# Epidemiological trends of tracheal, bronchus, and lung cancer at the global, regional, and national levels: a population-based study

**DOI:** 10.1186/s13045-020-00915-0

**Published:** 2020-07-20

**Authors:** Yujiao Deng, Peng Zhao, Linghui Zhou, Dong Xiang, Jingjing Hu, Yu Liu, Jian Ruan, Xianghua Ye, Yi Zheng, Jia Yao, Zhen Zhai, Shuqian Wang, Si Yang, Ying Wu, Na Li, Peng Xu, Dai Zhang, Huafeng Kang, Jun Lyu, Zhijun Dai

**Affiliations:** 1 Department of Breast Surgery, The First Affiliated Hospital, College of Medicine, Zhejiang University, Hangzhou, China; 2 Department of Oncology, The Second Affiliated Hospital of Xi’an Jiaotong University, Xi’an, China; 3 Department of Medical Oncology, The First Affiliated Hospital, College of Medicine, Zhejiang University, Hangzhou, China; 4 Celilo Cancer Center, Oregon Health Science Center affiliated Mid-Columbia Medical Center, The Dalles, OR USA; 5 Dana-Farber Cancer Institute, Harvard Medical School, Boston, MA USA; 6 Department of Radiotherapy, The First Affiliated Hospital, College of Medicine, Zhejiang University, Hangzhou, China; 7 Department of Clinical Research, The First Affiliated Hospital of Jinan University, Guangzhou, 510632 China

**Keywords:** Tracheal, bronchus, and lung cancer, Global burden of disease, Incidence, Death, Disability-adjusted life years

## Abstract

**Background:**

Investigations of disease incidence, mortality, and disability-adjusted life years (DALYs) are valuable for facilitating preventive measures and health resource planning. We examined the tracheal, bronchus, and lung (TBL) cancer burdens worldwide according to sex, age, and social development index (SDI) at the global, regional, and national levels.

**Methods:**

We assessed the TBL cancer burden using data from the Global Burden of Disease (GBD) database, including 21 regions, 195 countries, and territories in the diagnostic period 1990–2017. The data of TBL cancer-related mortality and DALYs attributable to all known risk factors were also analyzed. Age-standardized rates (ASRs) and their estimated annual percentage changes (EAPCs) were calculated.

**Results:**

Incident cases, deaths, and DALYs of TBL cancer increased worldwide (100.44%, 82.30%, and 61.27%, respectively). The age-standardized incidence rate (ASIR) was stable (EAPC = 0.02, 95% confidence interval [CI] − 0.03 to 0.08), but the age-standardized death (EAPC = − 0.34, 95%CI − 0.38 to − 0.3) and DALY rate decreased generally (EAPC = − 0.74, 95%CI − 0.8 to − 0.68). However, the change trend of ASIR and ASDR among sexes was on the contrary. China and the USA always had the highest incidence, mortality, and DALYs of TBL cancer. Significant positive correlations between ASRs and SDI were observed, especially among females. High (36.86%), high-middle (28.78%), and middle SDI quintiles (24.91%) carried the majority burden of TBL cancer. Tobacco remained the top cause of TBL cancer death and DALYs, followed by air pollution, the leading cause in the low-middle and low-SDI quintiles. Metabolic risk-related TBL cancer mortality and DALYs among females increased but was stable among males. The main ages of TBL cancer onset and death were > 50 years, and the DALYs concentrated in 50 − 69 years.

**Conclusions:**

To significantly reduce the growing burden of TBL cancer, treatment resources need to be skewed according to factors such as risks and geography, especially for high-risk groups and high-burden areas. Asia had the greatest TBL cancer burden, followed by high-income North America. Tobacco remains the leading cause of death and DALYs, followed by air pollution. Effective prevention measures against tobacco and air pollution should be strengthened.

## Background

Lung cancer was uncommon before the twentieth century, but it ranks the second and is the leading cause of cancer mortality. In addition, various factors play a crucial role in the occurrence, infiltration, metastasis of tracheal, bronchus, and lung (TBL) cancer, such as environmental and genetic factors [1, 2]. In recent years, substantial progress has been attained in terms of early diagnosis of and therapy options for TBL cancer [3]. However, the TBL cancer burden is still increasing owing to the aging population and risk factors such as pollution, dietary habits, and tobacco, which vary among different countries [4–6]. Further knowledge about TBL cancer burden is necessary to better allocate the limited health resources worldwide, which is helpful for the prevention, diagnosis, and treatment of TBL cancer.

The Global Burden of Disease (GBD) study included 354 diseases and injuries in 195 countries and territories worldwide, providing an opportunity to perform comprehensive assessments of disease incidence, mortality, disability-adjusted life years (DALY), and change trends of TBL cancer [7]. To better understand the TBL cancer burden among geographical locations, the social development index (SDI), age groups, and sexes, we conducted various subgroup analyses to assess the burden and variation trends of TBL cancer on the basis of data from the GBD study 2017.

## Methods

### Data resources

Annual data on TBL cancer were derived from the Global Health Data Exchange (GHDx) query tool (http://ghdx.healthdata.org/gbd-results-tool), including cases, deaths, and DALYs. The basic instructions of the GBD study and the methods for estimating the cancer burden have already been introduced in our previous studies [8–11]. More GBD 2017 study data information was listed in Additional file 1. Countries were divided by SDI into five categories (low, low-middle, middle, high-middle, and high) to assess the relationship between TBL cancer and social development status.

### Statistical analyses

We calculated estimated annual percentage change (EAPCs) of age-standardized rates (ASRs). The EAPC describes the ASR trends within a specified time interval. The natural logarithm of ASR is assumed to be linear along with time; that is, *Y = α + βX + ε*, where *Y* refers to ln (ASR), *X* the calendar year, and ε the error term. Based on this formula, *β* represents the positive or negative ASR trends. The EAPC was calculated as EAPC = 100 × (exp(*β*)-1). Its 95% confidence intervals (CI) could be obtained from the linear model. When the EAPC and lower CI limit are positive, ASR shows an upward trend. Conversely, when the EAPC and upper CI limit are negative, ASR shows a descending trend. In addition, we evaluated the relationship between SDI and ASRs in the different regions to define the potential factors that affect ASRs.

### Attributable risk factors

A comparative risk assessment framework was used to evaluate the disease burden attributable to 84 health risk factors categorized as behavioral, environmental/occupational, and metabolic risks. Exposures, attributable deaths, and DALYs of TBL cancer were estimated for 18 risk factors. Data of the risk factors were extracted from 46,000 empirical data points derived from cohort studies and randomized controlled trials.

## Results

### Global burden of TBL cancer

**Fig. 1:**
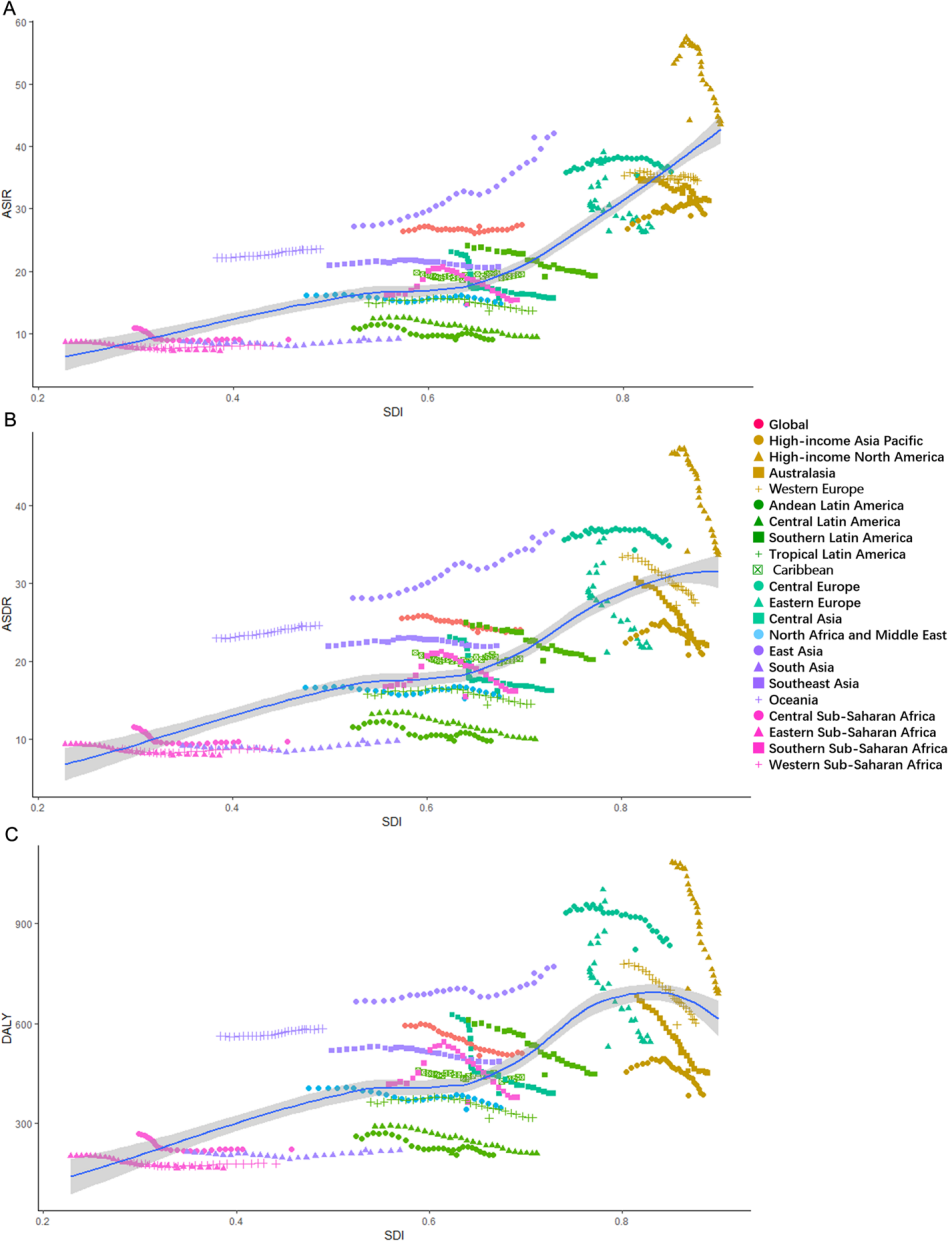
Age-standardized rates (per 100,000 population) of TBL cancer among regions based on SDI in 2017. **a** ASIR. **b** ASDR. **c** Age-standardized DALY rate. DALY: disability-adjusted life year; ASIR, age-standardized incidence rate; ASDR, age-standardized death rate; SDI, socio-demographic index

In 2017, the incident cases of TBL cancer increased to 2,163,130, twice the number in 1990. From 1990 to 2017, the TBL cancer deaths increased by 82.30%, and DALYs increased by 61.27% (Table 1). The age-standardized incidence rate (ASIR, per 100,000 persons) remained stable, while the age-standardized death rate (ASDR) and age-standardized DALY rate showed a decreasing trend (Table 2). In the further analysis, the ASIR among males decreased (EAPC = − 0.31; 95%CI, − 0.37 to − 0.24), while that among females increased (EAPC = 0.73; 95%CI, 0.67 to 0.79). ASRs among females showed an upward trend (EAPC = 0.31; 95%CI, 0.26 to 0.37). Burden was generally higher in males than in females, with a male-to-female ratio of 2–4:1. Positive correlations were found between ASIR, ASDR, and SDI (Fig. 1). Age-standardized DALY rate and SDI was positively related when SDI was < 0.8, and when SDI > 0.8, it reversed. However, a sex-related difference in this association was observed significantly (Additional file 2: Figure S1).

**Table 1:** The incidence, death, DALYs, and their change trends of tracheal, bronchus, and lung cancer from 1990 to 2017

Characteristics	Incident cases (No.)					Deaths (No.)					DALYs (No.)				
	1990		2017		1990–2017 increase (%)	1990		2017		1990–2017 increase (%)	1990		2017		1990–2017 increase (%)
	Both (95%UI) (No. × 10^**3**^)	Male/female ratio	Both (95%UI) (No. × 10^**3**^)	Male/female ratio		Both (95%UI) (No. × 10^**3**^)	Male/female ratio	Both (95%UI) (No. × 10^**3**^)	Male/female ratio		Both (95%UI) (No. × 10^**3**^)	Male/female ratio	Both (95%UI) (No. × 10^**3**^)	Male/female ratio	
Global	1079.19 (1060.31–1110.19)	2.77	2163.13 (2117.04–2212.89)	2.11	100.44	1032.94 (1014.7–1063.11)	2.79	1883.07 (1844.25–1922.81)	2.16	82.30	25379.25 (24887.6–26231.02)	2.90	40928.67 (40016.96–41855.08)	2.26	61.27
**Socio-demographic index**															
High SDI	504.53 (500.45–508.86)	2.62	797.42 (780.75–814.85)	1.68	58.05	462.6 (459.16–466.02)	2.68	635.08 (623.74–646.61)	1.75	37.28	10269.95 (10190.24–10349.09)	2.80	12166.63 (11940.27–12388.67)	1.79	18.47
High-middle SDI	291.66 (283.24–301.81)	3.26	622.56 (595.63–649.47)	2.31	113.46	285.77 (277.74–296.6)	3.19	537.22 (514.4–558.2)	2.33	87.99	7541.65 (7312.99–7837.67)	3.42	12131.49 (11619.86–12612.67)	2.48	60.86
Low SDI	33.12 (29.87–37.18)	3.66	68.43 (64.24–72.66)	2.74	106.61	33.48 (37.53–30.22)	3.78	70.78 (75.26–66.38)	2.86	111.43	885.24 (796.03–996.46)	3.41	1744.38 (1639.2–1857.13)	2.47	97.05
Low-middle SDI	58.87 (54.75–64.05)	3.02	121.20 (114.18–129.68)	2.50	105.87	59.34 (55.15–64.35)	3.04	124.51 (117.34–132.89)	2.51	109.84	1578.81 (1467.93–1718.73)	2.98	3086.99 (2902.02–3297.12)	2.47	95.53
Middle SDI	186.28 (179.16–197.22)	2.38	538.93 (510.94–569.42)	2.56	189.31	187.03 (179.93–198.03)	2.36	502.91 (479.64–528.68)	2.45	168.90	4980.32 (4776.3–5296.84)	2.41	11522.03 (10949.76–12140.59)	2.54	131.35
**Region**															
Andean Latin America	2.28 (2.13–2.42)	1.85	4.86 (4.40–5.33)	1.31	113.47	2.36 (2.2–2.51)	1.89	5.2 (4.71–5.69)	1.33	120.42	58 (54.2–61.82)	1.82	111.23 (100.12–122.25)	1.26	91.78
Australasia	8.50 (8.30–8.73)	2.59	15.33 (13.95–16.74)	1.45	80.21	7.48 (7.34–7.63)	2.68	10.96 (10.04–11.89)	1.48	46.42	163.25 (160.09–166.43)	2.63	209.62 (190.97–227.96)	1.43	28.41
Caribbean	5.23 (5.07–5.46)	2.60	9.86 (9.16–10.62)	1.91	88.36	5.52 (5.36–5.75)	2.61	10.22 (9.49–10.97)	1.89	85.04	124.05 (120.07–129.97)	2.54	221.59 (204.91–239.15)	1.88	78.63
Central Asia	11.71 (11.48–11.95)	4.20	11.83 (11.33–12.37)	3.83	1.01	11.53 (11.3–11.75)	4.07	11.87 (11.37–12.4)	3.82	2.95	326.91 (319.92–333.49)	4.51	313.61 (298.93–328.26)	3.88	− 4.07
Central Europe	55.56 (54.85–56.36)	4.93	74.37 (71.95–76.60)	2.54	33.85	55.04 (54.33–55.84)	4.80	73.02 (70.73–75.25)	2.58	32.67	1440.93 (1420.61–1461.4)	5.21	1660.32 (1608.05–1712.02)	2.68	15.23
Central Latin America	10.46 (10.32–10.62)	2.07	21.53 (20.67–22.43)	1.72	105.81	10.86 (10.72–11)	2.09	22.61 (21.73–23.53)	1.71	108.22	261.31 (257.87–264.83)	2.04	492.36 (472.52–514.08)	1.68	88.42
Central sub-Saharan Africa	2.52 (2.08–3.07)	3.14	4.53 (3.76–5.75)	2.72	79.89	2.54 (2.09–3.1)	3.19	4.57 (3.78–5.8)	2.72	80.16	68.35 (56.16–83.64)	3.05	123.99 (102.07–158.17)	2.75	81.39
East Asia	252.92 (241.56–269.56)	2.19	845.75 (809.40–883.11)	2.26	234.39	252.24 (241.19–268.92)	2.17	722.06 (690.73–751.27)	2.21	186.26	6655.77 (6345.73–7113.59)	2.21	15905.05 (15198.33–16566.97)	2.33	138.97
Eastern Europe	99.18 (96.51–102.05)	5.09	90.38 (87.33–93.56)	4.20	-8.87	92.82 (91.07–94.17)	5.03	73.24 (71.71–74.77)	4.49	-21.10	2477.97 (2423.42–2519.25)	6.02	1784.52 (1743.68–1825.11)	5.09	− 27.98
Eastern sub-Saharan Africa	6.27 (5.49–7.10)	3.68	10.49 (9.70–11.52)	2.88	67.39	6.47 (5.69–7.32)	3.79	10.94 (10.14–12.06)	2.97	69.24	162.1 (140.8–184.8)	3.45	267.66 (247.43–295.11)	2.69	65.12
High-income Asia Pacific	54.81 (54.19–55.42)	2.65	135.16 (129.85–140.27)	2.37	146.61	45.47 (44.96–45.91)	2.70	100.62 (97.46–103.72)	2.36	121.31	958.74 (946.19–969.67)	2.83	1588.44 (1532.76–1642.61)	2.71	65.68
High-income North America	189.38 (186.97–191.84)	1.72	273.20 (265.26–280.84)	1.19	44.26	167.42 (165.84–168.96)	1.77	212.19 (207.06–217.68)	1.24	26.74	3685.79 (3649–3721.25)	1.79	4183.22 (4074.76–4295.94)	1.29	13.50
North Africa and Middle East	29.16 (26.75–31.98)	5.32	60.44 (57.18–63.76)	4.21	107.28	28.82 (26.52–31.6)	5.32	60.97 (57.61–64.09)	4.29	111.55	786.78 (715.61–862.12)	5.15	1511.45 (1428.41–1590.68)	4.09	92.11
Oceania	0.68 (0.57–0.88)	3.37	1.55 (1.27–2.09)	3.18	128.29	0.66 (0.56–0.86)	3.36	1.52 (1.26–2.04)	3.17	129.72	19.51 (16.31–25.57)	3.48	44.10 (35.58–60.27)	3.27	126.00
South Asia	51.88 (48.00–56.20)	3.98	122.60 (115.07–130.73)	2.68	136.30	52.17 (48.21–56.52)	4.07	126.52 (118.81–134.6)	2.75	142.52	1402.61 (1296.45–1516.88)	3.80	3131.09 (2942.45–3331.08)	2.53	123.23
Southeast Asia	55.29 (51.43–60.30)	2.34	119.50 (109.30–130.96)	2.47	116.14	55.58 (51.73–60.74)	2.33	123.25 (112.82–135.24)	2.43	121.72	1489.8 (1380.76–1629.82)	2.36	3005.25 (2747.8–3294.35)	2.54	101.72
Southern Latin America	11.51 (11.23–11.78)	4.15	15.64 (14.47–16.92)	1.95	35.91	11.85 (11.57–12.14)	4.05	16.48 (15.27–17.84)	1.93	39.04	292.98 (285.73–300.47)	4.40	356.99 (328.99–388.02)	2.00	21.84
Southern sub-Saharan Africa	4.64 (4.29–5.57)	2.49	8.17 (7.80–8.57)	2.22	75.88	4.67 (4.3–5.6)	2.42	8.37 (7.98–8.76)	2.16	79.03	126.45 (116.97–149.16)	2.66	213.18 (202.83–224.31)	2.41	68.59
Tropical Latin America	14.03 (13.72–14.29)	2.21	31.66 (30.91–32.50)	1.40	125.66	14.33 (14.03–14.58)	2.21	33.17 (32.4–34.02)	1.41	131.49	364.9 (357.25–371.65)	2.20	750.05 (732.31–769.16)	1.36	105.55
Western Europe	206.08 (203.92–208.73)	3.80	292.86 (280.87–304.68)	1.89	42.11	197.7 (195.81–199.63)	3.73	241.32 (232.78–250.12)	1.99	22.06	4339.98 (4297.62–4385.22)	4.02	4725.7 (4554.54–4906.84)	1.99	8.89
Western sub-Saharan Africa	7.11 (6.13–8.39)	3.22	13.44 (11.62–15.80)	2.53	89.03	7.4 (6.39–8.72)	3.22	13.97 (12.1–16.39)	2.58	88.77	173.08 (148.12–205.18)	3.13	329.25 (283.13–388.81)	2.36	90.23

*DALY* disability-adjusted life year, *UI* uncertainty interval, *SDI* socio-demographic index

**Table 2:** The ASRs and variations of tracheal, bronchus, and lung cancer from 1990 to 2017

Characteristics	ASIR (per 100,000 persons)					ASDR (per 100,000 persons)					Age-Standardized DALY Rate (per 100,000 persons)				
	1990 (95%UI)		2017 (95%UI)		EAPC (95%CI)	1990 (95%UI)		2017 (95%UI)		EAPC(95%CI)	1990 (95%UI)		2017 (95%UI)		EAPC (95%CI)
	Both	Male/female ratio	Both	Male/female ratio		Both	Male/female ratio	Both	Male/female ratio		Both	Male/female ratio	Both	Male/female ratio	
Global	26.34 (25.89–27.05)	3.26	27.13 (26.55–27.75)	2.45	0.02 (-0.03–0.08)	25.54 (25.1–26.25)	3.33	23.74 (23.25–24.24)	2.54	− 0.34 (− 0.38–− 0.3)	594.04 (582.83–613.2)	3.23	503.05 (491.93–514.34)	2.48	− 0.74 (− 0.8–− 0.68)
**Socio-demographic index**															
High SDI	39.07 (38.76–39.4)	3.47	36.25 (35.5–37.04)	2.00	− 0.41 (-0.49–− 0.33)	35.57 (35.32–35.84)	3.62	28.29 (27.77–28.81)	2.14	− 0.97 (− 1.04–− 0.9)	817.66 (811.1–823.94)	3.41	593.07 (582.05–604.07)	1.98	− 1.33 (− 1.39–− 1.27)
High-middle SDI	29.91 (29.06–30.98)	3.99	34.46 (32.94–35.93)	2.74	0.4 (0.25–0.55)	29.81 (28.98–30.94)	4.00	29.99 (28.74–31.15)	2.82	− 0.12 (− 0.22–− 0.01)	749.67 (726.97–778.93)	3.97	656.82 (628.76–682.81)	2.80	− 0.72 (− 0.85–− 0.59)
Low SDI	10.14 (9.16–11.29)	3.56	10.08 (9.46–10.72)	3.05	0.02 (− 0.21–0.26)	10.71 (9.68–11.93)	3.64	10.82 (10.12–11.51)	3.17	0.08 (− 0.17–0.33)	245.45 (221.25–275.57)	3.36	235.41 (220.98–250.32)	2.74	− 0.11 (− 0.32–0.11)
Low-middle SDI	10.31 (9.59–11.21)	3.00	10.41 (9.81–11.13)	2.73	0.03 (0.01–0.06)	10.79 (10.04–11.7)	3.01	11.01 (10.38–11.73)	2.75	0.08 (0.06–0.11)	254.35 (236.68–276.55)	2.97	247.18 (232.55–263.93)	2.68	− 0.13 (− 0.16–− 0.1)
Middle SDI	19.12 (18.41–20.23)	2.52	24.76 (23.5–26.16)	2.83	1.04 (0.95–1.13)	19.9 (19.16–21.03)	2.52	23.46 (22.38–24.65)	2.73	0.72 (0.67–0.77)	472.34 (453.76–501.45)	2.49	503.86 (478.95–530.3)	2.73	0.25 (0.2–0.31)
**Region**															
Andean Latin America	10.9 (10.17–11.62)	2.03	9.07 (8.21–9.94)	1.42	− 0.78 (− 0.98–− 0.59)	11.6 (10.8–12.36)	2.06	9.76 (8.86–10.67)	1.45	− 0.73 (− 0.93–− 0.54)	259.62 (242.39–276.94)	1.97	204.07 (183.98–224.59)	1.35	− 0.99 (− 1.19–− 0.79)
Australasia	34.96 (34.13–35.85)	3.10	31.69 (28.91–34.62)	1.60	− 0.4 (− 0.47–− 0.34)	30.6 (30.01–31.21)	3.28	22.32 (20.41–24.23)	1.66	− 1.32 (− 1.38–− 1.25)	682.37 (668.99–695.83)	2.93	456.72 (415.5–496.96)	1.52	− 1.65 (− 1.72–− 1.58)
Caribbean	19.82 (19.23–20.67)	2.80	19.35 (18–20.86)	2.17	0.02 (− 0.05–0.09)	21.11 (20.5–21.96)	2.82	20.08 (18.64–21.56)	2.18	− 0.06 (− 0.13–0.02)	458.68 (444.02–480.37)	2.71	433.97 (401.41–468.43)	2.08	− 0.15 (− 0.2–− 0.09)
Central Asia	23.09 (22.64–23.54)	5.63	15.63 (14.98–16.3)	4.97	− 1.5 (− 1.73–− 1.27)	23.09 (22.65–23.51)	5.58	16.12 (15.47–16.78)	5.04	− 1.36 (− 1.59–− 1.14)	625.67 (613.17–637.9)	5.75	388.11 (370.76–405.12)	4.82	− 1.87 (− 2.08–− 1.65)
Central Europe	35.9 (35.44–36.39)	6.14	35.42 (34.3–36.51)	3.14	0 (− 0.11–0.11)	35.56 (35.11–36.07)	6.10	34.3 (33.25–35.35)	3.28	− 0.09 (− 0.18–0)	933.31 (920.48–946.37)	6.17	822.54 (796.38–847.48)	3.10	− 0.47 (− 0.57–− 0.37)
Central Latin America	12.35 (12.2–12.52)	2.30	9.4 (9.02–9.79)	2.03	− 1.25 (− 1.33–− 1.17)	13.17 (13–13.34)	2.32	9.97 (9.59–10.38)	2.03	− 1.27 (− 1.35–− 1.18)	288.7 (284.86–292.51)	2.24	208.83 (200.51–218.03)	1.95	− 1.44 (− 1.51–− 1.36)
Central sub-Saharan Africa	10.95 (9.14–13.24)	3.50	9.1 (7.6–11.41)	3.31	− 0.78 (− 0.94–− 0.62)	11.58 (9.66–13.96)	3.51	9.63 (8.05–12.06)	3.33	− 0.77 (− 0.94–− 0.61)	267.73 (220.82–326.5)	3.48	222.19 (183.56–281.77)	3.28	− 0.8 (− 0.96–− 0.64)
East Asia	27.24 (26.06–28.98)	2.31	41.54 (39.8–43.37)	2.41	1.66 (1.53–1.79)	28.11 (26.89–29.93)	2.32	35.94 (34.41–37.34)	2.38	1.02 (0.95–1.09)	665.67 (635.14–711.27)	2.24	751.11 (718.1–782.06)	2.39	0.42 (0.31–0.52)
Eastern Europe	33.44 (32.57–34.35)	8.45	26.33 (25.44–27.21)	6.48	− 1.23 (− 1.48–− 0.98)	31.31 (30.71–31.75)	8.70	21.1 (20.66–21.55)	7.32	− 1.88 (− 2.12–− 1.63)	838.2 (819.27–852.15)	9.16	529.08 (516.65–541.2)	7.21	− 2.18 (− 2.46–− 1.91)
Eastern sub-Saharan Africa	8.64 (7.63–9.72)	3.86	7.21 (6.68–7.9)	3.44	− 0.8 (− 0.87–− 0.73)	9.3 (8.26–10.43)	3.91	7.83 (7.26–8.6)	3.54	− 0.75 (− 0.82–− 0.69)	201.39 (176.39–228.78)	3.70	164.8 (152.48–181.65)	3.21	− 0.9 (− 0.98–− 0.81)
High-income Asia Pacific	26.83 (26.54–27.15)	3.62	28.92 (27.77–30.06)	3.21	0.25 (0.1–0.4)	22.41 (22.16–22.63)	3.74	20.77 (20.1–21.43)	3.34	− 0.42 (− 0.62–− 0.23)	455.56 (449.69–460.72)	3.55	382.8 (368.97–396.18)	3.13	− 0.8 (− 0.99–− 0.6)
High-income North America	53.24 (52.57–53.91)	2.22	44.22 (42.92–45.51)	1.41	− 1.02 (− 1.24–− 0.81)	46.67 (46.24–47.1)	2.33	34.09 (33.25–34.99)	1.50	− 1.44 (− 1.6–− 1.28)	1083.68 (1073.28–1094.01)	2.15	702.16 (683.25–721.43)	1.45	− 1.92 (− 2.07–− 1.78)
North Africa and Middle East	16.16 (14.93–17.74)	5.18	14.64 (13.83–15.42)	4.30	− 0.23 (− 0.34–− 0.12)	16.6 (15.32–18.26)	5.18	15.23 (14.39–16)	4.37	− 0.16 (− 0.27–− 0.04)	404.46 (370.85–443.47)	5.02	340.62 (321.82–358.68)	4.15	− 0.52 (− 0.63–− 0.41)
Oceania	22.19 (19.05–28.36)	3.03	23.56 (19.68–31.02)	2.91	0.26 (0.25–0.28)	22.95 (19.74–29.32)	3.02	24.59 (20.68–32.06)	2.89	0.3 (0.28–0.31)	562.62 (476.64–728.7)	3.12	583.26 (483.35–784.81)	2.97	0.19 (0.16–0.21)
South Asia	8.69 (8.01–9.42)	3.77	9.19 (8.64–9.81)	2.88	0.21 (0.04–0.38)	9.12 (8.4–9.89)	3.83	9.77 (9.16–10.4)	2.96	0.25 (0.07–0.43)	213.17 (196.68–230.95)	3.63	219.2 (205.77–233.26)	2.67	0.1 (− 0.06–0.26)
Southeast Asia	20.95 (19.53–22.83)	2.62	20.67 (18.93–22.72)	2.93	− 0.12 (− 0.2–− 0.05)	21.89 (20.45–23.87)	2.61	21.94 (20.1–24.18)	2.93	− 0.05 (− 0.13–0.03)	518.32 (481.9–566.49)	2.63	484.12 (443.23–530.59)	2.92	− 0.35 (− 0.42–− 0.28)
Southern Latin America	24.12 (23.56–24.69)	5.04	19.12 (17.69–20.7)	2.44	− 0.89 (− 0.94–− 0.84)	24.95 (24.37–25.54)	4.99	20.01 (18.53–21.66)	2.48	− 0.84 (− 0.88–− 0.79)	610.41 (595.3–626.01)	5.15	444.97 (410.02–483.38)	2.37	− 1.23 (− 1.28–− 1.18)
Southern sub-Saharan Africa	16.16 (14.85–19.48)	3.08	14.69 (14.03–15.39)	3.03	− 0.51 (− 0.98–− 0.03)	16.7 (15.28–20.09)	3.04	15.4 (14.7–16.12)	3.00	− 0.43 (− 0.88–0.03)	415.68 (383.33–495.34)	3.19	362.31 (344.91–380.44)	3.13	− 0.72 (− 1.26–− 0.18)
Tropical Latin America	14.95 (14.62–15.22)	2.52	13.64 (13.31–14.01)	1.74	− 0.42 (− 0.56–− 0.28)	15.73 (15.4–15.99)	2.54	14.44 (14.1–14.8)	1.78	− 0.39 (− 0.53–− 0.25)	363.48 (356.03–370.07)	2.48	315.16 (307.69–323.21)	1.63	− 0.62 (− 0.78–− 0.46)
Western Europe	35.36 (34.98–35.81)	5.11	34.11 (32.7–35.49)	2.16	− 0.12 (− 0.16–− 0.07)	33.43 (33.11–33.75)	5.16	27.2 (26.23–28.2)	2.36	− 0.76 (− 0.79–− 0.72)	777.62 (770.03–785.76)	4.94	595.84 (573.77–619.39)	2.15	− 1 (− 1.03–− 0.96)
Western sub-Saharan Africa	8.21 (7.1–9.66)	3.15	8.06 (6.98–9.41)	2.85	0.07 (− 0.04–0.18)	8.8 (7.6–10.32)	3.17	8.71 (7.55–10.14)	2.86	0.11 (− 0.01–0.22)	185.79 (159.72–219.77)	3.02	178.33 (154.13–209.79)	2.72	− 0.03 (− 0.15–0.08)

*ASR* age standardized death rate, *ASDR* age-standardized death rate, *ASIR* age-standardized incidence rate, *DALY* disability-adjusted life year, *EAPC* estimated annual percentage change, *CI* confidence interval, *UI* uncertainty interval, *SDI* socio-demographic index

From 1990 to 2017 (Additional file 3: Figure S2), the > 70 years age group presented a significant increase with time, whereas the 15–49 years and 50–69 years age groups showed a slight decrease. The > 50 years age subgroup carried the majority of incidence and mortality. The main age at TBL cancer onset was concentrated in 50–69 years. Most deaths occurred at ages > 70 years, followed by 50–69 years. In the past 28 years, most DALYs were in the 50–69-year subgroup.

**Fig. 2:**
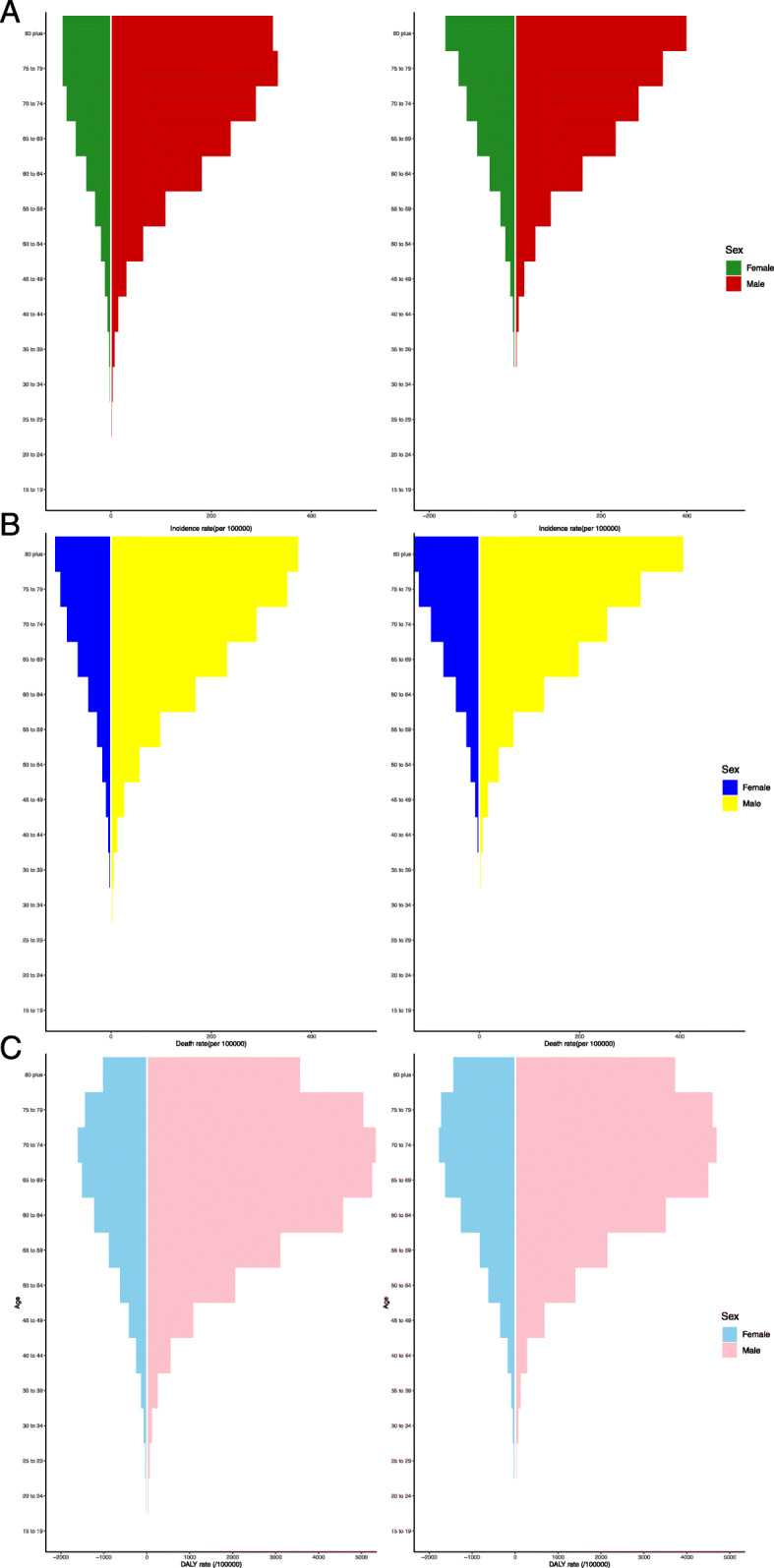
The incidence, death, and DALY rates of TBL cancer among gender and age. **a** Incidence. **b** Death rate. **c** DALY rate. DALY: disability-adjusted life year

In the subgroup analysis of gender, the main age of TBL cancer incident cases and deaths among females was early than that among males. As presented in Fig. 2, the morbidity and mortality of TBL cancer increased with age. As for the DALY rate, the patients were always mainly concentrated in the 60–79-year age group.

### Global burden of TBL cancer among countries

From 1990 to 2017 (Additional file 4: Table S1), China and the USA always had the highest TBL cancer burden in both genders. And those were always lower in Antigua, Barbuda, and the Marshall Islands. In 1990, females in UK and males in Russia had a higher TBL cancer burden than other countries. Up to 2017, the countries with greatest increase of TBL cancer burden were the United Arab Emirates and Qatar, while Kazakhstan had the greatest decrease.

**Fig. 3:**
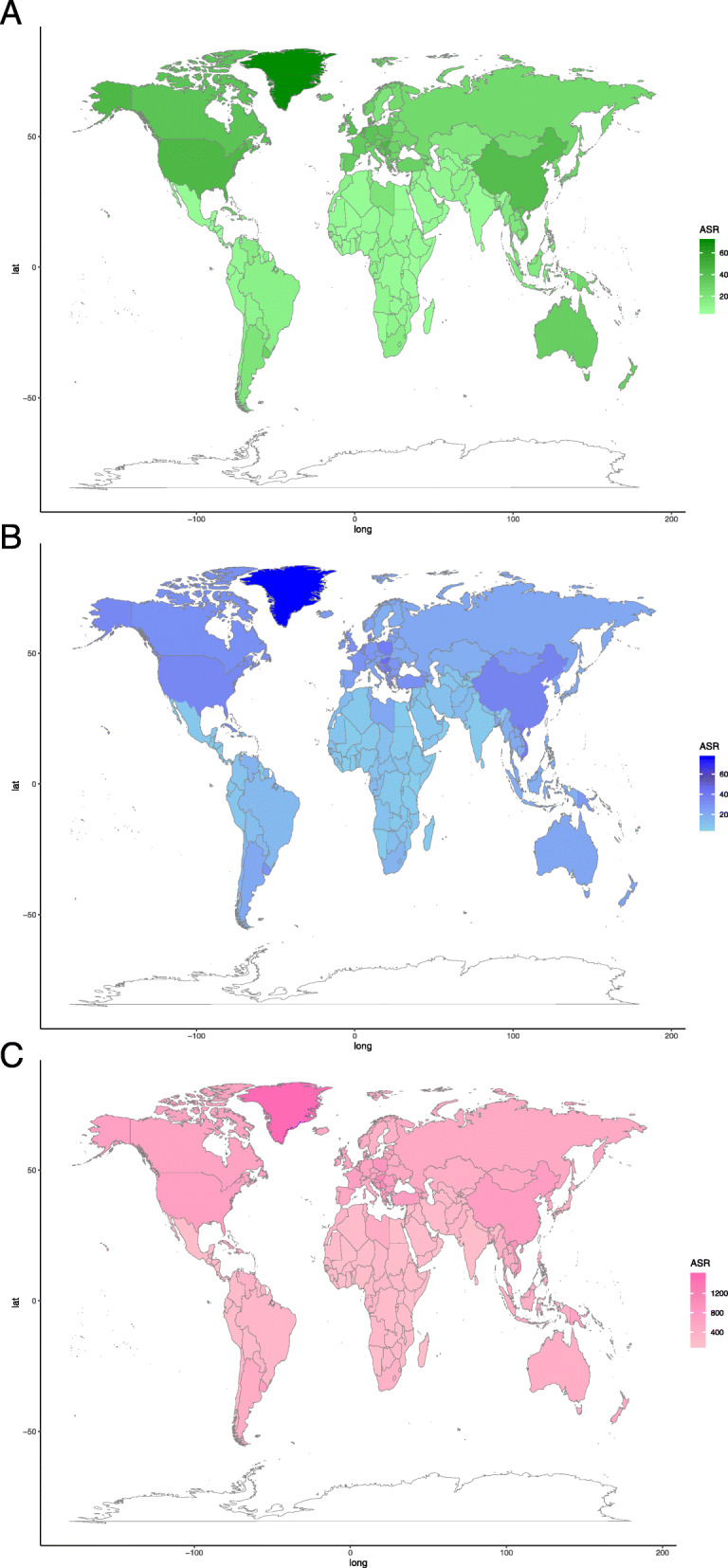
The ASRs (per 100,000 population) of TBL cancer incidence, death, DALY in 2017 worldwide. **a** ASIR. **b** ASDR. **c** Age-standardized DALY rate. DALY: disability-adjusted life year; ASIR, age-standardized incidence rate; ASDR, age-standardized death rate

ASRs and their EAPCs among countries were presented detailed in Fig. 3 and Tables S2-3 (Additional files 5 and 6), respectively. People in Greenland among both genders always had the highest ASRs over past 28 years. However, from 1990 to 2017, country with the lowest ASRs had changed from Uganda to Malawi (Saudi Arabia to Maldives among females, and from Uganda to Nicaragua among males) (Additional file 4: Table S1). Females in Spain and France, and males in Georgia had a faster increase of ASRs. Besides, ASRs of China males also increased rapidly. But ASRs in Bahrain, Maldives, and Kazakhstan declined at a relatively rapid rate.

### Global burden of TBL cancer among regions

The top 3 regions with the greatest TBL cancer burden in both genders remained East Asia, Western Europe, and high-income North America over 28 years; Oceania, Andean Latin America, and Central sub-Saharan Africa always had the lower burden (Additional file 4: Table S1). As for the ASRs, from 1990 to 2017, high-income North America, East Asia, and Western Europe always had higher ASRs of TBL cancer among both sexes. Besides, the ASRs were also higher among females in Australasia, Central Europe, and among males in Eastern and Central Europe. Western sub-Saharan Africa, Eastern sub-Saharan Africa, and South Asia always had the lowest ASRs. East Asia and South Asia had faster increase of TBL cancer burden, whereas Eastern Europe had the fastest decrease. The ASRs increased most in East Asia, but it decreased most in Eastern Europe and Central Asia.

### Global burden of TBL cancer among the SDI quintiles

In the past 28 years, the high SDI quintile always had the highest incident cases, deaths, and DALYs of TBL cancer, while the low SDI quintile always had the lowest. Up to 2017, the fastest increase of TBL cancer burden was in the middle SDI quintile, and the slowest increase was in the high SDI quintile (Table 1).

As for ASRs, ASIR in high SDI quintile remained the highest, while that in low SDI quintile was always the lowest. The SDI quintile with the highest ASDR or age-standardized DALY rate had changed from the high to high-middle SDI quintile, but they kept the lowest in the low SDI quintile. All the ASRs decreased most in high SDI quintile, but they increased most in low SDI quintile (EAPC = 1.04, 0.72, and 0.25, respectively). ASIR showed a downward trend only in high SDI quintile, and ASDR decreased in the high and high-middle SDI quintiles. However, the age-standardized DALY rate in all SDI quintiles presented a decreasing trend except for the low SDI quintile (Table 2).

### Attributable risks

**Fig. 4:**
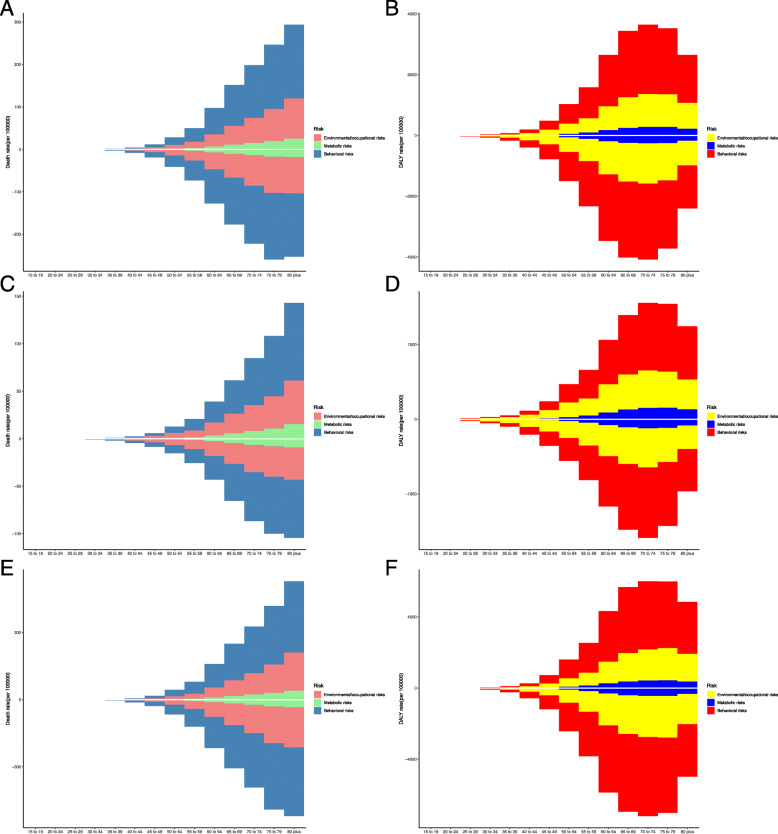
The death and DALY rate of TBL cancer by age, gender, and risk factors. The upper column in each group is data in 2017 and the lower column in 1990. **a** Death rate among both sexes. **b** DALY rate among both sexes. **c** Death rate among females. **d** DALY rate among females. **e** Death rate among males. **f** DALY rate among males. DALY: disability-adjusted life year

As shown in Fig. 4, behavioral risks kept the leading cause of death and DALY rate of TBL cancer in both gender and all age subgroups (from 15–19 to 80 plus years old), followed by environmental/occupational risks, and the metabolic risk proportion were the least. The contribution of all risk factors to death rate of TBL cancer increased with age. However, DALY rates increased with age until age of 70–74 years and then declined thereafter.

### Distribution of total risk factors among the regions

As shown in Figure S3 (Additional file 7), behavioral risks related ASDR and age-standardized DALY rate increased most in East Asia but decreased most in high-income North America over 28 years among males; as for females, they increased most in Central Europe but decreased most in high-income North America. Environmental/occupational risks related ASDR and age-standardized DALY rate decreased generally, and ASDR showed a downward trend among males except for East Asia; as for females, they all increased in South Asia, Southern Latin America, Western Europe, Central Europe, and Australasia but decreased in other regions.

### Distribution of six risk factors among SDI quintiles

**Fig. 5:**
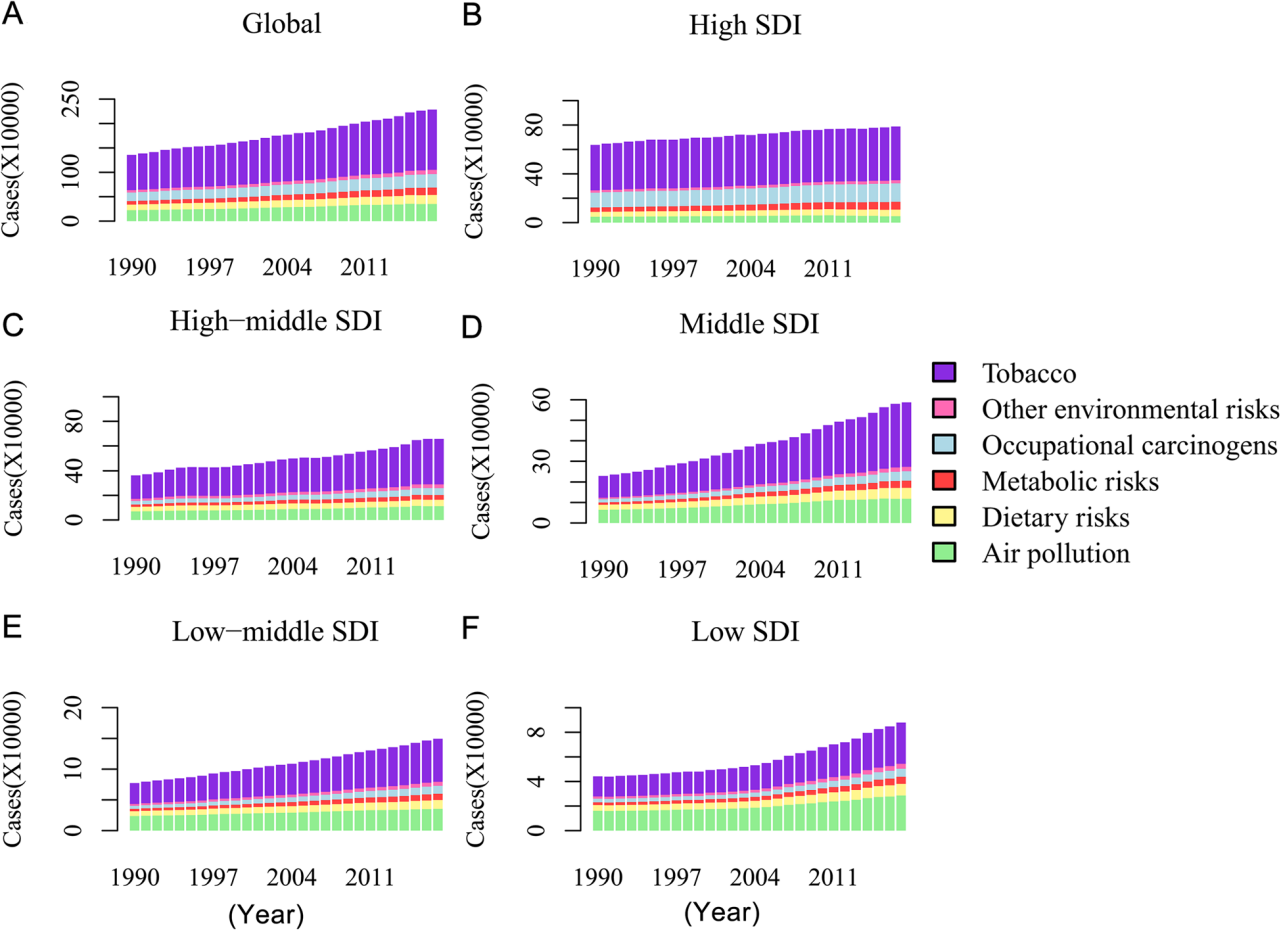
The change trends of TBL cancer DALYs among SDI quintiles and risks over 28 years. DALY: disability-adjusted life year; SDI, socio-demographic index

**Fig. 6:**
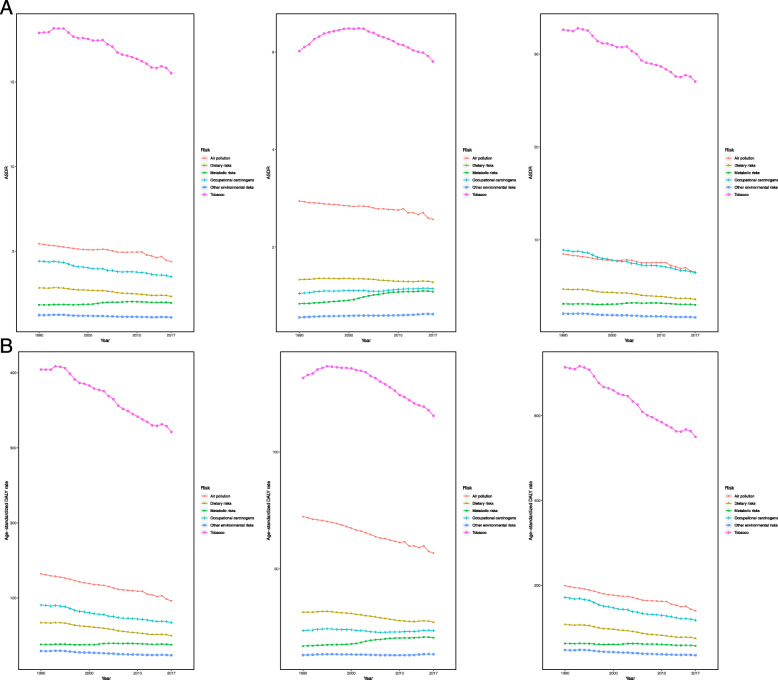
The change trends of ASRs for TBL cancer among sexes and risk factors. **a** ASDR, **b** age-standardized DALY rate. ASDR, age-standardized death rate; ASR, age-standardized rate; DALY: disability-adjusted life year

As shown in Fig. 5 and Figure S4 (Additional file 8), the deaths and DALYs among the five SDI quintiles showed a steady upward trend, and all six risk factors increased with time. In the past 28 years, tobacco remained the leading cause of TBL cancer deaths and DALYs, followed by air pollution, occupational carcinogens, dietary risks, metabolic risks, and other environmental risks. In the high SDI quintile, the leading three risk factors were tobacco, occupational carcinogens, and metabolic risks. In the middle, low-middle, and low SDI quintiles, tobacco, air pollution, dietary risks, occupational carcinogens, metabolic risks, and other environmental risks were ranked in order of risk among deaths and DALYs from high to low. As for the ASRs (Fig. 6), all the risk factors showed a decreasing trend with time among males, except for metabolic risks with a stable trend. Besides, the air pollution and occupational carcinogen-related ASRs and their trends were similar. However, for females, tobacco-related ASRs increased initially and then decreased. Metabolic risks showed an obvious increasing trend. Air pollution-related ASRs were high and kept decreasing among females, second to tobacco.

## Discussion

At present, lung cancer continues to be a major global public health problem. The ASIR was stable globally, but the ASDR and age-standardized DALY rate decreased generally. However, the change trend of ASIR and ASDR among sexes was on the contrary. ASIR and ASDR of TBL cancer in females showed an increasing trend, which is contrary to males. Therefore, though males carried the majority burden of TBL cancer, we should attach importance to the higher growth rate of women and its related risk factors among various regions [12]. The burden of lung cancer in men was reported largely determined by smoking patterns, although other factors such as air pollution and occupational exposure also play a role. While the burden of lung cancer in women was found to be associated primarily with smoking patterns, it is also associated with other risk factors, including air pollution and occupational exposure et al. [13]. Especially in East Asia, where smoking by women remained uncommon, indoor air pollution from cooking and heating played a major role in lung cancer incidence [14]. Age-standardized DALY rate of TBL cancer kept decreasing in both genders worldwide, which might due to the improvement of lung cancer treatment [15, 16] and variation of related risk factors, which was consistent with the global disease trend [17].

The burden and its trend of TBL cancer varied among regions. East Asia, Western Europe, and high-income North America (Canada and USA), where smoking uptake began earliest, had higher burden of TBL cancer, which might result from historical smoking patterns [18] and epidemic [12]. Indeed, lung cancer mortality began to increase 20 to 30 years after the onset of widespread smoking, and peak 30 to 40 years after the peak of smoking in the population [19]. Burdens were imbalanced among the five SDI quintiles, which might result from the inequalities in access to health care [20–22]. At present, one third of the TBL cancer burden was in the high SDI quintile, where ASRs decreased most, which might benefit from the advanced medical conditions [23, 24]. A previous study in California showed that in the high SDI quintile, the TBL burden among males decreased [25]. All indicators were always lowest in the low SDI quintile. However, data in low SDI quintile is scarce, and the detected trends should be treated cautiously.

China and the USA always had the highest burden of TBL cancer, which might be partly due to their high population. Females in the UK and males in Russia also bore a great TBL cancer burden. It is reported that the mortality of lung cancer declined in the USA, benefiting from the decline in smoking rates and clean air legislation [26]. In 2017, Greenland and Hungary had higher ASRs than other districts, whereas Malawi had the lowest. Consistent with the previous data, the ASIR and ASDR of lung cancer in Hungary were higher than those in Western-European countries [27]. Another study also stated that the incidence and survival rates of lung cancer in Greenland were comparable to those in northern European countries [28]. In addition, females in Spain and France, and males in Georgia and China had a faster increase of ASRs, which deserves further investigation. In Europe, lung cancer rates were falling for smoking men and rising for smoking women overtime [29]. Previous studies indicated that female heavy smokers had a higher risk of lung cancer compared to men [30, 31]. The gender difference may stem from differences in the number of men and women who smoke and how their bodies react to tobacco. In addition to smoking and other sex-related factors that may increase a woman’s susceptibility to lung cancer, such as genetic susceptibility, sex hormone exposure [32, 33], and molecular characteristics [34]. However, the biological basis of gender differences is controversial and requires further evaluation.

ASRs were also higher in Australasia [35], Europe, and East Asia. South Asia, Western, and Eastern sub-Saharan Africa had the lowest ASRs. The huge differences in TBL cancer morbidity and mortality among sexes, countries, and regions remind us that the government should investigate in-depth the reasons such as genetic factors, risk factors, policy adoption, and medical technology. A previous study described that the difference in lung cancer mortality between genders in Latin America was attributable to smoking patterns [36]. The burden of lung cancer varied in different countries and regions partially result from the gap of health-care resources [37–39], leading to different opportunities for diagnosis and treatment outcomes.

The leading cause of death and DALYs was behavioral risks (including smoking, secondhand smoke, and low-fruit diet), followed by environmental/occupational and metabolic risks (high-fasting plasma glucose level). The contribution of all the risk factors to death increased with age, which showed an accumulative effect [40]. In high-income North America and Asia, the government should take necessary measures to alter the impact of behavioral risks on ASDR. The mortality rates of lung cancer were high in countries where smoking uptake began earliest, especially in North America and Europe [41]. In the low-middle and low SDI quintiles, the top 3 risk factors of TBL cancer deaths and DALYs were air pollution, tobacco, and dietary risks (low-fruit diet). In China, high levels of fine particulate matter (PM2.5) might attribute to its huge TBL cancer burden [26, 42]. Measures to prevent and control home ambient particulate matter pollution and household air pollution from solid fuels should be further strengthened [43], especially in less developed areas. ASRs attributable to tobacco among females increased until 2013 and then decreased, which might have resulted from the period of smoking cessation. Women started quitting smoking mostly in the 1980s, which was later than the anti-smoking movement by the US Department of Health in 1964 [12]. For males, the effect of tobacco on the prognosis of patients with TBL cancer has diminished, consistent with the previous cigarette epidemic [18]. These changes are largely due to the tobacco control worldwide. Lung cancer mortality rates began to increase in the population 20 to 30 years after widespread smoking began [12]. Although tobacco control was popular in the past 50 years, the decline in smoking rates may have stalled at the current levels.

As regards metabolic risks (high-fasting plasma glucose level), the prognosis in female patients showed an increasing trend, whereas that of males was stable. Metabolic risk-related [44] ASDR increased in most regions, indicating fasting glucose level elevated TBL cancer death. Some studies provided evidence that diabetes correlated with an elevated risk of lung cancer mortality [45, 46], and baseline fasting plasma glucose level was an independent predictor of lung cancer survival [47]. Another large prospective study revealed that pre-existing diabetes was related to the poor prognosis in women with lung cancer [48]. A meta-analysis suggested that diabetics patients have an increased risk of lung cancer, especially women [49], similar to another study [50]. The mechanisms of the difference between genders, such as hormonal and environmental levels, deserve further investigation.

The main strength of this study is that we presented a comprehensive review of the TBL cancer burden based on the most recent national estimated data worldwide. However, this study also has some limits. Owing to the breadth and complexity of the data, the TBL cancer burden should be interpreted with caution. Few data are available from countries with lower SDI values, and the burden may be underestimated owing to the different levels of registration management. Although the GBD study data are considered of high quality, the accuracy of cancer information collected, extracted, and reported in population-based cancer registries must be improved. Owing to the lack of specific information such as TBL cancer classification, staging, and treatment, further analysis is difficult to achieve.

## Conclusions

Our study provides a comprehensive overview of the global TBL cancer burden. Incidence, mortality, DALYs, ASRs, and their trends varied substantially by gender, age, socioeconomic status, ethnicity, and geography. The incident cases, mortality, and DALYs of TBL cancer kept increasing worldwide. Asia had the greatest TBL cancer burden, followed by high-income North America. The leading cause of death and DALYs in TBL cancer was tobacco, followed by air pollution. Further investigation is warranted to determine the causes of these changes.

## Supplementary information

**Additional file 1.** GBD overview.

**Additional file 2: Figure S1.** Age-standardized rates (per 100,000) of TBL cancer among regions based on SDI in 2017. **Figure legends:** (a) ASIR among females, (b) ASIR among males, (c) ASDR among females, (d) ASDR among males, (e) Age-standardized DALY rate among females, (f) Age-standardized DALY rate among males. ASIR, age-standardized incidence rate; ASDR, age-standardized death rate; DALY: disability adjusted life-year; SDI, socio-demographic index.

**Additional file 3: Figure S2.** The proportion of different age subgroups in TBL cancer burden by years. **Figure legends:** (a) incident cases, (b) deaths and (c) DALYs. DALY: disability adjusted life-year.

**Additional file 4: Table S1.** Three countries/regions with top and bottom burden of tracheal, bronchus, and lung cancer.

**Additional file 5: Table S2.** The incidence, deaths, DALYs and variations of TBL cancer from 1990 to 2017 among countries.

**Additional file 6: Table S3.** The ASRs and variations of TBL cancer from 1990 to 2017 among countries.

**Additional file 7: Figure S3.** The ASDR and DALY of TBL cancer among different regions, genders and risk factors. **Figure legends:** The left column in each group is data in 1990 and the right column in 2017. (a) ASDR among both sexes; (b) Age standardized DALY rate among both sexes; (c) ASDR among female; (d) Age standardized DALY rate among female; (e) ASDR among male; (f) Age standardized DALY rate among male. ASDR, Age standardized death rate; DALY: disability adjusted life-year.

**Additional file 8: Figure S4.** The change trends of TBL cancer deaths among SDI quintiles and risks over 28 years. **Figure legends:** SDI, socio-demographic index.

## Data Availability

The datasets supporting the conclusions of this article are included within the article (and its additional files).

## Abbreviations

ASR: Age-standardized rate
ASDR: Age standardized death rate
ASIR: Age standardized incidence rate
CI: Confidence interval
DALY: Disability-adjusted life year
EAPC: Estimated annual percentage change
GBD: Global Burden of Disease
GHDx: Global Health Data Exchange
UI: Uncertainty interval
PM2.5: Fine particulate matter
SDI: Socio-demographic index
TBL cancer: Tracheal, bronchus, and lung cancer
