# Supplementary material for: Epidemiological trends of tracheal, bronchus, and lung cancer at the global, regional, and national levels: a population-based study
Source: J Hematol Oncol. 2020 Jul 20;13:98. doi: 10.1186/s13045-020-00915-0 (PMC7370495; doi:10.1186/s13045-020-00915-0)
Supplement: Supplementary file 1 — Additional file 1. GBD overview. [file 13045_2020_915_MOESM1_ESM.docx]

**GBD Overview**

The Global Burden of Disease (GBD) is an approach to global descriptive epidemiology. It is a systematic, scientific effort to quantify the comparative magnitude of health loss due to diseases, injuries, and risk factors by age, sex, and geographies for specific points in time. IHME serves as the coordinating center for the GBD and affiliated projects.

Incidence data is obtained from individual cancer registries or aggregated databases of cancer registries, such as CI5 (cancer incidence of five continents), SEER, EUERG or NORDCAN. GBD study relies on a lot of data – over 90,000 data sources. GBD produces regular estimates of all‐cause mortality, deaths by cause, years of life lost due to premature mortality (YLLs), years lived with disability (YLDs), and disability‐adjusted life years (DALYs) for a cause list. The cause list is agreed upon annually by the Scientific Council. The critical milestones for ongoing estimation include regular updates to the GBD estimates, referred to as the “GBD round.” For each round, the entire time series back to 1990 is re‐estimated using all available data to ensure the most complete and highly comparable set of estimates possible. Previous results will be archived every time new results are released. The GBD provides cutting‐edge and timely results through scientific papers, policy reports, web content, and interactive visualizations.

Published in November 2018, GBD 2017 provides for the first time an independent estimation of population, for each of 195 countries and territories and the globe, using a standardized, replicable approach, as well as a comprehensive update on fertility. GBD 2017 incorporates major data additions and improvements, and methodological refinements. Mortality and life expectancy estimates have been extended back to 1950, and new causes have been added to the fatal and non-fatal cause lists, for a total of 359 diseases and injuries (<http://www.healthdata.org/gbd/about/protocol>).

GBD 2017 incorporated a large number and wide variety of input sources to estimate mortality, causes of death and illness, and risk factors for 195 countries and territories from 1990-2017. These input sources are accessible through an interactive citation tool available in IHME’s GHDx. Users can retrieve citations for a specific GBD component, cause or risk, and location by choosing from the available selection boxes. They can then view and access GHDx records for input sources and export a CSV file that includes the GHDx metadata, citations, and information about where the data were used in GBD.

**Definition of indicator**

The GBD cause list is organized in a hierarchy. Levels 1 and 2 represent general groupings. The broad group “neoplasms.” which includes all malignant and benign neoplasms, is at Level 2 under the Level 1 group “Non-communicable diseases.” Level 3 includes 29 cancer groups, and Level 4 includes 37 groups since in Level 4, leukemia, liver cancer, and non-melanoma skin cancer are further subdivided. In this publication, estimates for the GBD cancer groups, for both sexes, for the time from 1980 to 2017, and for the 5-year GBD age groups (0-5; 5-9; etc. until 95+) are presented for 195 countries or territories.

**Data analysis**

EAPC, which is approximately equal to the annual change for a specified range, was calculated using the following regression model to assess the trends in ASR: Y=α+βX+ε, where y refers to ln(ASR), x represents calendar year, ε means error term, and β determines the positive or negative trends in ASR. The EAPC could be given by 100*(exp(β)-1), as well as its 95% confidence interval (CI).

Disability adjusted life-years were also defined as years of healthy life lost, estimated by the sum of the YLLs and the YLDs. YLLs, the years of life lost due to premature mortality, are the multiplication of deaths and a standard life expectancy at each age of death. In each population greater than 5 million, the standard life expectancy was estimated from a life table which includes the lowest observed mortality at each age group. YLDs were defined as the years lived with any short-term or long-term health loss weighted for severity by the disability weights, calculated by multiplying prevalence estimate and the disability weight of each mutually exclusive sequela for prostate cancer, such as disability due to incontinence and impotence from prostatectomy.

**GBD world population age standard**

Age-standardized populations in the GBD were calculated using the GBD world population age standard. For GBD 2017, the non-weighted mean of 2017 age-specific proportional distributions from the GBD 2017 population estimates for all national locations with a population greater than 5 million people in 2017 were used for generating an updated standard population age structure.

**Data sources**

**Cancer incidence data sources**

Data on cancer incidence were sought from individual population-based cancer registries as well as from databases that include multiple registries, including Cancer Incidence in Five Continents, NORDCAN, and EUREG. Cancer registries were identified through the membership list of the International Association of Cancer Registries, through the GBD collaborator network, or through the GHDx. Data were excluded if they were not representative of the coverage population (e.g., hospital-based registries), if they did not cover all malignant neoplasms as defined in ICD9 (140-208) or ICD10 (C00-C96) (e.g., specialty cancer registry), if they did not include data for both sexes and all age groups, if the data were limited to years prior to 1980, or if the source did not provide details on the population covered. Preference was given to registries with national coverage over those with only local coverage, except those from countries where the GBD study provides subnational estimates. Additional metadata for each source are available in the online GBD citation tool, <http://ghdx.healthdata.org/gbd-2017>. International Classification of Diseases (ICD) codes of tracheal, bronchus, and lung cancer incidence data were ICD10 (C33, C34-C34.92, Z12.2, Z80.1-Z80.2, Z85.1-Z85.20) and ICD9 (162-162.9, 209.21, V10.1-V10.20, V16.1-V16.2, V16.4-V16.40).

**Cancer mortality data sources**

A detailed description of the data sources and processing steps for the cause of death database can be found in the appendix to the GBD 2017 paper “Global, regional, and national age-sex-specific mortality for 282 causes of death in 195 countries and territories, 1980–2017: a systematic analysis for the Global Burden of Disease Study 2017.”

The cause of death (COD) database contains multiple sources of cancer mortality data. These sources include vital registration, verbal autopsy, and cancer registry data. The cancer registry mortality estimates that are uploaded into the COD database stem from cancer registry incidence data that have been transformed to mortality estimates through the use of mortality-to-incidence ratios (MIR). International Classification of Diseases (ICD) codes of tracheal, bronchus, and lung cancer mortality data were ICD10 (C33-C34.9, D02.1-D02.3, D14.2-D14.3,

D38.1) and ICD9 (162-162.9, 212.2-212.3, 231.1-231.2, 235.7).

The other group producing country-level cancer mortality estimates is the International Agency for Research on Cancer (IARC) with their GLOBOCAN database. Significantly different methods between the GBD study and GLOBOCAN can lead to differences in results. Whereas estimates in GLOBOCAN are based on the assumption that there are “In theory, […] as many methods as countries,” the cancer estimation process for the GBD study follows a coherent, well-documented method for all cancers, which allows cross-validation of models as well as determination of uncertainty. Another major difference is the ability in the GBD study to adjust single-cause estimates to the all-cause mortality, which is being determined independently. This also allows us to adjust individual causes of death to the all-cause mortality envelope, which permits us to correct for the underdiagnosis of cancer in countries with inadequate diagnostic resources. Redistribution of a fraction of undefined causes of death to certain cancers is another methodological advantage the GBD study has over GLOBOCAN, and estimates for cancer mortality can therefore differ substantially in countries with a large proportion of undefined causes of deaths in their vital registration data or a large proportion of undefined cancer cases in their cancer registry data.

There are certain limitations to consider when interpreting the GBD cancer mortality estimates. First, even though every effort is made to include the most recently available data for each country, dataseeking resources are not limitless and new data cannot always be accessed as soon as they are made available. It is therefore possible that the GBD study does not include all available data sources for cancer incidence or cancer mortality. Second, different redistribution methods can potentially change the cancer estimates substantially if the data sources used for the estimated location contain a large number of undefined causes; however, neglecting to account for these undefined deaths would likely introduce an even greater bias in the disease estimates. Third, using mortality-to-incidence ratios to transform cancer registry incidence data to mortality estimates requires accurate MIR. For GBD 2017 we have made further changes to the MIR estimation, but the method remains sensitive to underdiagnosis of cancer cases or underascertainment of cancer deaths. However, given that the majority of data used for the cancer mortality estimation come from vital registration data and not cancer registry data, this is not a major limitation.

**Cancer registry data**

Cancer registry data were used from publicly available sources or provided by collaborators. This analysis used all data from GBD 2016 and added registry data from Russia, Iran, Ethiopia, Norway, as well as the newly released CI5 XI (Cancer Registry in Five Continents).

**Inclusion and exclusion criteria**

Only population-based cancer registries were included, and only those that included all cancers (no specialty registries), data for all age groups, and data for both sexes. Pathology-based cancer registries were included if they had a defined population. Hospital-based cancer registries were excluded. Cancer registry data were excluded from either the final incidence data input or the MI model input if a more detailed source (eg, providing more detailed age or diagnostic groups) was available for the same population. Preference was given to registries with national coverage over those with only local coverage, except those from countries where the GBD study provides subnational estimates. Data were excluded if the coverage population was unknown.

**Age-sex splitting**

For the analysis of causes of death, we mapped these different age intervals to the GBD standard set of age groups. The approach to undertake this mapping was the same as in the prior GBD studies (GBD 2016, GBD 2015, GBD 2013, and GBD 2010).the detailed description can be found in the appendix to the GBD 2017 paper “Global, regional, and national age-sex-specific mortality for 282 causes of death in 195 countries and territories, 1980–2017: a systematic analysis for the Global Burden of Disease Study 2017.”

**SDI definition**

The Socio-demographic Index (SDI) is a composite indicator of development status strongly correlated with health outcomes. In short, it is the geometric mean of 0 to 1 indices of total fertility rate under the age of 25 (TFU25), mean education for those aged 15 and older (EDU15+), and lag distributed income (LDI) per capita. The composite Socio-demographic Index is the geometric mean of these three indices for a given location-year. The cutoff values used to determine quintiles for analysis were then computed using country-level estimates of SDI for the year 2017, excluding countries with populations less than 1 million.

**Bias of categories of input data**

Bias of the input data included for the COD database is described elsewhere. Cancer registry data can be biased in multiple ways. A high proportion of ill-defined cancer cases in the registry data requires redistribution of these cases to other cancers, which introduces a potential for bias. Changes between coding systems can lead to artificial differences in disease estimates; however, we adjust for this bias by mapping the different coding systems to the GBD causes. Since many cancer registries are located in urban areas, the representativeness of the registry for the general population can also be problematic. The accuracy of mortality data reported in cancer registries usually depends on the quality of the vital registration system. If the vital registration system is incomplete or of poor quality, the mortality-to-incidence ratio can be biased to lower ratios.

**Input data**

To estimate the prevalence of each of these categories for all locations, by age, year, and sex, the prevalence of these neoplasms from hospital data was used as input for a prevalence model in DisMod-MR 2.1. These inputs included MarketScan claims data from the United States in the years 2000, 2010, and 2012, as well as hospital and outpatient data from other health systems worldwide. Each of these data sources were crosswalked to the 2012 MarketScan data.
