# Supplementary figures and images for: Epidemiological trends of tracheal, bronchus, and lung cancer at the global, regional, and national levels: a population-based study
Source: J Hematol Oncol. 2020 Jul 20;13:98. doi: 10.1186/s13045-020-00915-0 (PMC7370495; doi:10.1186/s13045-020-00915-0)

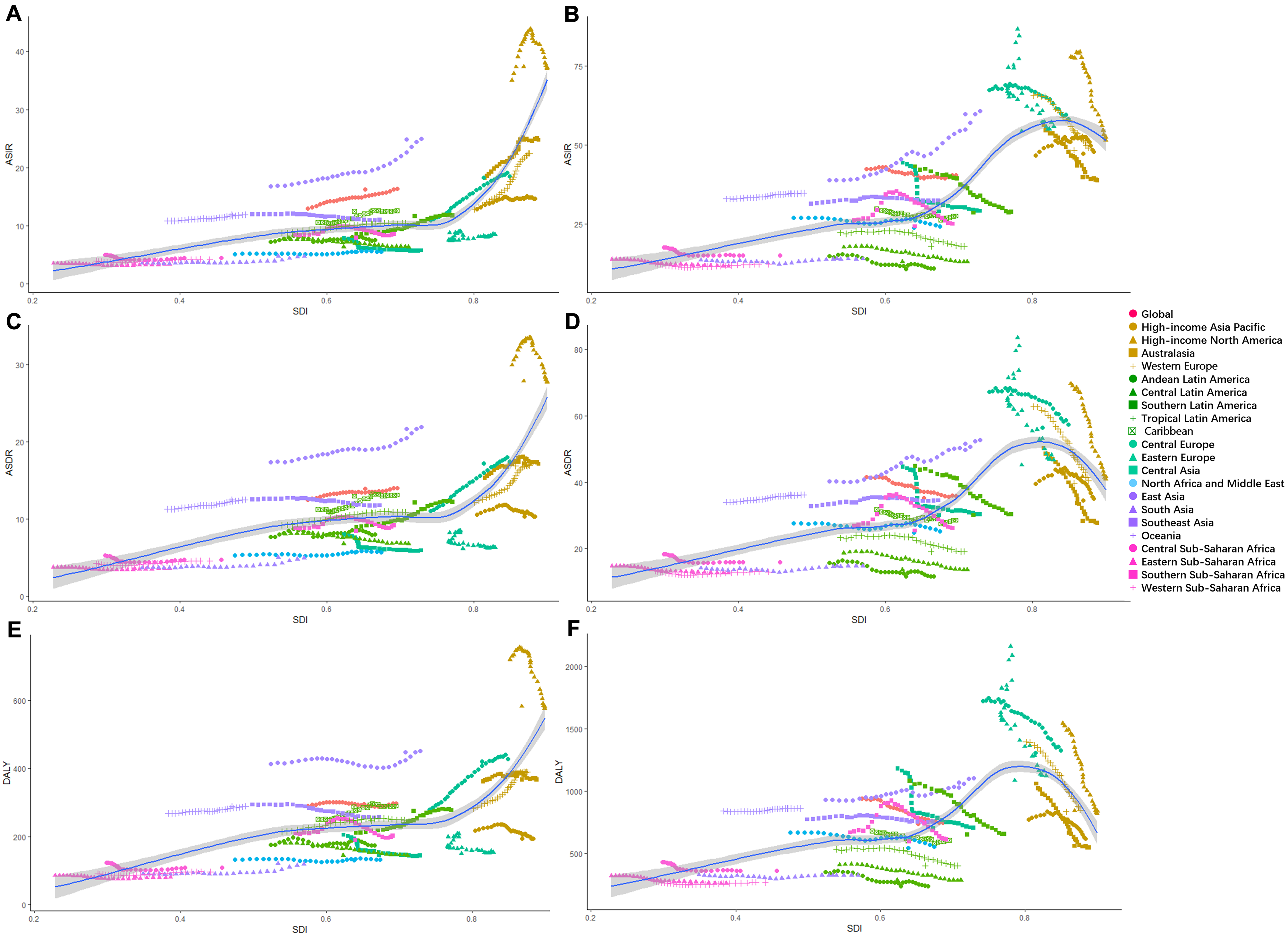

Supplement: Supplementary file 2 — Additional file 2: Figure S1. Age-standardized rates (per 100,000) of TBL cancer among regions based on SDI in 2017. Figure legends: (a) ASIR among females, (b) ASIR among males, (c) ASDR among females, (d) ASDR among males, (e) Age-standardized DALY rate among females, (f) Age-standardized DALY rate among males. ASIR, age-standardized incidence rate; ASDR, age-standardized death rate; DALY: disability adjusted life-year; SDI, socio-demographic index. [file 13045_2020_915_MOESM2_ESM.tif]

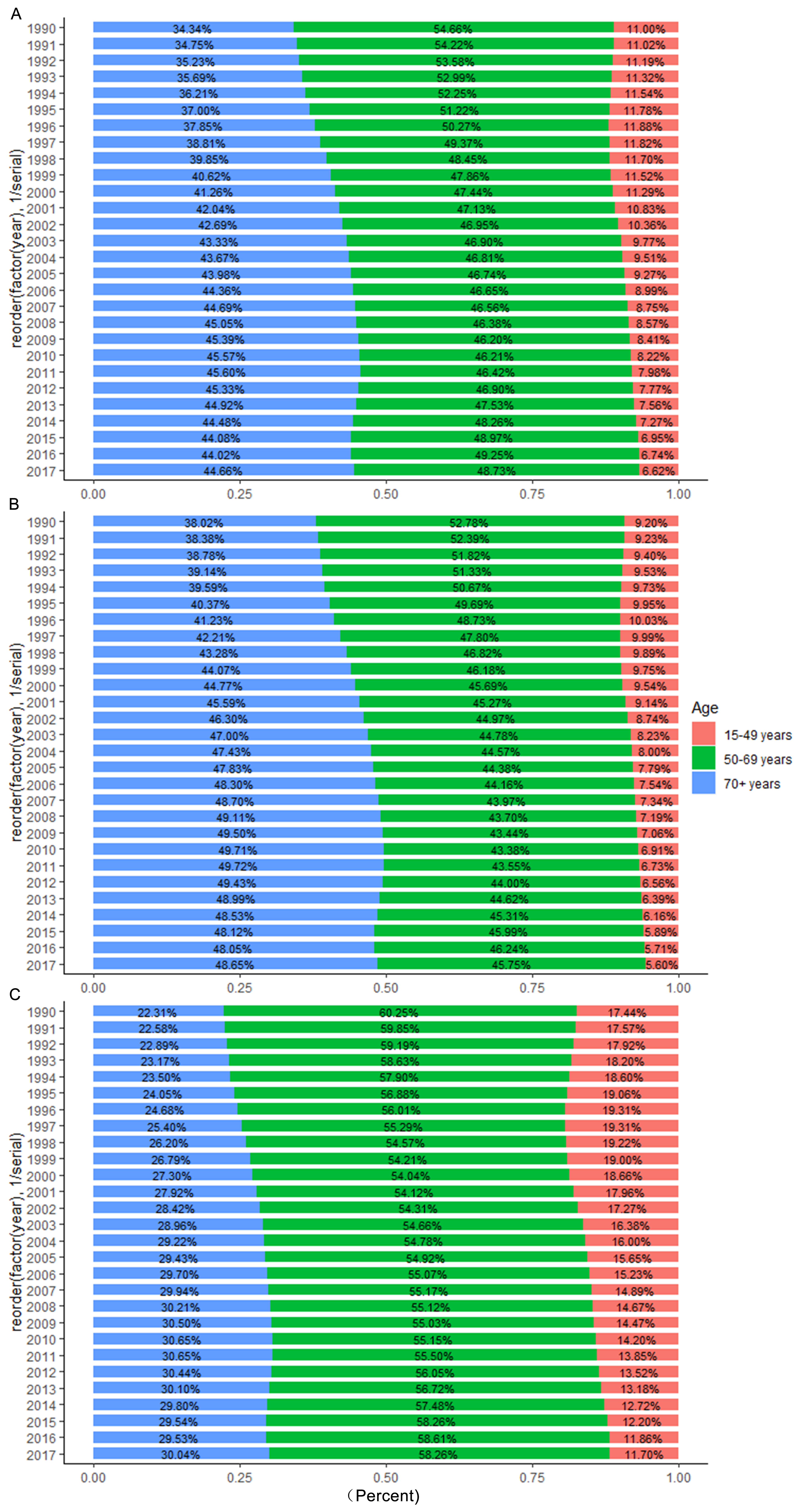

Supplement: Supplementary file 3 — Additional file 3: Figure S2. The proportion of different age subgroups in TBL cancer burden by years. Figure legends: (a) incident cases, (b) deaths and (c) DALYs. DALY: disability adjusted life-year. [file 13045_2020_915_MOESM3_ESM.tif]

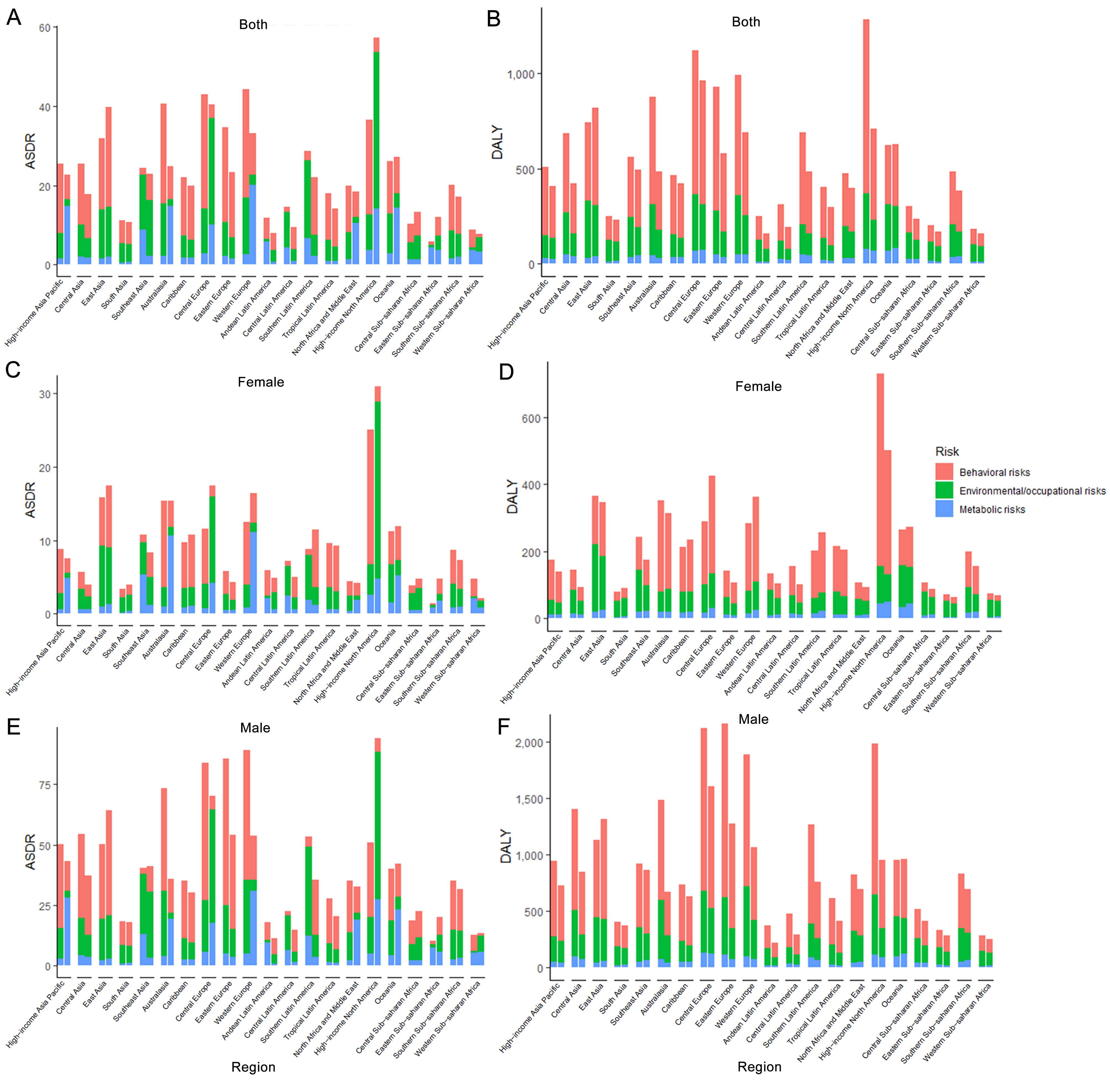

Supplement: Supplementary file 7 — Additional file 7: Figure S3. The ASDR and DALY of TBL cancer among different regions, genders and risk factors. Figure legends: The left column in each group is data in 1990 and the right column in 2017. (a) ASDR among both sexes; (b) Age standardized DALY rate among both sexes; (c) ASDR among female; (d) Age standardized DALY rate among female; (e) ASDR among male; (f) Age standardized DALY rate among male. ASDR, Age standardized death rate; DALY: disability adjusted life-year. [file 13045_2020_915_MOESM7_ESM.tif]

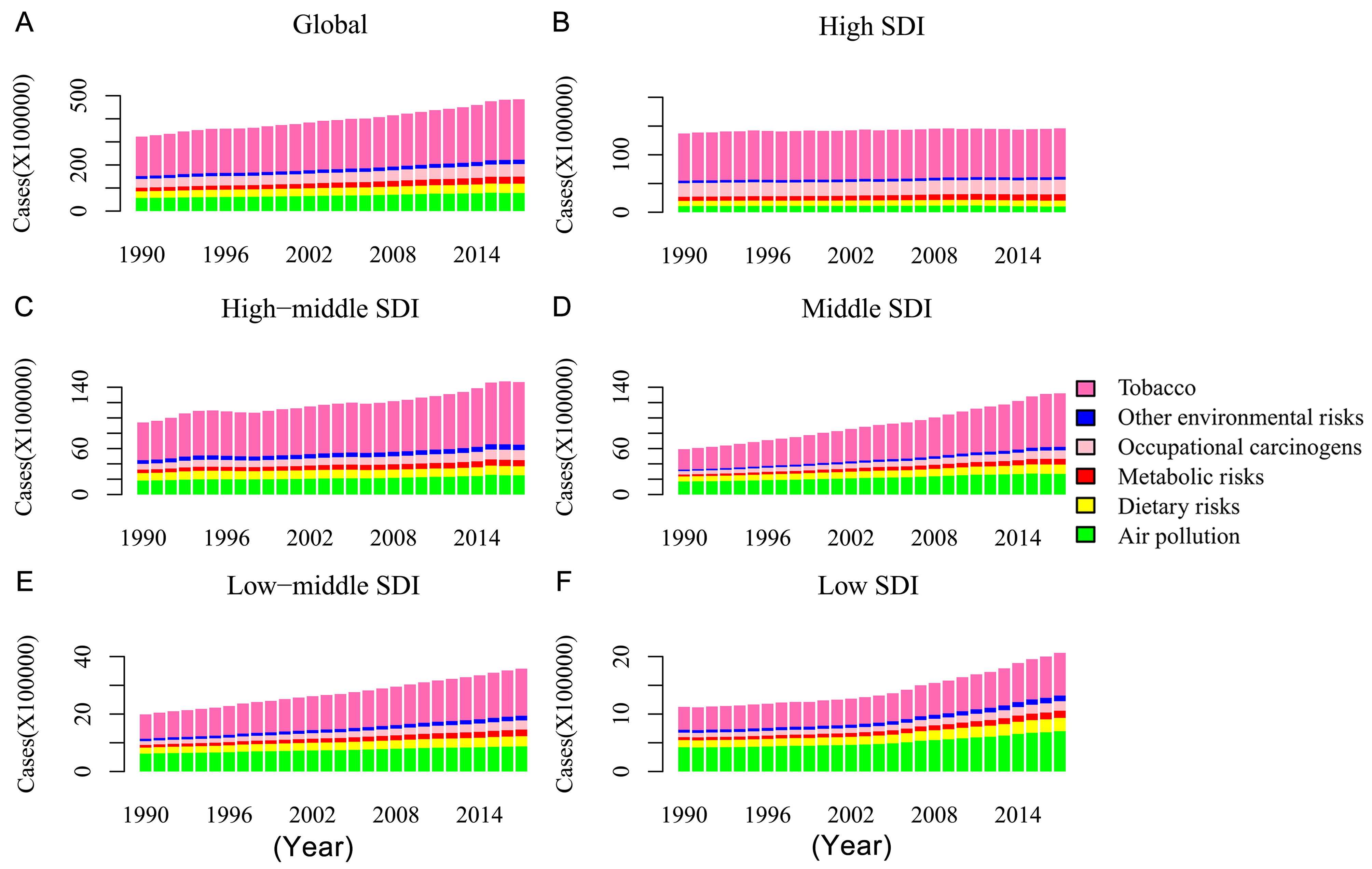

Supplement: Supplementary file 8 — Additional file 8: Figure S4. The change trends of TBL cancer deaths among SDI quintiles and risks over 28 years. Figure legends: SDI, socio-demographic index. [file 13045_2020_915_MOESM8_ESM.tif]
