# Supplementary material for: Epidemiological trends of tracheal, bronchus, and lung cancer at the global, regional, and national levels: a population-based study
Source: J Hematol Oncol. 2020 Jul 20;13:98. doi: 10.1186/s13045-020-00915-0 (PMC7370495; doi:10.1186/s13045-020-00915-0)
Supplement: Supplementary file 4 — Additional file 4: Table S1. Three countries/regions with top and bottom burden of tracheal, bronchus, and lung cancer. [file 13045_2020_915_MOESM4_ESM.docx]

**Table S1. Three countries/regions with top and bottom burden of tracheal, bronchus, and lung cancer.**

| **Period** | **Measure** | **Top three countries/regions** | | | | | | **Bottom three countries/regions** | | | | | |
| --- | --- | --- | --- | --- | --- | --- | --- | --- | --- | --- | --- | --- | --- |
|  |  | **Both** | **Number** | **Female** | **Number** | **Male** | **Number** | **Both** | **Number** | **Female** | **Number** | **Male** | **Number** |
| **Country** | | | | | | | | | | | | | |
| 1990 | incidence | China | 241036.12 | China | 75646.65 | China | 165389.47 | Antigua | 4.22 | Antigua | 1.11 | Marshall Islands | 3.50 |
|  |  | USA | 174213.44 | USA | 64556.89 | USA | 109656.55 | Barbuda | 4.22 | Barbuda | 1.11 | Antigua | 3.11 |
|  |  | Russia | 63832.81 | UK | 14442.73 | Russia | 53570.20 | Marshall Islands | 4.17 | Marshall Islands | 0.66 | Barbuda | 3.11 |
|  | death | China | 240475.21 | China | 76027.55 | China | 164447.66 | Antigua | 4.48 | Barbuda | 1.18 | Marshall Islands | 3.51 |
|  |  | USA | 152628.98 | USA | 55583.60 | USA | 97045.38 | Barbuda | 4.48 | Northern Mariana Islands | 1.17 | Antigua | 3.30 |
|  |  | Russia | 60306.56 | UK | 13569.99 | Russia | 50451.92 | Marshall Islands | 4.19 | Marshall Islands | 0.67 | Barbuda | 3.30 |
|  | DALY | China | 6348558.58 | China | 1975846.89 | China | 4372711.69 | Marshall Islands | 111.77 | Antigua | 26.10 | Marshall Islands | 94.12 |
|  |  | USA | 3357006.35 | USA | 1212145.37 | USA | 2144860.98 | Antigua | 98.97 | Barbuda | 26.10 | Antigua | 72.86 |
|  |  | Russia | 1609315.65 | UK | 270347.47 | Russia | 1384862.76 | Barbuda | 98.97 | Marshall Islands | 17.65 | Barbuda | 72.86 |
|  | ASIR (per 100000 persons) | Greenland | 105.92 | Greenland | 80.14 | Greenland | 141.53 | Malawi | 5.84 | India | 2.84 | Malawi | 8.71 |
|  |  | USA | 54.05 | USA | 35.81 | Czech Republic | 92.18 | Egypt | 5.74 | Tunisia | 2.78 | Egypt | 8.38 |
|  |  | UK | 46.02 | Denmark | 30.76 | Belgium | 90.93 | Uganda | 5.31 | Saudi Arabia | 2.25 | Uganda | 6.89 |
|  | ASDR (per 100000 persons) | Greenland | 112.91 | Greenland | 84.59 | Greenland | 153.97 | Malawi | 6.34 | Egypt | 2.94 | El Salvador | 9.26 |
|  |  | USA | 46.95 | USA | 30.30 | Belgium | 93.87 | Egypt | 5.92 | Tunisia | 2.83 | Egypt | 8.71 |
|  |  | Hungary | 45.90 | Brunei | 27.83 | Czech Republic | 92.49 | Uganda | 5.75 | Saudi Arabia | 2.38 | Uganda | 7.53 |
|  | Age standardized DALY rate (per 100000 persons) | Greenland | 2468.23 | Greenland | 1910.86 | Greenland | 3138.13 | Kenya | 140.92 | India | 69.89 | Guatemala | 205.45 |
|  |  | Hungary | 1202.93 | USA | 725.64 | Czech Republic | 2257.11 | Malawi | 132.55 | Tunisia | 61.39 | Malawi | 195.55 |
|  |  | Czech Republic | 1157.02 | Denmark | 697.75 | Hungary | 2172.96 | Uganda | 124.08 | Saudi Arabia | 54.34 | Uganda | 156.68 |
| 2017 | incidence | China | 813209.20 | China | 249117.19 | China | 564092.02 | Marshall Islands | 8.33 | Samoa | 2.90 | Marshall Islands | 6.74 |
|  |  | USA | 247661.38 | USA | 112879.67 | USA | 134781.71 | Antigua | 7.61 | American Samoa | 2.85 | Antigua | 4.64 |
|  |  | Japan | 107264.93 | Japan | 32002.72 | Japan | 75262.21 | Barbuda | 7.61 | Marshall Islands | 1.59 | Barbuda | 4.64 |
|  | death | China | 692388.62 | China | 215142.84 | China | 477245.77 | Marshall Islands | 8.27 | Samoa | 3.02 | Kiribati | 6.65 |
|  |  | USA | 190697.62 | USA | 84931.54 | USA | 105766.07 | Antigua | 7.93 | American Samoa | 2.91 | Antigua | 4.82 |
|  |  | India | 83891.46 | India | 24114.98 | India | 59776.48 | Barbuda | 7.93 | Marshall Islands | 1.56 | Barbuda | 4.82 |
|  | DALY | China | 15252918.02 | China | 4581367.73 | China | 10671550.29 | Marshall Islands | 231.32 | Antigua | 69.51 | Samoa | 170.16 |
|  |  | USA | 3765845.00 | USA | 1639848.80 | USA | 2125996.20 | Antigua | 178.67 | Barbuda | 69.51 | Antigua | 109.15 |
|  |  | India | 2059937.63 | India | 608750.78 | India | 1451186.85 | Barbuda | 178.67 | Marshall Islands | 45.08 | Barbuda | 109.15 |
|  | ASIR (per 100000 persons) | Greenland | 71.02 | Greenland | 54.80 | Greenland | 86.90 | Kenya | 5.92 | Kenya | 2.69 | Malawi | 8.93 |
|  |  | Hungary | 45.39 | Denmark | 40.97 | Montenegro | 69.81 | Nicaragua | 5.86 | Malawi | 2.65 | Guatemala | 8.78 |
|  |  | USA | 44.99 | USA | 37.80 | Hungary | 67.88 | Malawi | 5.29 | Maldives | 2.59 | Nicaragua | 8.05 |
|  | ASDR (per 100000 persons) | Greenland | 76.18 | Greenland | 58.23 | Greenland | 94.30 | Kenya | 6.43 | Kenya | 2.89 | Oman | 9.66 |
|  |  | Hungary | 45.30 | Denmark | 31.54 | Montenegro | 71.60 | Nicaragua | 6.26 | Malawi | 2.86 | Guatemala | 9.49 |
|  |  | Montenegro | 43.79 | Hungary | 28.53 | Hungary | 68.99 | Malawi | 5.71 | Maldives | 2.72 | Nicaragua | 8.62 |
|  | Age standardized DALY rate (per 100000 persons) | Greenland | 1584.94 | Greenland | 1244.05 | Greenland | 1892.33 | Kenya | 134.10 | Malawi | 62.32 | Guatemala | 195.62 |
|  |  | Hungary | 1115.57 | Hungary | 718.98 | Montenegro | 1658.49 | Nicaragua | 132.06 | Kenya | 61.96 | Oman | 193.15 |
|  |  | Montenegro | 1048.64 | Denmark | 665.63 | Hungary | 1631.33 | Malawi | 121.91 | Maldives | 59.29 | Nicaragua | 178.72 |
| 1990-2017 | incidence | United Arab Emirates | 783.54 | United Arab Emirates | 632.79 | United Arab Emirates | 815.40 | Ukraine | -23.38 | Kyrgyzstan | -11.64 | Estonia | -24.99 |
|  |  | Qatar | 419.91 | Qatar | 468.11 | Qatar | 412.26 | Kyrgyzstan | -33.49 | Ukraine | -23.59 | Kazakhstan | -37.16 |
|  |  | South Korea | 309.43 | Jordan | 402.86 | South Korea | 289.92 | Kazakhstan | -35.55 | Kazakhstan | -27.92 | Kyrgyzstan | -38.37 |
|  | death | United Arab Emirates | 743.79 | United Arab Emirates | 604.34 | United Arab Emirates | 772.76 | Kyrgyzstan | -32.53 | Belarus | -15.87 | Ukraine | -33.76 |
|  |  | Qatar | 385.84 | Qatar | 416.00 | Qatar | 381.07 | Kazakhstan | -34.21 | Kazakhstan | -27.55 | Kazakhstan | -35.66 |
|  |  | Jordan | 280.57 | Jordan | 412.35 | Djibouti | 286.01 | Ukraine | -34.40 | Ukraine | -37.37 | Kyrgyzstan | -37.25 |
|  | DALY | United Arab Emirates | 824.26 | United Arab Emirates | 659.14 | United Arab Emirates | 859.95 | Kyrgyzstan | -35.62 | Belarus | -21.48 | Estonia | -39.19 |
|  |  | Qatar | 387.36 | Qatar | 427.15 | Qatar | 380.75 | Ukraine | -37.86 | Kazakhstan | -30.76 | Kyrgyzstan | -40.84 |
|  |  | Belize | 270.71 | Jordan | 360.55 | Djibouti | 255.75 | Kazakhstan | -40.06 | Ukraine | -38.19 | Kazakhstan | -41.85 |
|  | ASIR (per 100000 persons) | Cyprus | 1.99 | Spain | 4.32 | Cyprus | 1.97 | Maldives | -2.79 | Mongolia | -2.61 | Kazakhstan | -2.99 |
|  |  | Georgia | 1.69 | France | 4.15 | China | 1.80 | Kazakhstan | -2.84 | Thailand | -2.74 | Iraq | -3.02 |
|  |  | China | 1.69 | Netherlands | 3.99 | Georgia | 1.78 | Bahrain | -4.76 | Bahrain | -3.56 | Bahrain | -5.07 |
|  | ASDR (per 100000 persons) | Georgia | 1.78 | France | 3.28 | Georgia | 1.85 | Kazakhstan | -2.73 | Singapore | -2.49 | Kazakhstan | -2.89 |
|  |  | Chad | 1.41 | Netherlands | 2.98 | Paraguay | 1.52 | Maldives | -2.78 | Thailand | -2.66 | Iraq | -3.00 |
|  |  | El Salvador | 1.27 | Spain | 2.89 | Lesotho | 1.35 | Bahrain | -4.78 | Bahrain | -3.58 | Bahrain | -5.06 |
|  | Age standardized DALY rate (per 100000 persons) | Georgia | 1.55 | France | 3.56 | Georgia | 1.67 | Maldives | -3.07 | Mongolia | -2.94 | Kazakhstan | -3.32 |
|  |  | Chad | 1.30 | Spain | 3.41 | Lesotho | 1.51 | Kazakhstan | -3.20 | Thailand | -3.09 | Czech Republic | -3.39 |
|  |  | Lesotho | 1.27 | Serbia | 2.51 | Paraguay | 1.28 | Bahrain | -4.91 | Bahrain | -3.74 | Bahrain | -5.29 |
|  |  |  |  |  |  |  |  |  |  |  |  |  |  |
| **Region** | | | | | | | | | | | | | |
| 1990 | incidence | East Asia | 252921.22 | East Asia | 79314.04 | East Asia | 173607.19 | Central Sub-Saharan Africa | 2515.60 | Andean Latin America | 797.65 | Central Sub-Saharan Africa | 1907.57 |
|  |  | Western Europe | 206079.30 | High-income North America | 69516.27 | Western Europe | 163113.29 | Andean Latin America | 2276.07 | Central Sub-Saharan Africa | 608.04 | Andean Latin America | 1478.42 |
|  |  | High-income North America | 189379.41 | Western Europe | 42966.01 | High-income North America | 119863.14 | Oceania | 678.57 | Oceania | 155.24 | Oceania | 523.33 |
|  | death | East Asia | 252235.26 | East Asia | 79680.93 | East Asia | 172554.33 | Central Sub-Saharan Africa | 2538.04 | Andean Latin America | 817.12 | Central Sub-Saharan Africa | 1932.49 |
|  |  | Western Europe | 197700.34 | High-income North America | 60387.54 | Western Europe | 155898.86 | Andean Latin America | 2358.24 | Central Sub-Saharan Africa | 605.56 | Andean Latin America | 1541.12 |
|  |  | High-income North America | 167419.29 | Western Europe | 41801.48 | High-income North America | 107031.75 | Oceania | 660.12 | Oceania | 151.34 | Oceania | 508.77 |
|  | DALY | East Asia | 6655766.02 | East Asia | 2070825.13 | East Asia | 4584940.89 | Central Sub-Saharan Africa | 68354.97 | Andean Latin America | 20585.35 | Central Sub-Saharan Africa | 51466.14 |
|  |  | Western Europe | 4339975.86 | High-income North America | 1322095.12 | Western Europe | 3474770.19 | Andean Latin America | 57998.74 | Central Sub-Saharan Africa | 16888.83 | Andean Latin America | 37413.39 |
|  |  | High-income North America | 3685790.22 | Western Europe | 865205.67 | High-income North America | 2363695.10 | Oceania | 19513.81 | Oceania | 4352.47 | Oceania | 15161.33 |
|  | ASIR (per 100000 persons) | High-income North America | 53.24 | High-income North America | 35.06 | High-income North America | 77.92 | South Asia | 8.69 | Western Sub-Saharan Africa | 3.95 | Eastern Sub-Saharan Africa | 13.72 |
|  |  | Central Europe | 35.90 | Australasia | 18.17 | Eastern Europe | 74.71 | Eastern Sub-Saharan Africa | 8.64 | South Asia | 3.56 | South Asia | 13.45 |
|  |  | Western Europe | 35.36 | East Asia | 16.82 | Central Europe | 67.42 | Western Sub-Saharan Africa | 8.21 | Eastern Sub-Saharan Africa | 3.56 | Western Sub-Saharan Africa | 12.45 |
|  | ASDR (per 100000 persons) | High-income North America | 46.67 | High-income North America | 29.93 | Eastern Europe | 71.50 | Eastern Sub-Saharan Africa | 9.30 | Western Sub-Saharan Africa | 4.23 | Eastern Sub-Saharan Africa | 14.82 |
|  |  | Central Europe | 35.56 | East Asia | 17.38 | High-income North America | 69.71 | South Asia | 9.12 | Eastern Sub-Saharan Africa | 3.79 | South Asia | 14.19 |
|  |  | Western Europe | 33.43 | Australasia | 15.35 | Central Europe | 67.20 | Western Sub-Saharan Africa | 8.80 | South Asia | 3.70 | Western Sub-Saharan Africa | 13.42 |
|  | Age standardized DALY rate (per 100000 persons) | High-income North America | 1083.68 | High-income North America | 717.60 | Eastern Europe | 1823.86 | South Asia | 213.17 | Western Sub-Saharan Africa | 91.28 | South Asia | 325.99 |
|  |  | Central Europe | 933.31 | East Asia | 413.68 | Central Europe | 1720.36 | Eastern Sub-Saharan Africa | 201.39 | South Asia | 89.80 | Eastern Sub-Saharan Africa | 316.99 |
|  |  | Eastern Europe | 838.20 | Australasia | 361.36 | High-income North America | 1542.24 | Western Sub-Saharan Africa | 185.79 | Eastern Sub-Saharan Africa | 85.71 | Western Sub-Saharan Africa | 275.69 |
| 2017 | incidence | East Asia | 845753.16 | East Asia | 259692.75 | East Asia | 586060.41 | Andean Latin America | 4858.80 | Andean Latin America | 2105.73 | Central Sub-Saharan Africa | 3310.27 |
|  |  | Western Europe | 292856.05 | High-income North America | 124709.67 | Western Europe | 191502.48 | Central Sub-Saharan Africa | 4525.21 | Central Sub-Saharan Africa | 1214.94 | Andean Latin America | 2753.08 |
|  |  | High-income North America | 273198.23 | Western Europe | 101353.56 | High-income North America | 148488.55 | Oceania | 1549.08 | Oceania | 370.47 | Oceania | 1178.62 |
|  | death | East Asia | 722055.95 | East Asia | 224689.82 | East Asia | 497366.13 | Andean Latin America | 5198.13 | Andean Latin America | 2234.58 | Central Sub-Saharan Africa | 3342.30 |
|  |  | Western Europe | 241322.52 | High-income North America | 94640.89 | Western Europe | 160514.31 | Central Sub-Saharan Africa | 4572.61 | Central Sub-Saharan Africa | 1230.31 | Andean Latin America | 2963.55 |
|  |  | High-income North America | 212190.98 | Western Europe | 80808.21 | High-income North America | 117550.08 | Oceania | 1516.43 | Oceania | 363.62 | Oceania | 1152.81 |
|  | DALY | East Asia | 15905049.23 | East Asia | 4783265.08 | East Asia | 11121784.16 | Central Sub-Saharan Africa | 123988.56 | Andean Latin America | 49230.20 | Central Sub-Saharan Africa | 90907.40 |
|  |  | Western Europe | 4725697.21 | High-income North America | 1830613.44 | Western Europe | 3145844.94 | Andean Latin America | 111232.45 | Central Sub-Saharan Africa | 33081.16 | Andean Latin America | 62002.24 |
|  |  | High-income North America | 4183222.64 | Western Europe | 1579852.27 | High-income North America | 2352609.20 | Oceania | 44101.60 | Oceania | 10329.48 | Oceania | 33772.12 |
|  | ASIR (per 100000 persons) | High-income North America | 44.22 | High-income North America | 37.33 | East Asia | 59.79 | Andean Latin America | 9.07 | Central Sub-Saharan Africa | 4.51 | Western Sub-Saharan Africa | 12.12 |
|  |  | East Asia | 41.54 | East Asia | 24.84 | Central Europe | 57.30 | Western Sub-Saharan Africa | 8.06 | Western Sub-Saharan Africa | 4.26 | Eastern Sub-Saharan Africa | 11.59 |
|  |  | Central Europe | 35.42 | Australasia | 24.81 | Eastern Europe | 54.37 | Eastern Sub-Saharan Africa | 7.21 | Eastern Sub-Saharan Africa | 3.37 | Andean Latin America | 10.73 |
|  | ASDR (per 100000 persons) | East Asia | 35.94 | High-income North America | 27.84 | Central Europe | 56.46 | Central Sub-Saharan Africa | 9.63 | Central Sub-Saharan Africa | 4.78 | Western Sub-Saharan Africa | 13.10 |
|  |  | Central Europe | 34.30 | East Asia | 21.71 | East Asia | 51.60 | Western Sub-Saharan Africa | 8.71 | Western Sub-Saharan Africa | 4.58 | Eastern Sub-Saharan Africa | 12.72 |
|  |  | High-income North America | 34.09 | Central Europe | 17.20 | Eastern Europe | 45.20 | Eastern Sub-Saharan Africa | 7.83 | Eastern Sub-Saharan Africa | 3.60 | Andean Latin America | 11.66 |
|  | Age standardized DALY rate (per 100000 persons) | Central Europe | 822.54 | High-income North America | 581.70 | Central Europe | 1304.98 | Andean Latin America | 204.07 | Central Sub-Saharan Africa | 109.57 | Western Sub-Saharan Africa | 264.59 |
|  |  | East Asia | 751.11 | East Asia | 447.20 | Eastern Europe | 1082.79 | Western Sub-Saharan Africa | 178.33 | Western Sub-Saharan Africa | 97.13 | Eastern Sub-Saharan Africa | 258.11 |
|  |  | High-income North America | 702.16 | Central Europe | 420.52 | East Asia | 1068.89 | Eastern Sub-Saharan Africa | 164.80 | Eastern Sub-Saharan Africa | 80.35 | Andean Latin America | 235.78 |
| 1990-2017 | incidence | East Asia | 234.39 | East Asia | 227.42 | East Asia | 237.58 | Central Europe | 33.85 | High-income North America | 79.40 | Southern Latin America | 11.43 |
|  |  | High-income Asia Pacific | 146.61 | South Asia | 219.48 | High-income Asia Pacific | 138.88 | Central Asia | 1.01 | Central Asia | 8.68 | Central Asia | -0.81 |
|  |  | South Asia | 136.30 | Tropical Latin America | 201.77 | Oceania | 125.21 | Eastern Europe | -8.87 | Eastern Europe | 6.66 | Eastern Europe | -11.92 |
|  | death | East Asia | 186.26 | South Asia | 227.67 | East Asia | 188.24 | Western Europe | 22.06 | High-income North America | 56.72 | Western Europe | 2.96 |
|  |  | South Asia | 142.52 | Tropical Latin America | 209.08 | Oceania | 126.59 | Central Asia | 2.95 | Central Asia | 8.35 | Central Asia | 1.63 |
|  |  | Tropical Latin America | 131.49 | East Asia | 181.97 | Southeast Asia | 124.65 | Eastern Europe | -21.10 | Eastern Europe | -13.37 | Eastern Europe | -22.64 |
|  | DALY | East Asia | 138.97 | South Asia | 203.95 | East Asia | 142.57 | Western Europe | 8.89 | High-income North America | 38.46 | Central Asia | -6.82 |
|  |  | Oceania | 126.00 | Tropical Latin America | 177.83 | Oceania | 122.75 | Central Asia | -4.07 | Central Asia | 8.32 | Western Europe | -9.47 |
|  |  | South Asia | 123.23 | Andean Latin America | 139.15 | Southeast Asia | 106.19 | Eastern Europe | -27.98 | Eastern Europe | -16.98 | Eastern Europe | -29.81 |
|  | ASIR (per 100000 persons) | East Asia | 1.66 | Western Europe | 2.27 | East Asia | 1.77 | Eastern Europe | -1.23 | Central Sub-Saharan Africa | -0.53 | Central Asia | -1.62 |
|  |  | Oceania | 0.26 | Central Europe | 2.24 | Oceania | 0.23 | Central Latin America | -1.25 | Central Latin America | -0.82 | Southern Latin America | -1.66 |
|  |  | High-income Asia Pacific | 0.25 | East Asia | 1.42 | South Asia | 0.20 | Central Asia | -1.50 | Central Asia | -1.13 | High-income North America | -1.82 |
|  | ASDR (per 100000 persons) | East Asia | 1.02 | Central Europe | 1.94 | East Asia | 1.10 | Central Asia | -1.36 | Central Latin America | -0.81 | Eastern Europe | -2.08 |
|  |  | Oceania | 0.30 | Western Europe | 1.39 | Oceania | 0.27 | High-income North America | -1.44 | Central Asia | -1.08 | High-income North America | -2.21 |
|  |  | South Asia | 0.25 | Southern Latin America | 1.35 | South Asia | 0.26 | Eastern Europe | -1.88 | Eastern Europe | -1.20 | Australasia | -2.38 |
|  | Age standardized DALY rate (per 100000 persons) | East Asia | 0.42 | Central Europe | 1.81 | Western Sub-Saharan Africa | 0.58 | Central Asia | -1.87 | High-income North America | -1.06 | Oceania | -2.37 |
|  |  | Oceania | 0.19 | Western Europe | 1.35 | Southern Sub-Saharan Africa | 0.15 | High-income North America | -1.92 | Eastern Europe | -1.20 | North Africa and Middle East | -2.60 |
|  |  | South Asia | 0.10 | Southern Latin America | 1.23 | Central Sub-Saharan Africa | 0.05 | Eastern Europe | -2.18 | Central Asia | -1.31 | Central Europe | -2.64 |

Abbreviations: ASDR, age standardized death rate; ASIR, age standardized incidence rate; DALY, disability adjusted life-year; EAPC: estimated annual percentage change; CI: confidence interval; UI: uncertainty interval.
