# Supplementary material for: Epidemiological trends of tracheal, bronchus, and lung cancer at the global, regional, and national levels: a population-based study
Source: J Hematol Oncol. 2020 Jul 20;13:98. doi: 10.1186/s13045-020-00915-0 (PMC7370495; doi:10.1186/s13045-020-00915-0)
Supplement: Supplementary file 5 — Additional file 5: Table S2. The incidence, deaths, DALYs and variations of TBL cancer from 1990 to 2017 among countries. [file 13045_2020_915_MOESM5_ESM.docx]

**Table S2. The incidence, death, DALYs and variations of TBL cancer from 1990 to 2017 among countries.**

| **Measure** | **Location** | **Sex** | **1990 (No.)** | | | | **2017(No.)** | | | | **1990-2017 Increase Percent (%)** |
| --- | --- | --- | --- | --- | --- | --- | --- | --- | --- | --- | --- |
|  |  |  | **Both (No.)** | **Male (No.)** | **Female (No.)** | **Male/Female Ratio** | **Both (No.)** | **Male (No.)** | **Female (No.)** | **Male/Female ratio** |  |
| DALYs | Afghanistan | Both | 23837.07 | 18993.24 | 4843.83 | 3.92 | 34066.93 | 24331.16 | 9735.77 | 2.50 | 42.92 |
| DALYs | Albania | Both | 13884.05 | 11715.32 | 2168.73 | 5.40 | 22649.89 | 18833.55 | 3816.34 | 4.93 | 63.14 |
| DALYs | Algeria | Both | 32031.90 | 26696.88 | 5335.02 | 5.00 | 66027.25 | 52670.76 | 13356.49 | 3.94 | 106.13 |
| DALYs | American Samoa | Both | 151.24 | 119.56 | 31.69 | 3.77 | 253.49 | 180.48 | 73.01 | 2.47 | 67.61 |
| DALYs | Andorra | Both | 434.95 | 340.56 | 94.39 | 3.61 | 702.56 | 480.07 | 222.48 | 2.16 | 61.52 |
| DALYs | Angola | Both | 15745.87 | 12606.37 | 3139.50 | 4.02 | 31203.38 | 23208.92 | 7994.46 | 2.90 | 98.17 |
| DALYs | Antigua | Both | 98.97 | 72.86 | 26.10 | 2.79 | 178.67 | 109.15 | 69.51 | 1.57 | 80.53 |
| DALYs | Argentina | Both | 215588.37 | 177160.88 | 38427.49 | 4.61 | 250099.10 | 169880.77 | 80218.33 | 2.12 | 16.01 |
| DALYs | Armenia | Both | 24614.48 | 20850.39 | 3764.08 | 5.54 | 28698.85 | 24088.75 | 4610.10 | 5.23 | 16.59 |
| DALYs | Australia | Both | 134147.39 | 99013.64 | 35133.75 | 2.82 | 173654.47 | 104434.33 | 69220.14 | 1.51 | 29.45 |
| DALYs | Austria | Both | 72401.15 | 55710.82 | 16690.33 | 3.34 | 86872.23 | 53471.13 | 33401.10 | 1.60 | 19.99 |
| DALYs | Azerbaijan | Both | 30145.60 | 25067.75 | 5077.85 | 4.94 | 53993.19 | 43604.19 | 10389.00 | 4.20 | 79.11 |
| DALYs | Bahamas | Both | 635.88 | 486.21 | 149.67 | 3.25 | 1259.68 | 867.81 | 391.87 | 2.21 | 98.10 |
| DALYs | Bahrain | Both | 1309.06 | 1040.75 | 268.31 | 3.88 | 2212.32 | 1705.88 | 506.45 | 3.37 | 69.00 |
| DALYs | Bangladesh | Both | 295109.27 | 228410.51 | 66698.76 | 3.42 | 608826.51 | 440288.82 | 168537.69 | 2.61 | 106.31 |
| DALYs | Barbados | Both | 563.91 | 404.26 | 159.65 | 2.53 | 842.97 | 541.55 | 301.42 | 1.80 | 49.49 |
| DALYs | Barbuda | Both | 98.97 | 72.86 | 26.10 | 2.79 | 178.67 | 109.15 | 69.51 | 1.57 | 80.53 |
| DALYs | Belarus | Both | 104478.52 | 92671.09 | 11807.43 | 7.85 | 76376.97 | 67105.67 | 9271.29 | 7.24 | -26.90 |
| DALYs | Belgium | Both | 154992.26 | 133124.15 | 21868.11 | 6.09 | 143489.80 | 99777.65 | 43712.15 | 2.28 | -7.42 |
| DALYs | Belize | Both | 217.41 | 161.18 | 56.23 | 2.87 | 805.95 | 573.22 | 232.73 | 2.46 | 270.71 |
| DALYs | Benin | Both | 4623.92 | 3417.70 | 1206.22 | 2.83 | 9658.66 | 6857.09 | 2801.57 | 2.45 | 108.88 |
| DALYs | Bermuda | Both | 452.43 | 329.48 | 122.96 | 2.68 | 527.92 | 380.43 | 147.49 | 2.58 | 16.68 |
| DALYs | Bhutan | Both | 554.75 | 394.98 | 159.77 | 2.47 | 1051.22 | 720.56 | 330.66 | 2.18 | 89.49 |
| DALYs | Bolivia | Both | 10217.84 | 6980.04 | 3237.80 | 2.16 | 22343.84 | 13252.00 | 9091.84 | 1.46 | 118.67 |
| DALYs | Bosnia and Herzegovina | Both | 40532.46 | 34387.08 | 6145.38 | 5.60 | 51713.56 | 41028.29 | 10685.27 | 3.84 | 27.59 |
| DALYs | Botswana | Both | 2412.69 | 1830.97 | 581.72 | 3.15 | 3778.91 | 2369.26 | 1409.65 | 1.68 | 56.63 |
| DALYs | Brazil | Both | 359083.72 | 246685.98 | 112397.74 | 2.19 | 732579.09 | 419749.84 | 312829.25 | 1.34 | 104.01 |
| DALYs | Brunei | Both | 830.26 | 515.76 | 314.50 | 1.64 | 2109.23 | 1158.20 | 951.03 | 1.22 | 154.04 |
| DALYs | Bulgaria | Both | 83871.25 | 72320.71 | 11550.54 | 6.26 | 92074.18 | 73837.70 | 18236.48 | 4.05 | 9.78 |
| DALYs | Burkina Faso | Both | 8091.70 | 5519.49 | 2572.22 | 2.15 | 15342.98 | 10477.57 | 4865.41 | 2.15 | 89.61 |
| DALYs | Burundi | Both | 5245.29 | 3952.01 | 1293.28 | 3.06 | 6922.03 | 5348.24 | 1573.79 | 3.40 | 31.97 |
| DALYs | Cambodia | Both | 29754.71 | 21901.56 | 7853.15 | 2.79 | 56351.56 | 40879.14 | 15472.42 | 2.64 | 89.39 |
| DALYs | Cameroon | Both | 11554.78 | 8641.08 | 2913.69 | 2.97 | 28528.46 | 21160.57 | 7367.89 | 2.87 | 146.90 |
| DALYs | Canada | Both | 327786.45 | 218215.02 | 109571.43 | 1.99 | 416159.79 | 225835.71 | 190324.07 | 1.19 | 26.96 |
| DALYs | Cape Verde | Both | 454.67 | 261.38 | 193.29 | 1.35 | 1049.68 | 651.91 | 397.77 | 1.64 | 130.86 |
| DALYs | Central African Republic | Both | 4174.01 | 3273.20 | 900.82 | 3.63 | 6592.03 | 5262.13 | 1329.90 | 3.96 | 57.93 |
| DALYs | Chad | Both | 5698.58 | 4015.51 | 1683.07 | 2.39 | 13949.58 | 10496.32 | 3453.26 | 3.04 | 144.79 |
| DALYs | Chile | Both | 41980.15 | 30492.41 | 11487.75 | 2.65 | 72694.30 | 42972.62 | 29721.68 | 1.45 | 73.16 |
| DALYs | China | Both | 6348558.58 | 4372711.69 | 1975846.89 | 2.21 | 15252918.02 | 10671550.29 | 4581367.73 | 2.33 | 140.26 |
| DALYs | Colombia | Both | 59583.94 | 38598.61 | 20985.33 | 1.84 | 115144.68 | 66653.85 | 48490.83 | 1.37 | 93.25 |
| DALYs | Comoros | Both | 543.46 | 371.54 | 171.92 | 2.16 | 829.78 | 544.51 | 285.28 | 1.91 | 52.69 |
| DALYs | Costa Rica | Both | 4416.92 | 3172.15 | 1244.76 | 2.55 | 9530.97 | 6661.92 | 2869.05 | 2.32 | 115.78 |
| DALYs | Croatia | Both | 64240.27 | 55225.64 | 9014.64 | 6.13 | 59565.16 | 45249.97 | 14315.19 | 3.16 | -7.28 |
| DALYs | Cuba | Both | 71552.59 | 51221.69 | 20330.90 | 2.52 | 124566.47 | 78836.29 | 45730.18 | 1.72 | 74.09 |
| DALYs | Cyprus | Both | 3827.61 | 3114.32 | 713.29 | 4.37 | 8780.92 | 7096.30 | 1684.63 | 4.21 | 129.41 |
| DALYs | Czech Republic | Both | 157487.60 | 135836.79 | 21650.80 | 6.27 | 120176.90 | 83729.96 | 36446.94 | 2.30 | -23.69 |
| DALYs | Democratic Republic of the Congo | Both | 41286.20 | 30175.61 | 11110.60 | 2.72 | 73643.41 | 53671.26 | 19972.14 | 2.69 | 78.37 |
| DALYs | Denmark | Both | 74729.38 | 47691.54 | 27037.83 | 1.76 | 77653.43 | 40908.57 | 36744.85 | 1.11 | 3.91 |
| DALYs | Djibouti | Both | 397.11 | 276.76 | 120.35 | 2.30 | 1311.68 | 984.60 | 327.09 | 3.01 | 230.30 |
| DALYs | Dominica | Both | 192.59 | 138.95 | 53.64 | 2.59 | 277.41 | 193.49 | 83.92 | 2.31 | 44.04 |
| DALYs | Dominican Republic | Both | 9186.64 | 5865.66 | 3320.98 | 1.77 | 26882.11 | 16719.54 | 10162.58 | 1.65 | 192.62 |
| DALYs | Ecuador | Both | 10257.83 | 6818.55 | 3439.28 | 1.98 | 26788.76 | 14830.83 | 11957.93 | 1.24 | 161.15 |
| DALYs | Egypt | Both | 48140.67 | 34178.15 | 13962.53 | 2.45 | 121823.93 | 86630.28 | 35193.65 | 2.46 | 153.06 |
| DALYs | El Salvador | Both | 4904.04 | 3068.91 | 1835.13 | 1.67 | 11199.23 | 6463.53 | 4735.70 | 1.36 | 128.37 |
| DALYs | Equatorial Guinea | Both | 625.86 | 497.06 | 128.80 | 3.86 | 1217.70 | 770.43 | 447.26 | 1.72 | 94.57 |
| DALYs | Eritrea | Both | 2841.02 | 2187.63 | 653.39 | 3.35 | 5422.87 | 3782.83 | 1640.04 | 2.31 | 90.88 |
| DALYs | Estonia | Both | 19598.03 | 16795.80 | 2802.23 | 5.99 | 13366.65 | 10214.34 | 3152.30 | 3.24 | -31.80 |
| DALYs | Ethiopia | Both | 60191.76 | 51084.09 | 9107.67 | 5.61 | 71490.45 | 52143.73 | 19346.72 | 2.70 | 18.77 |
| DALYs | Fiji | Both | 754.35 | 493.11 | 261.24 | 1.89 | 1373.93 | 907.80 | 466.13 | 1.95 | 82.14 |
| DALYs | Finland | Both | 43826.30 | 35168.58 | 8657.73 | 4.06 | 45680.82 | 30301.60 | 15379.22 | 1.97 | 4.23 |
| DALYs | France | Both | 570129.23 | 497856.59 | 72272.63 | 6.89 | 787563.42 | 566166.69 | 221396.73 | 2.56 | 38.14 |
| DALYs | Gabon | Both | 2095.56 | 1628.68 | 466.88 | 3.49 | 3545.54 | 2727.49 | 818.05 | 3.33 | 69.19 |
| DALYs | Gambia | Both | 675.65 | 523.56 | 152.09 | 3.44 | 1617.56 | 1159.97 | 457.59 | 2.53 | 139.41 |
| DALYs | Georgia | Both | 35547.51 | 29620.14 | 5927.36 | 5.00 | 37466.72 | 32393.64 | 5073.08 | 6.39 | 5.40 |
| DALYs | Germany | Both | 899611.46 | 725649.31 | 173962.15 | 4.17 | 1033252.88 | 674326.83 | 358926.05 | 1.88 | 14.86 |
| DALYs | Ghana | Both | 9778.98 | 6892.76 | 2886.22 | 2.39 | 24977.19 | 17427.48 | 7549.70 | 2.31 | 155.42 |
| DALYs | Greece | Both | 118318.40 | 100848.13 | 17470.28 | 5.77 | 147060.28 | 114900.71 | 32159.58 | 3.57 | 24.29 |
| DALYs | Greenland | Both | 914.89 | 566.18 | 348.71 | 1.62 | 1144.65 | 736.12 | 408.53 | 1.80 | 25.11 |
| DALYs | Grenada | Both | 193.98 | 136.43 | 57.55 | 2.37 | 410.75 | 266.37 | 144.38 | 1.84 | 111.75 |
| DALYs | Grenadines | Both | 148.20 | 96.25 | 51.95 | 1.85 | 312.61 | 209.62 | 102.99 | 2.04 | 110.95 |
| DALYs | Guam | Both | 698.63 | 542.25 | 156.38 | 3.47 | 1563.63 | 1053.71 | 509.92 | 2.07 | 123.81 |
| DALYs | Guatemala | Both | 7292.02 | 4214.90 | 3077.12 | 1.37 | 16127.37 | 10163.61 | 5963.76 | 1.70 | 121.16 |
| DALYs | Guinea | Both | 7066.96 | 5332.73 | 1734.23 | 3.07 | 12750.92 | 10135.22 | 2615.70 | 3.87 | 80.43 |
| DALYs | Guinea-Bissau | Both | 1311.96 | 1048.07 | 263.89 | 3.97 | 1773.63 | 1301.24 | 472.40 | 2.75 | 35.19 |
| DALYs | Guyana | Both | 657.80 | 454.31 | 203.49 | 2.23 | 1150.36 | 694.68 | 455.68 | 1.52 | 74.88 |
| DALYs | Haiti | Both | 13885.18 | 10621.38 | 3263.80 | 3.25 | 20671.69 | 14548.32 | 6123.37 | 2.38 | 48.88 |
| DALYs | Honduras | Both | 4046.43 | 2752.94 | 1293.49 | 2.13 | 10821.43 | 6654.03 | 4167.40 | 1.60 | 167.43 |
| DALYs | Hungary | Both | 175487.08 | 140124.64 | 35362.44 | 3.96 | 195082.25 | 126374.84 | 68707.40 | 1.84 | 11.17 |
| DALYs | Iceland | Both | 2028.77 | 1126.78 | 901.99 | 1.25 | 2840.65 | 1570.55 | 1270.10 | 1.24 | 40.02 |
| DALYs | India | Both | 896761.14 | 714543.64 | 182217.50 | 3.92 | 2059937.63 | 1451186.85 | 608750.78 | 2.38 | 129.71 |
| DALYs | Indonesia | Both | 434067.91 | 312524.50 | 121543.40 | 2.57 | 925031.59 | 695790.77 | 229240.82 | 3.04 | 113.11 |
| DALYs | Iran | Both | 68588.99 | 51506.09 | 17082.90 | 3.02 | 174173.70 | 120144.10 | 54029.60 | 2.22 | 153.94 |
| DALYs | Iraq | Both | 34065.14 | 27027.79 | 7037.35 | 3.84 | 52636.30 | 36698.37 | 15937.94 | 2.30 | 54.52 |
| DALYs | Ireland | Both | 33337.07 | 22756.44 | 10580.63 | 2.15 | 38633.51 | 21567.03 | 17066.47 | 1.26 | 15.89 |
| DALYs | Israel | Both | 22393.09 | 15612.88 | 6780.20 | 2.30 | 44117.78 | 29912.49 | 14205.28 | 2.11 | 97.02 |
| DALYs | Italy | Both | 719408.15 | 606614.23 | 112793.92 | 5.38 | 612436.19 | 436260.61 | 176175.59 | 2.48 | -14.87 |
| DALYs | Ivory Coast | Both | 7351.47 | 5644.87 | 1706.59 | 3.31 | 15330.85 | 11512.04 | 3818.81 | 3.01 | 108.54 |
| DALYs | Jamaica | Both | 5977.89 | 4673.00 | 1304.89 | 3.58 | 13401.24 | 10860.65 | 2540.59 | 4.27 | 124.18 |
| DALYs | Japan | Both | 769509.82 | 565834.35 | 203675.47 | 2.78 | 1198319.93 | 875751.44 | 322568.49 | 2.71 | 55.73 |
| DALYs | Jordan | Both | 5114.26 | 4286.03 | 828.24 | 5.17 | 17251.21 | 13436.74 | 3814.47 | 3.52 | 237.32 |
| DALYs | Kazakhstan | Both | 139632.39 | 117083.42 | 22548.97 | 5.19 | 83698.06 | 68085.32 | 15612.74 | 4.36 | -40.06 |
| DALYs | Kenya | Both | 12134.32 | 8750.23 | 3384.09 | 2.59 | 29090.30 | 21660.68 | 7429.62 | 2.92 | 139.74 |
| DALYs | Kiribati | Both | 156.06 | 101.75 | 54.30 | 1.87 | 289.09 | 195.27 | 93.82 | 2.08 | 85.24 |
| DALYs | Kuwait | Both | 1776.92 | 1368.70 | 408.22 | 3.35 | 3854.72 | 2970.14 | 884.58 | 3.36 | 116.93 |
| DALYs | Kyrgyzstan | Both | 20173.73 | 16713.45 | 3460.28 | 4.83 | 12987.46 | 9887.83 | 3099.63 | 3.19 | -35.62 |
| DALYs | Laos | Both | 14856.57 | 11204.48 | 3652.09 | 3.07 | 22270.94 | 15706.10 | 6564.83 | 2.39 | 49.91 |
| DALYs | Latvia | Both | 31476.44 | 26988.55 | 4487.89 | 6.01 | 20813.33 | 16777.87 | 4035.46 | 4.16 | -33.88 |
| DALYs | Lebanon | Both | 15567.88 | 12051.12 | 3516.76 | 3.43 | 31836.42 | 18789.08 | 13047.34 | 1.44 | 104.50 |
| DALYs | Lesotho | Both | 3493.55 | 2862.65 | 630.90 | 4.54 | 5269.65 | 4194.20 | 1075.45 | 3.90 | 50.84 |
| DALYs | Liberia | Both | 2276.37 | 1764.31 | 512.07 | 3.45 | 3231.58 | 2338.69 | 892.89 | 2.62 | 41.96 |
| DALYs | Libya | Both | 10530.18 | 9575.68 | 954.51 | 10.03 | 24321.54 | 21334.91 | 2986.63 | 7.14 | 130.97 |
| DALYs | Lithuania | Both | 38078.18 | 33291.97 | 4786.21 | 6.96 | 30537.84 | 25238.40 | 5299.44 | 4.76 | -19.80 |
| DALYs | Luxembourg | Both | 4837.25 | 4022.55 | 814.70 | 4.94 | 5811.45 | 3839.49 | 1971.96 | 1.95 | 20.14 |
| DALYs | Macedonia | Both | 12680.66 | 10749.86 | 1930.80 | 5.57 | 25744.73 | 21335.24 | 4409.50 | 4.84 | 103.02 |
| DALYs | Madagascar | Both | 10550.90 | 7633.46 | 2917.44 | 2.62 | 18002.63 | 12958.75 | 5043.88 | 2.57 | 70.63 |
| DALYs | Malawi | Both | 5673.65 | 3953.83 | 1719.82 | 2.30 | 9255.31 | 6542.53 | 2712.78 | 2.41 | 63.13 |
| DALYs | Malaysia | Both | 40547.08 | 30502.97 | 10044.11 | 3.04 | 108134.96 | 78376.22 | 29758.74 | 2.63 | 166.69 |
| DALYs | Maldives | Both | 308.81 | 270.02 | 38.78 | 6.96 | 444.63 | 359.07 | 85.56 | 4.20 | 43.98 |
| DALYs | Mali | Both | 7109.81 | 5122.71 | 1987.11 | 2.58 | 12783.61 | 9035.64 | 3747.97 | 2.41 | 79.80 |
| DALYs | Malta | Both | 2418.63 | 2092.21 | 326.42 | 6.41 | 3941.56 | 3143.03 | 798.53 | 3.94 | 62.97 |
| DALYs | Marshall Islands | Both | 111.77 | 94.12 | 17.65 | 5.33 | 231.32 | 186.24 | 45.08 | 4.13 | 106.95 |
| DALYs | Mauritania | Both | 2481.58 | 1742.10 | 739.48 | 2.36 | 4059.94 | 2708.85 | 1351.09 | 2.00 | 63.60 |
| DALYs | Mauritius | Both | 2411.24 | 1838.85 | 572.38 | 3.21 | 4346.26 | 3127.92 | 1218.34 | 2.57 | 80.25 |
| DALYs | Mexico | Both | 131270.50 | 90733.25 | 40537.25 | 2.24 | 203586.73 | 133505.58 | 70081.15 | 1.91 | 55.09 |
| DALYs | Micronesia | Both | 301.17 | 219.21 | 81.96 | 2.67 | 418.12 | 300.92 | 117.20 | 2.57 | 38.83 |
| DALYs | Moldova | Both | 33925.04 | 28268.82 | 5656.23 | 5.00 | 27629.98 | 22821.04 | 4808.94 | 4.75 | -18.56 |
| DALYs | Mongolia | Both | 9086.46 | 6896.84 | 2189.62 | 3.15 | 12747.56 | 10138.16 | 2609.40 | 3.89 | 40.29 |
| DALYs | Montenegro | Both | 6894.04 | 5375.64 | 1518.40 | 3.54 | 10397.41 | 7762.09 | 2635.32 | 2.95 | 50.82 |
| DALYs | Morocco | Both | 50375.48 | 44615.32 | 5760.15 | 7.75 | 112535.94 | 99197.18 | 13338.76 | 7.44 | 123.39 |
| DALYs | Mozambique | Both | 12749.73 | 9993.55 | 2756.18 | 3.63 | 25570.43 | 20615.03 | 4955.40 | 4.16 | 100.56 |
| DALYs | Myanmar | Both | 151097.32 | 105357.52 | 45739.80 | 2.30 | 212783.26 | 138252.31 | 74530.95 | 1.85 | 40.83 |
| DALYs | Namibia | Both | 1598.59 | 1133.25 | 465.34 | 2.44 | 2319.61 | 1594.41 | 725.21 | 2.20 | 45.10 |
| DALYs | Nepal | Both | 28783.20 | 19275.04 | 9508.16 | 2.03 | 54760.72 | 31886.51 | 22874.21 | 1.39 | 90.25 |
| DALYs | Netherlands | Both | 199965.14 | 166162.81 | 33802.33 | 4.92 | 239454.92 | 136385.35 | 103069.57 | 1.32 | 19.75 |
| DALYs | New Zealand | Both | 29099.88 | 19208.08 | 9891.80 | 1.94 | 35966.29 | 18791.56 | 17174.73 | 1.09 | 23.60 |
| DALYs | Nicaragua | Both | 2677.53 | 1776.66 | 900.87 | 1.97 | 6121.56 | 3778.40 | 2343.16 | 1.61 | 128.63 |
| DALYs | Niger | Both | 5869.61 | 4361.13 | 1508.48 | 2.89 | 13534.96 | 9587.71 | 3947.24 | 2.43 | 130.59 |
| DALYs | Nigeria | Both | 82163.59 | 64194.06 | 17969.52 | 3.57 | 137580.92 | 91909.47 | 45671.46 | 2.01 | 67.45 |
| DALYs | North Korea | Both | 107144.07 | 73276.64 | 33867.43 | 2.16 | 187258.98 | 138655.94 | 48603.03 | 2.85 | 74.77 |
| DALYs | Northern Mariana Islands | Both | 171.71 | 139.65 | 32.06 | 4.36 | 360.67 | 275.16 | 85.51 | 3.22 | 110.05 |
| DALYs | Norway | Both | 34037.66 | 24309.07 | 9728.59 | 2.50 | 41188.60 | 23385.86 | 17802.74 | 1.31 | 21.01 |
| DALYs | Oman | Both | 1391.61 | 1135.34 | 256.27 | 4.43 | 3075.18 | 2320.85 | 754.33 | 3.08 | 120.98 |
| DALYs | Pakistan | Both | 181397.16 | 147764.42 | 33632.74 | 4.39 | 406517.96 | 318810.27 | 87707.69 | 3.63 | 124.10 |
| DALYs | Palestine | Both | 3825.72 | 2948.07 | 877.65 | 3.36 | 9208.99 | 6755.42 | 2453.56 | 2.75 | 140.71 |
| DALYs | Panama | Both | 4678.95 | 3574.32 | 1104.63 | 3.24 | 7941.07 | 5153.10 | 2787.97 | 1.85 | 69.72 |
| DALYs | Papua New Guinea | Both | 14017.52 | 10988.56 | 3028.96 | 3.63 | 33758.80 | 26228.23 | 7530.57 | 3.48 | 140.83 |
| DALYs | Paraguay | Both | 5817.65 | 4006.96 | 1810.69 | 2.21 | 17468.78 | 12987.14 | 4481.64 | 2.90 | 200.27 |
| DALYs | Peru | Both | 37523.07 | 23614.80 | 13908.27 | 1.70 | 62099.85 | 33919.41 | 28180.43 | 1.20 | 65.50 |
| DALYs | Philippines | Both | 143529.80 | 107212.16 | 36317.64 | 2.95 | 328392.28 | 231581.14 | 96811.14 | 2.39 | 128.80 |
| DALYs | Poland | Both | 479949.81 | 401143.68 | 78806.14 | 5.09 | 599114.84 | 422681.52 | 176433.32 | 2.40 | 24.83 |
| DALYs | Portugal | Both | 61494.55 | 50403.44 | 11091.11 | 4.54 | 92277.04 | 71169.94 | 21107.10 | 3.37 | 50.06 |
| DALYs | Puerto Rico | Both | 12254.37 | 8543.09 | 3711.28 | 2.30 | 14600.23 | 9182.78 | 5417.46 | 1.70 | 19.14 |
| DALYs | Qatar | Both | 541.67 | 464.52 | 77.16 | 6.02 | 2639.89 | 2233.15 | 406.73 | 5.49 | 387.36 |
| DALYs | Republic of Congo | Both | 4427.47 | 3285.23 | 1142.24 | 2.88 | 7786.51 | 5267.16 | 2519.35 | 2.09 | 75.87 |
| DALYs | Romania | Both | 196051.51 | 165988.70 | 30062.81 | 5.52 | 252460.28 | 200318.90 | 52141.38 | 3.84 | 28.77 |
| DALYs | Russia | Both | 1609315.65 | 1384862.76 | 224452.89 | 6.17 | 1217403.74 | 1012105.58 | 205298.16 | 4.93 | -24.35 |
| DALYs | Rwanda | Both | 6839.66 | 5159.36 | 1680.30 | 3.07 | 9177.43 | 6221.01 | 2956.42 | 2.10 | 34.18 |
| DALYs | Saint Lucia | Both | 256.25 | 174.33 | 81.92 | 2.13 | 528.42 | 341.67 | 186.75 | 1.83 | 106.21 |
| DALYs | Saint Vincent | Both | 148.20 | 96.25 | 51.95 | 1.85 | 312.61 | 209.62 | 102.99 | 2.04 | 110.95 |
| DALYs | Samoa | Both | 169.46 | 125.75 | 43.71 | 2.88 | 242.30 | 170.16 | 72.14 | 2.36 | 42.99 |
| DALYs | Sao Tome and Principe | Both | 188.43 | 140.85 | 47.58 | 2.96 | 359.09 | 266.26 | 92.83 | 2.87 | 90.57 |
| DALYs | Saudi Arabia | Both | 11650.79 | 10049.46 | 1601.34 | 6.28 | 33772.66 | 26698.09 | 7074.56 | 3.77 | 189.87 |
| DALYs | Senegal | Both | 8952.54 | 7073.41 | 1879.12 | 3.76 | 17779.66 | 13336.80 | 4442.86 | 3.00 | 98.60 |
| DALYs | Serbia | Both | 130288.34 | 106580.08 | 23708.26 | 4.50 | 153765.80 | 109821.46 | 43944.35 | 2.50 | 18.02 |
| DALYs | Seychelles | Both | 213.49 | 171.56 | 41.94 | 4.09 | 388.49 | 303.51 | 84.98 | 3.57 | 81.97 |
| DALYs | Sierra Leone | Both | 4653.13 | 3543.19 | 1109.94 | 3.19 | 7566.26 | 5543.59 | 2022.67 | 2.74 | 62.61 |
| DALYs | Singapore | Both | 18652.41 | 13560.33 | 5092.08 | 2.66 | 25050.65 | 17289.36 | 7761.29 | 2.23 | 34.30 |
| DALYs | Slovakia | Both | 57683.46 | 51248.91 | 6434.55 | 7.96 | 53209.32 | 40406.26 | 12803.06 | 3.16 | -7.76 |
| DALYs | Slovenia | Both | 21876.94 | 18279.20 | 3597.74 | 5.08 | 24368.86 | 17480.84 | 6888.02 | 2.54 | 11.39 |
| DALYs | Solomon Islands | Both | 889.23 | 714.04 | 175.19 | 4.08 | 1687.51 | 1275.16 | 412.35 | 3.09 | 89.77 |
| DALYs | Somalia | Both | 5492.30 | 4063.39 | 1428.90 | 2.84 | 12025.38 | 8934.12 | 3091.26 | 2.89 | 118.95 |
| DALYs | South Africa | Both | 104707.50 | 74946.94 | 29760.56 | 2.52 | 180039.79 | 127443.47 | 52596.33 | 2.42 | 71.95 |
| DALYs | South Korea | Both | 169746.04 | 128306.71 | 41439.33 | 3.10 | 362958.51 | 265942.19 | 97016.32 | 2.74 | 113.82 |
| DALYs | South Sudan | Both | 5868.57 | 4568.98 | 1299.59 | 3.52 | 8696.97 | 6670.70 | 2026.27 | 3.29 | 48.20 |
| DALYs | Spain | Both | 354802.86 | 320220.20 | 34582.66 | 9.26 | 464559.14 | 362614.45 | 101944.69 | 3.56 | 30.93 |
| DALYs | Sri Lanka | Both | 20755.49 | 15620.88 | 5134.61 | 3.04 | 41250.58 | 29964.22 | 11286.36 | 2.65 | 98.75 |
| DALYs | Sudan | Both | 19619.79 | 15101.54 | 4518.25 | 3.34 | 34385.11 | 25300.86 | 9084.26 | 2.79 | 75.26 |
| DALYs | Suriname | Both | 784.84 | 535.99 | 248.85 | 2.15 | 1994.51 | 1292.96 | 701.55 | 1.84 | 154.13 |
| DALYs | Swaziland | Both | 1384.08 | 1086.21 | 297.88 | 3.65 | 2410.51 | 1896.86 | 513.65 | 3.69 | 74.16 |
| DALYs | Sweden | Both | 60479.12 | 39838.18 | 20640.94 | 1.93 | 71551.32 | 35106.23 | 36445.09 | 0.96 | 18.31 |
| DALYs | Switzerland | Both | 66642.89 | 53429.31 | 13213.59 | 4.04 | 67056.51 | 40753.88 | 26302.63 | 1.55 | 0.62 |
| DALYs | Syria | Both | 13765.22 | 10401.78 | 3363.44 | 3.09 | 31384.58 | 23889.66 | 7494.92 | 3.19 | 128.00 |
| DALYs | Taiwan (Province of China) | Both | 89177.01 | 62566.55 | 26610.46 | 2.35 | 208650.88 | 132412.27 | 76238.62 | 1.74 | 133.97 |
| DALYs | Tajikistan | Both | 10458.09 | 7596.69 | 2861.40 | 2.65 | 11008.91 | 7135.56 | 3873.35 | 1.84 | 5.27 |
| DALYs | Tanzania | Both | 18590.29 | 13499.89 | 5090.40 | 2.65 | 38002.59 | 26811.09 | 11191.50 | 2.40 | 104.42 |
| DALYs | Thailand | Both | 296035.28 | 185078.26 | 110957.01 | 1.67 | 431745.74 | 293324.69 | 138421.05 | 2.12 | 45.84 |
| DALYs | Timor-Leste | Both | 1282.67 | 906.58 | 376.09 | 2.41 | 3404.85 | 2486.78 | 918.07 | 2.71 | 165.45 |
| DALYs | Tobago | Both | 2368.17 | 1806.76 | 561.41 | 3.22 | 4311.19 | 3218.69 | 1092.50 | 2.95 | 82.05 |
| DALYs | Togo | Both | 2766.56 | 1971.83 | 794.73 | 2.48 | 7369.55 | 5294.05 | 2075.50 | 2.55 | 166.38 |
| DALYs | Tonga | Both | 360.72 | 280.59 | 80.13 | 3.50 | 474.64 | 372.07 | 102.57 | 3.63 | 31.58 |
| DALYs | Trinidad | Both | 2368.17 | 1806.76 | 561.41 | 3.22 | 4311.19 | 3218.69 | 1092.50 | 2.95 | 82.05 |
| DALYs | Tunisia | Both | 20497.33 | 18871.61 | 1625.71 | 11.61 | 47744.07 | 42757.64 | 4986.43 | 8.57 | 132.93 |
| DALYs | Turkey | Both | 408182.52 | 356069.09 | 52113.43 | 6.83 | 661190.22 | 569455.06 | 91735.16 | 6.21 | 61.98 |
| DALYs | Turkmenistan | Both | 8293.16 | 6625.88 | 1667.28 | 3.97 | 10793.57 | 8222.65 | 2570.92 | 3.20 | 30.15 |
| DALYs | Uganda | Both | 8638.96 | 5361.43 | 3277.53 | 1.64 | 20110.81 | 13200.25 | 6910.56 | 1.91 | 132.79 |
| DALYs | UK | Both | 835674.57 | 565327.10 | 270347.47 | 2.09 | 705875.56 | 389446.83 | 316428.73 | 1.23 | -15.53 |
| DALYs | Ukraine | Both | 641094.25 | 542147.94 | 98946.31 | 5.48 | 398393.15 | 337238.49 | 61154.66 | 5.51 | -37.86 |
| DALYs | United Arab Emirates | Both | 1483.45 | 1219.86 | 263.59 | 4.63 | 13711.01 | 11709.97 | 2001.04 | 5.85 | 824.26 |
| DALYs | Uruguay | Both | 35403.94 | 31036.92 | 4367.02 | 7.11 | 34177.33 | 25282.45 | 8894.88 | 2.84 | -3.46 |
| DALYs | USA | Both | 3357006.35 | 2144860.98 | 1212145.37 | 1.77 | 3765845.00 | 2125996.20 | 1639848.80 | 1.30 | 12.18 |
| DALYs | Uzbekistan | Both | 48957.05 | 37090.61 | 11866.43 | 3.13 | 62216.19 | 45749.73 | 16466.45 | 2.78 | 27.08 |
| DALYs | Vanuatu | Both | 444.40 | 342.38 | 102.01 | 3.36 | 1017.85 | 765.89 | 251.96 | 3.04 | 129.04 |
| DALYs | Venezuela | Both | 42443.57 | 27416.33 | 15027.24 | 1.82 | 111889.66 | 69606.85 | 42282.82 | 1.65 | 163.62 |
| DALYs | Vietnam | Both | 352965.89 | 251779.23 | 101186.66 | 2.49 | 866747.31 | 623280.26 | 243467.05 | 2.56 | 145.56 |
| DALYs | Virgin Islands | Both | 379.74 | 268.25 | 111.49 | 2.41 | 899.03 | 625.15 | 273.89 | 2.28 | 136.75 |
| DALYs | Yemen | Both | 13977.49 | 10834.42 | 3143.07 | 3.45 | 32185.56 | 24225.62 | 7959.94 | 3.04 | 130.27 |
| DALYs | Zambia | Both | 6256.71 | 4740.87 | 1515.84 | 3.13 | 11578.61 | 8504.89 | 3073.73 | 2.77 | 85.06 |
| DALYs | Zimbabwe | Both | 12854.06 | 10046.08 | 2807.98 | 3.58 | 19363.54 | 13179.33 | 6184.21 | 2.13 | 50.64 |
| Deaths | Afghanistan | Both | 956.57 | 777.78 | 178.79 | 4.35 | 1208.87 | 892.32 | 316.55 | 2.82 | 26.38 |
| Deaths | Albania | Both | 540.10 | 456.97 | 83.14 | 5.50 | 1039.43 | 867.88 | 171.54 | 5.06 | 92.45 |
| Deaths | Algeria | Both | 1294.32 | 1086.43 | 207.89 | 5.23 | 2757.79 | 2220.73 | 537.06 | 4.14 | 113.07 |
| Deaths | American Samoa | Both | 5.95 | 4.66 | 1.29 | 3.61 | 10.05 | 7.14 | 2.91 | 2.45 | 68.77 |
| Deaths | Andorra | Both | 19.79 | 15.63 | 4.17 | 3.75 | 35.09 | 23.94 | 11.16 | 2.15 | 77.30 |
| Deaths | Angola | Both | 561.56 | 452.86 | 108.70 | 4.17 | 1135.03 | 850.25 | 284.78 | 2.99 | 102.12 |
| Deaths | Antigua | Both | 4.48 | 3.30 | 1.18 | 2.81 | 7.93 | 4.82 | 3.10 | 1.55 | 76.92 |
| Deaths | Argentina | Both | 8604.06 | 6962.41 | 1641.65 | 4.24 | 11292.00 | 7621.25 | 3670.75 | 2.08 | 31.24 |
| Deaths | Armenia | Both | 857.29 | 722.53 | 134.76 | 5.36 | 1234.92 | 1021.41 | 213.52 | 4.78 | 44.05 |
| Deaths | Australia | Both | 6147.07 | 4549.39 | 1597.69 | 2.85 | 9148.02 | 5568.38 | 3579.64 | 1.56 | 48.82 |
| Deaths | Austria | Both | 3297.49 | 2479.81 | 817.68 | 3.03 | 4202.86 | 2594.21 | 1608.66 | 1.61 | 27.46 |
| Deaths | Azerbaijan | Both | 1022.68 | 835.50 | 187.17 | 4.46 | 1905.92 | 1538.23 | 367.69 | 4.18 | 86.37 |
| Deaths | Bahamas | Both | 24.35 | 18.51 | 5.85 | 3.16 | 49.49 | 33.83 | 15.66 | 2.16 | 103.20 |
| Deaths | Bahrain | Both | 57.51 | 46.03 | 11.48 | 4.01 | 90.17 | 68.83 | 21.33 | 3.23 | 56.77 |
| Deaths | Bangladesh | Both | 10721.03 | 8640.73 | 2080.29 | 4.15 | 25225.83 | 19690.41 | 5535.42 | 3.56 | 135.29 |
| Deaths | Barbados | Both | 27.83 | 19.87 | 7.96 | 2.50 | 40.08 | 25.49 | 14.59 | 1.75 | 44.03 |
| Deaths | Barbuda | Both | 4.48 | 3.30 | 1.18 | 2.81 | 7.93 | 4.82 | 3.10 | 1.55 | 76.92 |
| Deaths | Belarus | Both | 3912.73 | 3384.61 | 528.13 | 6.41 | 3161.85 | 2717.55 | 444.30 | 6.12 | -19.19 |
| Deaths | Belgium | Both | 7219.07 | 6195.74 | 1023.34 | 6.05 | 7243.07 | 5133.50 | 2109.57 | 2.43 | 0.33 |
| Deaths | Belize | Both | 9.52 | 7.10 | 2.42 | 2.93 | 31.21 | 22.45 | 8.76 | 2.56 | 227.73 |
| Deaths | Benin | Both | 199.96 | 150.58 | 49.38 | 3.05 | 402.00 | 289.77 | 112.23 | 2.58 | 101.04 |
| Deaths | Bermuda | Both | 19.78 | 14.17 | 5.61 | 2.53 | 26.97 | 18.70 | 8.26 | 2.26 | 36.31 |
| Deaths | Bhutan | Both | 20.73 | 14.82 | 5.92 | 2.50 | 44.14 | 31.10 | 13.04 | 2.39 | 112.92 |
| Deaths | Bolivia | Both | 406.87 | 281.25 | 125.62 | 2.24 | 1001.65 | 611.72 | 389.93 | 1.57 | 146.18 |
| Deaths | Bosnia and Herzegovina | Both | 1489.70 | 1253.12 | 236.58 | 5.30 | 2237.57 | 1759.25 | 478.32 | 3.68 | 50.20 |
| Deaths | Botswana | Both | 91.83 | 69.13 | 22.70 | 3.05 | 155.10 | 97.80 | 57.30 | 1.71 | 68.91 |
| Deaths | Brazil | Both | 14085.39 | 9702.39 | 4383.00 | 2.21 | 32406.36 | 18823.17 | 13583.19 | 1.39 | 130.07 |
| Deaths | Brunei | Both | 34.94 | 22.21 | 12.72 | 1.75 | 86.29 | 48.44 | 37.86 | 1.28 | 147.00 |
| Deaths | Bulgaria | Both | 3108.87 | 2646.20 | 462.67 | 5.72 | 3780.56 | 2989.67 | 790.89 | 3.78 | 21.61 |
| Deaths | Burkina Faso | Both | 339.50 | 237.93 | 101.56 | 2.34 | 625.96 | 435.12 | 190.83 | 2.28 | 84.38 |
| Deaths | Burundi | Both | 200.71 | 153.53 | 47.18 | 3.25 | 269.02 | 211.28 | 57.74 | 3.66 | 34.03 |
| Deaths | Cambodia | Both | 1102.41 | 818.92 | 283.50 | 2.89 | 2241.36 | 1627.29 | 614.07 | 2.65 | 103.31 |
| Deaths | Cameroon | Both | 461.49 | 350.91 | 110.58 | 3.17 | 1160.41 | 874.38 | 286.03 | 3.06 | 151.45 |
| Deaths | Canada | Both | 14750.56 | 9962.14 | 4788.42 | 2.08 | 21439.83 | 11750.09 | 9689.75 | 1.21 | 45.35 |
| Deaths | Cape Verde | Both | 20.12 | 11.93 | 8.19 | 1.46 | 49.46 | 30.29 | 19.17 | 1.58 | 145.84 |
| Deaths | Central African Republic | Both | 147.57 | 116.16 | 31.41 | 3.70 | 226.89 | 179.66 | 47.24 | 3.80 | 53.75 |
| Deaths | Chad | Both | 249.11 | 179.38 | 69.73 | 2.57 | 587.89 | 452.44 | 135.44 | 3.34 | 135.99 |
| Deaths | Chile | Both | 1772.72 | 1261.76 | 510.96 | 2.47 | 3611.80 | 2086.76 | 1525.04 | 1.37 | 103.74 |
| Deaths | China | Both | 240475.21 | 164447.66 | 76027.55 | 2.16 | 692388.62 | 477245.77 | 215142.84 | 2.22 | 187.93 |
| Deaths | Colombia | Both | 2414.60 | 1578.83 | 835.77 | 1.89 | 5570.99 | 3202.10 | 2368.89 | 1.35 | 130.72 |
| Deaths | Comoros | Both | 21.36 | 15.05 | 6.31 | 2.39 | 35.37 | 23.89 | 11.48 | 2.08 | 65.60 |
| Deaths | Costa Rica | Both | 196.61 | 142.63 | 53.97 | 2.64 | 460.28 | 321.76 | 138.52 | 2.32 | 134.11 |
| Deaths | Croatia | Both | 2476.79 | 2092.50 | 384.30 | 5.45 | 2729.65 | 2053.14 | 676.51 | 3.03 | 10.21 |
| Deaths | Cuba | Both | 3321.54 | 2406.45 | 915.09 | 2.63 | 5951.69 | 3799.25 | 2152.44 | 1.77 | 79.18 |
| Deaths | Cyprus | Both | 175.04 | 143.51 | 31.54 | 4.55 | 420.23 | 343.25 | 76.98 | 4.46 | 140.07 |
| Deaths | Czech Republic | Both | 6346.61 | 5397.57 | 949.04 | 5.69 | 5763.02 | 3973.80 | 1789.23 | 2.22 | -9.20 |
| Deaths | Democratic Republic of the Congo | Both | 1559.01 | 1159.75 | 399.26 | 2.90 | 2736.31 | 1979.51 | 756.80 | 2.62 | 75.52 |
| Deaths | Denmark | Both | 3442.56 | 2252.67 | 1189.89 | 1.89 | 4100.55 | 2168.42 | 1932.13 | 1.12 | 19.11 |
| Deaths | Djibouti | Both | 14.24 | 10.19 | 4.05 | 2.52 | 51.01 | 39.33 | 11.68 | 3.37 | 258.29 |
| Deaths | Dominica | Both | 9.10 | 6.49 | 2.61 | 2.49 | 13.00 | 8.81 | 4.19 | 2.10 | 42.84 |
| Deaths | Dominican Republic | Both | 365.51 | 237.00 | 128.51 | 1.84 | 1182.43 | 748.96 | 433.47 | 1.73 | 223.50 |
| Deaths | Ecuador | Both | 424.72 | 288.55 | 136.18 | 2.12 | 1290.67 | 733.69 | 556.98 | 1.32 | 203.89 |
| Deaths | Egypt | Both | 1642.38 | 1220.85 | 421.53 | 2.90 | 4207.84 | 3109.70 | 1098.14 | 2.83 | 156.20 |
| Deaths | El Salvador | Both | 201.74 | 126.72 | 75.02 | 1.69 | 525.11 | 304.23 | 220.88 | 1.38 | 160.30 |
| Deaths | Equatorial Guinea | Both | 22.87 | 18.15 | 4.72 | 3.85 | 47.07 | 30.41 | 16.66 | 1.83 | 105.79 |
| Deaths | Eritrea | Both | 96.22 | 73.72 | 22.50 | 3.28 | 189.78 | 129.88 | 59.90 | 2.17 | 97.24 |
| Deaths | Estonia | Both | 768.81 | 643.35 | 125.46 | 5.13 | 653.80 | 487.42 | 166.38 | 2.93 | -14.96 |
| Deaths | Ethiopia | Both | 2323.18 | 1994.31 | 328.88 | 6.06 | 3034.87 | 2329.61 | 705.26 | 3.30 | 30.63 |
| Deaths | Fiji | Both | 28.36 | 18.63 | 9.73 | 1.92 | 55.00 | 36.32 | 18.68 | 1.94 | 93.92 |
| Deaths | Finland | Both | 2044.13 | 1619.55 | 424.59 | 3.81 | 2446.43 | 1609.32 | 837.11 | 1.92 | 19.68 |
| Deaths | France | Both | 24723.78 | 21171.02 | 3552.77 | 5.96 | 37040.51 | 26471.80 | 10568.71 | 2.50 | 49.82 |
| Deaths | Gabon | Both | 82.77 | 63.92 | 18.85 | 3.39 | 138.23 | 105.22 | 33.01 | 3.19 | 67.01 |
| Deaths | Gambia | Both | 26.97 | 20.86 | 6.11 | 3.41 | 69.37 | 50.00 | 19.37 | 2.58 | 157.26 |
| Deaths | Georgia | Both | 1285.01 | 1050.68 | 234.33 | 4.48 | 1492.60 | 1264.37 | 228.24 | 5.54 | 16.16 |
| Deaths | Germany | Both | 39420.67 | 31170.72 | 8249.95 | 3.78 | 51025.46 | 33425.13 | 17600.34 | 1.90 | 29.44 |
| Deaths | Ghana | Both | 384.86 | 276.39 | 108.48 | 2.55 | 1015.48 | 723.15 | 292.32 | 2.47 | 163.85 |
| Deaths | Greece | Both | 5410.67 | 4600.65 | 810.01 | 5.68 | 7575.74 | 5928.25 | 1647.50 | 3.60 | 40.01 |
| Deaths | Greenland | Both | 36.00 | 21.84 | 14.16 | 1.54 | 49.81 | 31.86 | 17.95 | 1.77 | 38.36 |
| Deaths | Grenada | Both | 8.76 | 6.08 | 2.68 | 2.26 | 19.29 | 11.90 | 7.40 | 1.61 | 120.26 |
| Deaths | Grenadines | Both | 6.57 | 4.22 | 2.35 | 1.80 | 13.37 | 8.97 | 4.39 | 2.04 | 103.46 |
| Deaths | Guam | Both | 26.94 | 20.63 | 6.30 | 3.27 | 65.69 | 43.33 | 22.36 | 1.94 | 143.85 |
| Deaths | Guatemala | Both | 273.71 | 163.14 | 110.57 | 1.48 | 691.52 | 450.21 | 241.31 | 1.87 | 152.65 |
| Deaths | Guinea | Both | 293.16 | 221.08 | 72.08 | 3.07 | 513.23 | 406.04 | 107.18 | 3.79 | 75.06 |
| Deaths | Guinea-Bissau | Both | 51.86 | 42.15 | 9.71 | 4.34 | 67.86 | 50.35 | 17.50 | 2.88 | 30.86 |
| Deaths | Guyana | Both | 25.37 | 17.67 | 7.70 | 2.29 | 44.69 | 27.45 | 17.24 | 1.59 | 76.17 |
| Deaths | Haiti | Both | 522.39 | 403.29 | 119.10 | 3.39 | 810.71 | 580.28 | 230.43 | 2.52 | 55.19 |
| Deaths | Honduras | Both | 157.91 | 109.40 | 48.51 | 2.26 | 468.36 | 300.86 | 167.50 | 1.80 | 196.60 |
| Deaths | Hungary | Both | 6908.17 | 5431.15 | 1477.02 | 3.68 | 8470.56 | 5407.63 | 3062.93 | 1.77 | 22.62 |
| Deaths | Iceland | Both | 94.31 | 52.77 | 41.54 | 1.27 | 143.18 | 78.92 | 64.25 | 1.23 | 51.82 |
| Deaths | India | Both | 33331.61 | 26753.52 | 6578.09 | 4.07 | 83891.46 | 59776.48 | 24114.98 | 2.48 | 151.69 |
| Deaths | Indonesia | Both | 15739.44 | 11511.67 | 4227.77 | 2.72 | 37221.42 | 28076.51 | 9144.91 | 3.07 | 136.49 |
| Deaths | Iran | Both | 2561.22 | 1956.68 | 604.54 | 3.24 | 7461.28 | 5184.58 | 2276.69 | 2.28 | 191.32 |
| Deaths | Iraq | Both | 1331.13 | 1067.37 | 263.77 | 4.05 | 2107.01 | 1501.71 | 605.30 | 2.48 | 58.29 |
| Deaths | Ireland | Both | 1606.74 | 1086.62 | 520.12 | 2.09 | 2024.67 | 1125.87 | 898.79 | 1.25 | 26.01 |
| Deaths | Israel | Both | 1033.91 | 705.77 | 328.14 | 2.15 | 2191.45 | 1429.41 | 762.04 | 1.88 | 111.96 |
| Deaths | Italy | Both | 32063.55 | 26607.72 | 5455.83 | 4.88 | 34098.94 | 24437.82 | 9661.11 | 2.53 | 6.35 |
| Deaths | Ivory Coast | Both | 281.34 | 218.65 | 62.69 | 3.49 | 603.00 | 457.10 | 145.90 | 3.13 | 114.33 |
| Deaths | Jamaica | Both | 259.91 | 200.89 | 59.01 | 3.40 | 557.13 | 444.93 | 112.19 | 3.97 | 114.36 |
| Deaths | Japan | Both | 38304.49 | 27844.22 | 10460.27 | 2.66 | 79862.29 | 55844.86 | 24017.44 | 2.33 | 108.49 |
| Deaths | Jordan | Both | 179.27 | 150.64 | 28.62 | 5.26 | 682.24 | 535.59 | 146.65 | 3.65 | 280.57 |
| Deaths | Kazakhstan | Both | 4919.18 | 4037.21 | 881.97 | 4.58 | 3236.54 | 2597.54 | 639.00 | 4.06 | -34.21 |
| Deaths | Kenya | Both | 517.92 | 385.33 | 132.59 | 2.91 | 1195.61 | 902.08 | 293.53 | 3.07 | 130.85 |
| Deaths | Kiribati | Both | 5.83 | 3.73 | 2.11 | 1.77 | 10.32 | 6.65 | 3.68 | 1.81 | 77.02 |
| Deaths | Kuwait | Both | 63.53 | 49.76 | 13.77 | 3.61 | 154.61 | 123.76 | 30.85 | 4.01 | 143.36 |
| Deaths | Kyrgyzstan | Both | 720.14 | 584.90 | 135.24 | 4.33 | 485.91 | 367.01 | 118.90 | 3.09 | -32.53 |
| Deaths | Laos | Both | 548.28 | 415.67 | 132.61 | 3.13 | 863.36 | 617.63 | 245.73 | 2.51 | 57.47 |
| Deaths | Latvia | Both | 1226.48 | 1021.89 | 204.60 | 4.99 | 965.88 | 757.87 | 208.00 | 3.64 | -21.25 |
| Deaths | Lebanon | Both | 588.63 | 460.13 | 128.50 | 3.58 | 1315.92 | 768.09 | 547.83 | 1.40 | 123.55 |
| Deaths | Lesotho | Both | 130.89 | 105.37 | 25.52 | 4.13 | 193.41 | 148.75 | 44.66 | 3.33 | 47.76 |
| Deaths | Liberia | Both | 99.15 | 78.26 | 20.89 | 3.75 | 133.15 | 98.63 | 34.52 | 2.86 | 34.30 |
| Deaths | Libya | Both | 411.08 | 373.33 | 37.74 | 9.89 | 928.60 | 815.97 | 112.63 | 7.24 | 125.89 |
| Deaths | Lithuania | Both | 1500.07 | 1283.88 | 216.19 | 5.94 | 1395.62 | 1124.67 | 270.95 | 4.15 | -6.96 |
| Deaths | Luxembourg | Both | 213.27 | 176.13 | 37.13 | 4.74 | 283.09 | 186.20 | 96.89 | 1.92 | 32.74 |
| Deaths | Macedonia | Both | 457.89 | 389.98 | 67.91 | 5.74 | 1015.96 | 849.16 | 166.80 | 5.09 | 121.88 |
| Deaths | Madagascar | Both | 413.24 | 310.04 | 103.19 | 3.00 | 672.71 | 495.83 | 176.88 | 2.80 | 62.79 |
| Deaths | Malawi | Both | 244.06 | 173.78 | 70.28 | 2.47 | 401.77 | 285.82 | 115.94 | 2.47 | 64.62 |
| Deaths | Malaysia | Both | 1690.24 | 1278.05 | 412.18 | 3.10 | 4709.85 | 3396.12 | 1313.73 | 2.59 | 178.65 |
| Deaths | Maldives | Both | 11.56 | 10.28 | 1.28 | 8.03 | 19.22 | 15.74 | 3.49 | 4.51 | 66.29 |
| Deaths | Mali | Both | 283.01 | 207.57 | 75.44 | 2.75 | 534.58 | 387.03 | 147.55 | 2.62 | 88.89 |
| Deaths | Malta | Both | 108.49 | 94.09 | 14.40 | 6.53 | 199.23 | 159.41 | 39.82 | 4.00 | 83.63 |
| Deaths | Marshall Islands | Both | 4.19 | 3.51 | 0.67 | 5.24 | 8.27 | 6.71 | 1.56 | 4.29 | 97.63 |
| Deaths | Mauritania | Both | 104.77 | 73.66 | 31.11 | 2.37 | 180.96 | 125.41 | 55.55 | 2.26 | 72.72 |
| Deaths | Mauritius | Both | 97.05 | 74.52 | 22.53 | 3.31 | 189.12 | 136.80 | 52.32 | 2.61 | 94.87 |
| Deaths | Mexico | Both | 5662.05 | 3934.29 | 1727.77 | 2.28 | 9537.94 | 6356.02 | 3181.92 | 2.00 | 68.45 |
| Deaths | Micronesia | Both | 11.98 | 8.53 | 3.45 | 2.47 | 15.79 | 11.16 | 4.63 | 2.41 | 31.82 |
| Deaths | Moldova | Both | 1198.54 | 981.18 | 217.36 | 4.51 | 1058.01 | 855.27 | 202.73 | 4.22 | -11.73 |
| Deaths | Mongolia | Both | 389.10 | 289.95 | 99.15 | 2.92 | 505.96 | 390.67 | 115.29 | 3.39 | 30.03 |
| Deaths | Montenegro | Both | 267.75 | 207.51 | 60.24 | 3.44 | 444.03 | 331.44 | 112.59 | 2.94 | 65.84 |
| Deaths | Morocco | Both | 1943.65 | 1714.37 | 229.29 | 7.48 | 4431.58 | 3878.44 | 553.14 | 7.01 | 128.00 |
| Deaths | Mozambique | Both | 515.30 | 413.03 | 102.27 | 4.04 | 997.42 | 806.14 | 191.28 | 4.21 | 93.56 |
| Deaths | Myanmar | Both | 5394.13 | 3747.77 | 1646.36 | 2.28 | 8178.85 | 5285.33 | 2893.52 | 1.83 | 51.62 |
| Deaths | Namibia | Both | 64.54 | 46.22 | 18.32 | 2.52 | 100.67 | 69.22 | 31.45 | 2.20 | 55.99 |
| Deaths | Nepal | Both | 1072.66 | 709.90 | 362.76 | 1.96 | 2327.97 | 1391.89 | 936.09 | 1.49 | 117.03 |
| Deaths | Netherlands | Both | 9356.16 | 7927.75 | 1428.41 | 5.55 | 12200.38 | 7281.67 | 4918.71 | 1.48 | 30.40 |
| Deaths | New Zealand | Both | 1336.21 | 900.64 | 435.57 | 2.07 | 1809.34 | 964.52 | 844.82 | 1.14 | 35.41 |
| Deaths | Nicaragua | Both | 106.11 | 71.50 | 34.61 | 2.07 | 273.71 | 172.00 | 101.72 | 1.69 | 157.95 |
| Deaths | Niger | Both | 228.53 | 173.02 | 55.51 | 3.12 | 544.03 | 394.02 | 150.01 | 2.63 | 138.06 |
| Deaths | Nigeria | Both | 3678.98 | 2857.90 | 821.08 | 3.48 | 6093.71 | 4251.71 | 1842.00 | 2.31 | 65.64 |
| Deaths | North Korea | Both | 3903.10 | 2630.16 | 1272.94 | 2.07 | 7488.55 | 5363.77 | 2124.78 | 2.52 | 91.86 |
| Deaths | Northern Mariana Islands | Both | 6.04 | 4.87 | 1.17 | 4.15 | 14.70 | 11.25 | 3.45 | 3.27 | 143.40 |
| Deaths | Norway | Both | 1624.62 | 1158.96 | 465.67 | 2.49 | 2112.36 | 1200.05 | 912.31 | 1.32 | 30.02 |
| Deaths | Oman | Both | 53.26 | 43.62 | 9.64 | 4.53 | 116.15 | 88.70 | 27.46 | 3.23 | 118.09 |
| Deaths | Pakistan | Both | 7021.99 | 5749.40 | 1272.60 | 4.52 | 15029.49 | 11880.52 | 3148.97 | 3.77 | 114.03 |
| Deaths | Palestine | Both | 152.54 | 120.14 | 32.39 | 3.71 | 348.98 | 255.72 | 93.26 | 2.74 | 128.78 |
| Deaths | Panama | Both | 197.52 | 151.88 | 45.64 | 3.33 | 377.99 | 247.40 | 130.59 | 1.89 | 91.37 |
| Deaths | Papua New Guinea | Both | 457.02 | 355.36 | 101.66 | 3.50 | 1118.99 | 865.78 | 253.21 | 3.42 | 144.84 |
| Deaths | Paraguay | Both | 244.03 | 169.17 | 74.85 | 2.26 | 765.17 | 570.15 | 195.02 | 2.92 | 213.56 |
| Deaths | Peru | Both | 1526.65 | 971.32 | 555.33 | 1.75 | 2905.81 | 1618.14 | 1287.67 | 1.26 | 90.34 |
| Deaths | Philippines | Both | 4977.53 | 3701.27 | 1276.26 | 2.90 | 12733.65 | 8931.52 | 3802.13 | 2.35 | 155.82 |
| Deaths | Poland | Both | 18694.20 | 15384.79 | 3309.40 | 4.65 | 27049.35 | 19007.28 | 8042.07 | 2.36 | 44.69 |
| Deaths | Portugal | Both | 2664.83 | 2139.32 | 525.51 | 4.07 | 4397.89 | 3317.83 | 1080.06 | 3.07 | 65.03 |
| Deaths | Puerto Rico | Both | 572.39 | 394.93 | 177.46 | 2.23 | 775.37 | 478.38 | 297.00 | 1.61 | 35.46 |
| Deaths | Qatar | Both | 18.73 | 16.17 | 2.55 | 6.33 | 90.97 | 77.79 | 13.18 | 5.90 | 385.84 |
| Deaths | Republic of Congo | Both | 164.26 | 121.64 | 42.62 | 2.85 | 289.07 | 197.26 | 91.81 | 2.15 | 75.99 |
| Deaths | Romania | Both | 6825.06 | 5683.57 | 1141.49 | 4.98 | 10500.50 | 8176.11 | 2324.39 | 3.52 | 53.85 |
| Deaths | Russia | Both | 60306.56 | 50451.92 | 9854.64 | 5.12 | 50317.25 | 40935.90 | 9381.35 | 4.36 | -16.56 |
| Deaths | Rwanda | Both | 274.67 | 213.19 | 61.47 | 3.47 | 376.15 | 261.39 | 114.77 | 2.28 | 36.95 |
| Deaths | Saint Lucia | Both | 11.12 | 7.59 | 3.52 | 2.15 | 22.78 | 14.61 | 8.17 | 1.79 | 104.91 |
| Deaths | Saint Vincent | Both | 6.57 | 4.22 | 2.35 | 1.80 | 13.37 | 8.97 | 4.39 | 2.04 | 103.46 |
| Deaths | Samoa | Both | 6.56 | 4.87 | 1.69 | 2.88 | 10.12 | 7.10 | 3.02 | 2.35 | 54.32 |
| Deaths | Sao Tome and Principe | Both | 8.14 | 6.27 | 1.87 | 3.35 | 14.64 | 11.21 | 3.44 | 3.26 | 79.88 |
| Deaths | Saudi Arabia | Both | 472.28 | 410.20 | 62.07 | 6.61 | 1263.46 | 1040.62 | 222.84 | 4.67 | 167.53 |
| Deaths | Senegal | Both | 378.04 | 302.14 | 75.90 | 3.98 | 770.21 | 589.69 | 180.51 | 3.27 | 103.74 |
| Deaths | Serbia | Both | 4807.28 | 3928.84 | 878.44 | 4.47 | 6504.60 | 4630.25 | 1874.35 | 2.47 | 35.31 |
| Deaths | Seychelles | Both | 8.94 | 7.08 | 1.86 | 3.80 | 15.88 | 12.02 | 3.87 | 3.11 | 77.60 |
| Deaths | Sierra Leone | Both | 203.85 | 157.79 | 46.06 | 3.43 | 321.24 | 239.96 | 81.28 | 2.95 | 57.59 |
| Deaths | Singapore | Both | 802.63 | 578.14 | 224.49 | 2.58 | 1231.87 | 848.09 | 383.77 | 2.21 | 53.48 |
| Deaths | Slovakia | Both | 2258.47 | 1977.71 | 280.75 | 7.04 | 2343.41 | 1764.25 | 579.15 | 3.05 | 3.76 |
| Deaths | Slovenia | Both | 862.19 | 708.07 | 154.13 | 4.59 | 1146.17 | 814.12 | 332.05 | 2.45 | 32.94 |
| Deaths | Solomon Islands | Both | 31.69 | 25.51 | 6.17 | 4.13 | 62.77 | 47.14 | 15.63 | 3.02 | 98.10 |
| Deaths | Somalia | Both | 204.52 | 155.16 | 49.36 | 3.14 | 464.85 | 351.84 | 113.01 | 3.11 | 127.29 |
| Deaths | South Africa | Both | 3831.32 | 2651.93 | 1179.39 | 2.25 | 7125.78 | 4860.48 | 2265.30 | 2.15 | 85.99 |
| Deaths | South Korea | Both | 6325.53 | 4723.14 | 1602.38 | 2.95 | 19444.42 | 13954.15 | 5490.27 | 2.54 | 207.40 |
| Deaths | South Sudan | Both | 238.71 | 188.94 | 49.76 | 3.80 | 340.59 | 269.15 | 71.44 | 3.77 | 42.68 |
| Deaths | Spain | Both | 15518.12 | 13824.26 | 1693.86 | 8.16 | 22566.03 | 17835.66 | 4730.38 | 3.77 | 45.42 |
| Deaths | Sri Lanka | Both | 805.45 | 610.09 | 195.36 | 3.12 | 1734.33 | 1254.34 | 479.99 | 2.61 | 115.33 |
| Deaths | Sudan | Both | 802.11 | 626.97 | 175.14 | 3.58 | 1405.04 | 1073.06 | 331.98 | 3.23 | 75.17 |
| Deaths | Suriname | Both | 31.75 | 21.83 | 9.92 | 2.20 | 82.78 | 53.35 | 29.43 | 1.81 | 160.74 |
| Deaths | Swaziland | Both | 50.27 | 39.00 | 11.27 | 3.46 | 86.75 | 66.28 | 20.47 | 3.24 | 72.57 |
| Deaths | Sweden | Both | 2931.94 | 1955.84 | 976.11 | 2.00 | 3961.16 | 1960.84 | 2000.32 | 0.98 | 35.10 |
| Deaths | Switzerland | Both | 3029.46 | 2428.92 | 600.54 | 4.04 | 3420.76 | 2103.24 | 1317.52 | 1.60 | 12.92 |
| Deaths | Syria | Both | 501.27 | 387.89 | 113.38 | 3.42 | 1206.78 | 939.55 | 267.23 | 3.52 | 140.75 |
| Deaths | Taiwan (Province of China) | Both | 3654.66 | 2601.72 | 1052.94 | 2.47 | 10546.87 | 6744.30 | 3802.57 | 1.77 | 188.59 |
| Deaths | Tajikistan | Both | 370.46 | 265.44 | 105.02 | 2.53 | 390.45 | 265.70 | 124.75 | 2.13 | 5.40 |
| Deaths | Tanzania | Both | 784.89 | 587.05 | 197.84 | 2.97 | 1613.28 | 1165.42 | 447.86 | 2.60 | 105.54 |
| Deaths | Thailand | Both | 10772.93 | 6601.70 | 4171.23 | 1.58 | 19475.65 | 13105.12 | 6370.53 | 2.06 | 80.78 |
| Deaths | Timor-Leste | Both | 43.88 | 31.28 | 12.60 | 2.48 | 145.96 | 108.32 | 37.64 | 2.88 | 232.67 |
| Deaths | Tobago | Both | 96.45 | 73.20 | 23.24 | 3.15 | 179.12 | 131.34 | 47.77 | 2.75 | 85.72 |
| Deaths | Togo | Both | 109.33 | 79.88 | 29.44 | 2.71 | 286.26 | 206.32 | 79.94 | 2.58 | 161.83 |
| Deaths | Tonga | Both | 15.57 | 12.09 | 3.47 | 3.48 | 21.10 | 16.22 | 4.88 | 3.32 | 35.55 |
| Deaths | Trinidad | Both | 96.45 | 73.20 | 23.24 | 3.15 | 179.12 | 131.34 | 47.77 | 2.75 | 85.72 |
| Deaths | Tunisia | Both | 889.64 | 823.47 | 66.17 | 12.44 | 2097.59 | 1875.66 | 221.93 | 8.45 | 135.78 |
| Deaths | Turkey | Both | 14303.85 | 12466.02 | 1837.84 | 6.78 | 27340.91 | 23590.83 | 3750.08 | 6.29 | 91.14 |
| Deaths | Turkmenistan | Both | 276.73 | 217.51 | 59.22 | 3.67 | 382.36 | 287.10 | 95.26 | 3.01 | 38.17 |
| Deaths | Uganda | Both | 352.79 | 233.06 | 119.73 | 1.95 | 817.51 | 555.13 | 262.38 | 2.12 | 131.72 |
| Deaths | UK | Both | 41511.08 | 27941.09 | 13569.99 | 2.06 | 39383.39 | 21533.26 | 17850.13 | 1.21 | -5.13 |
| Deaths | Ukraine | Both | 23911.61 | 19669.19 | 4242.43 | 4.64 | 15686.47 | 13029.54 | 2656.93 | 4.90 | -34.40 |
| Deaths | United Arab Emirates | Both | 51.58 | 42.71 | 8.87 | 4.81 | 435.20 | 372.72 | 62.48 | 5.97 | 743.79 |
| Deaths | Uruguay | Both | 1472.60 | 1277.19 | 195.41 | 6.54 | 1571.32 | 1151.32 | 420.00 | 2.74 | 6.70 |
| Deaths | USA | Both | 152628.98 | 97045.38 | 55583.60 | 1.75 | 190697.62 | 105766.07 | 84931.54 | 1.25 | 24.94 |
| Deaths | Uzbekistan | Both | 1689.75 | 1253.35 | 436.40 | 2.87 | 2236.11 | 1675.77 | 560.34 | 2.99 | 32.33 |
| Deaths | Vanuatu | Both | 16.44 | 12.80 | 3.64 | 3.52 | 40.07 | 30.50 | 9.58 | 3.18 | 143.71 |
| Deaths | Venezuela | Both | 1649.10 | 1065.63 | 583.47 | 1.83 | 4705.04 | 2899.91 | 1805.13 | 1.61 | 185.31 |
| Deaths | Vietnam | Both | 14319.37 | 10021.68 | 4297.68 | 2.33 | 35554.19 | 24666.32 | 10887.88 | 2.27 | 148.29 |
| Deaths | Virgin Islands | Both | 15.44 | 10.81 | 4.63 | 2.33 | 40.84 | 27.77 | 13.06 | 2.13 | 164.48 |
| Deaths | Yemen | Both | 528.06 | 407.25 | 120.81 | 3.37 | 1262.70 | 973.96 | 288.74 | 3.37 | 139.12 |
| Deaths | Zambia | Both | 260.49 | 206.25 | 54.24 | 3.80 | 475.69 | 357.69 | 117.99 | 3.03 | 82.61 |
| Deaths | Zimbabwe | Both | 504.59 | 394.94 | 109.65 | 3.60 | 705.33 | 474.34 | 230.99 | 2.05 | 39.78 |
| Incidence | Afghanistan | Both | 943.43 | 764.15 | 179.28 | 4.26 | 1226.91 | 899.76 | 327.15 | 2.75 | 30.05 |
| Incidence | Albania | Both | 532.90 | 450.81 | 82.09 | 5.49 | 986.09 | 821.77 | 164.32 | 5.00 | 85.04 |
| Incidence | Algeria | Both | 1261.94 | 1058.01 | 203.93 | 5.19 | 2664.83 | 2141.25 | 523.58 | 4.09 | 111.17 |
| Incidence | American Samoa | Both | 5.82 | 4.57 | 1.25 | 3.65 | 9.84 | 6.99 | 2.85 | 2.46 | 69.05 |
| Incidence | Andorra | Both | 19.96 | 15.76 | 4.20 | 3.75 | 40.53 | 27.33 | 13.20 | 2.07 | 103.09 |
| Incidence | Angola | Both | 562.99 | 452.76 | 110.23 | 4.11 | 1129.67 | 843.90 | 285.78 | 2.95 | 100.66 |
| Incidence | Antigua | Both | 4.22 | 3.11 | 1.11 | 2.81 | 7.61 | 4.64 | 2.96 | 1.57 | 80.35 |
| Incidence | Argentina | Both | 8381.68 | 6816.23 | 1565.45 | 4.35 | 10717.01 | 7247.10 | 3469.90 | 2.09 | 27.86 |
| Incidence | Armenia | Both | 874.62 | 738.21 | 136.40 | 5.41 | 1191.38 | 989.20 | 202.18 | 4.89 | 36.22 |
| Incidence | Australia | Both | 7096.24 | 5182.91 | 1913.32 | 2.71 | 12966.15 | 7801.27 | 5164.88 | 1.51 | 82.72 |
| Incidence | Austria | Both | 3310.78 | 2500.23 | 810.56 | 3.08 | 5431.33 | 3261.07 | 2170.26 | 1.50 | 64.05 |
| Incidence | Azerbaijan | Both | 1051.56 | 863.88 | 187.68 | 4.60 | 1937.98 | 1563.03 | 374.95 | 4.17 | 84.30 |
| Incidence | Bahamas | Both | 24.04 | 18.30 | 5.74 | 3.19 | 48.71 | 33.34 | 15.37 | 2.17 | 102.63 |
| Incidence | Bahrain | Both | 54.62 | 43.64 | 10.98 | 3.98 | 88.75 | 67.94 | 20.81 | 3.26 | 62.50 |
| Incidence | Bangladesh | Both | 10707.11 | 8517.09 | 2190.02 | 3.89 | 24190.54 | 18471.42 | 5719.12 | 3.23 | 125.93 |
| Incidence | Barbados | Both | 25.59 | 18.30 | 7.29 | 2.51 | 37.76 | 24.13 | 13.63 | 1.77 | 47.55 |
| Incidence | Barbuda | Both | 4.22 | 3.11 | 1.11 | 2.81 | 7.61 | 4.64 | 2.96 | 1.57 | 80.35 |
| Incidence | Belarus | Both | 3949.25 | 3433.37 | 515.89 | 6.66 | 3575.20 | 3025.75 | 549.45 | 5.51 | -9.47 |
| Incidence | Belgium | Both | 7026.43 | 6043.52 | 982.91 | 6.15 | 8027.74 | 5606.91 | 2420.83 | 2.32 | 14.25 |
| Incidence | Belize | Both | 9.03 | 6.72 | 2.30 | 2.92 | 30.73 | 22.03 | 8.69 | 2.53 | 240.45 |
| Incidence | Benin | Both | 192.07 | 144.17 | 47.90 | 3.01 | 390.48 | 281.55 | 108.93 | 2.58 | 103.30 |
| Incidence | Bermuda | Both | 18.85 | 13.57 | 5.28 | 2.57 | 27.00 | 19.11 | 7.90 | 2.42 | 43.28 |
| Incidence | Bhutan | Both | 20.58 | 14.73 | 5.85 | 2.52 | 42.32 | 29.60 | 12.72 | 2.33 | 105.63 |
| Incidence | Bolivia | Both | 393.89 | 270.78 | 123.10 | 2.20 | 937.82 | 566.10 | 371.72 | 1.52 | 138.09 |
| Incidence | Bosnia and Herzegovina | Both | 1492.55 | 1258.60 | 233.96 | 5.38 | 2147.76 | 1692.18 | 455.58 | 3.71 | 43.90 |
| Incidence | Botswana | Both | 89.45 | 67.28 | 22.16 | 3.04 | 149.39 | 94.01 | 55.38 | 1.70 | 67.02 |
| Incidence | Brazil | Both | 13795.33 | 9496.98 | 4298.35 | 2.21 | 30929.99 | 17926.78 | 13003.21 | 1.38 | 124.21 |
| Incidence | Brunei | Both | 34.12 | 21.57 | 12.56 | 1.72 | 91.49 | 50.66 | 40.84 | 1.24 | 168.13 |
| Incidence | Bulgaria | Both | 3144.29 | 2691.51 | 452.78 | 5.94 | 3865.21 | 3088.64 | 776.57 | 3.98 | 22.93 |
| Incidence | Burkina Faso | Both | 332.77 | 231.98 | 100.79 | 2.30 | 619.54 | 430.61 | 188.93 | 2.28 | 86.18 |
| Incidence | Burundi | Both | 194.97 | 148.30 | 46.67 | 3.18 | 259.80 | 203.16 | 56.65 | 3.59 | 33.26 |
| Incidence | Cambodia | Both | 1101.02 | 816.23 | 284.79 | 2.87 | 2189.37 | 1590.18 | 599.18 | 2.65 | 98.85 |
| Incidence | Cameroon | Both | 452.06 | 342.51 | 109.55 | 3.13 | 1126.79 | 846.33 | 280.46 | 3.02 | 149.26 |
| Incidence | Canada | Both | 15126.47 | 10182.41 | 4944.07 | 2.06 | 25484.28 | 13673.64 | 11810.64 | 1.16 | 68.47 |
| Incidence | Cape Verde | Both | 18.96 | 11.15 | 7.80 | 1.43 | 45.69 | 28.08 | 17.60 | 1.60 | 141.01 |
| Incidence | Central African Republic | Both | 147.75 | 115.99 | 31.76 | 3.65 | 227.31 | 180.57 | 46.75 | 3.86 | 53.85 |
| Incidence | Chad | Both | 237.62 | 170.50 | 67.12 | 2.54 | 567.40 | 435.28 | 132.12 | 3.29 | 138.78 |
| Incidence | Chile | Both | 1703.06 | 1220.09 | 482.97 | 2.53 | 3429.37 | 1990.54 | 1438.83 | 1.38 | 101.37 |
| Incidence | China | Both | 241036.12 | 165389.47 | 75646.65 | 2.19 | 813209.20 | 564092.02 | 249117.19 | 2.26 | 237.38 |
| Incidence | Colombia | Both | 2337.74 | 1519.93 | 817.81 | 1.86 | 5214.89 | 3009.89 | 2204.99 | 1.37 | 123.07 |
| Incidence | Comoros | Both | 20.65 | 14.41 | 6.23 | 2.31 | 33.47 | 22.40 | 11.06 | 2.02 | 62.08 |
| Incidence | Costa Rica | Both | 186.98 | 135.51 | 51.47 | 2.63 | 443.15 | 312.50 | 130.65 | 2.39 | 137.00 |
| Incidence | Croatia | Both | 2689.75 | 2277.22 | 412.53 | 5.52 | 3282.40 | 2407.79 | 874.61 | 2.75 | 22.03 |
| Incidence | Cuba | Both | 3109.83 | 2247.64 | 862.19 | 2.61 | 5717.13 | 3657.41 | 2059.73 | 1.78 | 83.84 |
| Incidence | Cyprus | Both | 166.57 | 136.52 | 30.05 | 4.54 | 499.71 | 414.46 | 85.25 | 4.86 | 200.00 |
| Incidence | Czech Republic | Both | 6353.35 | 5427.90 | 925.45 | 5.87 | 7009.50 | 4781.24 | 2228.26 | 2.15 | 10.33 |
| Incidence | Democratic Republic of the Congo | Both | 1539.19 | 1138.19 | 401.00 | 2.84 | 2702.77 | 1959.93 | 742.83 | 2.64 | 75.60 |
| Incidence | Denmark | Both | 3591.96 | 2315.20 | 1276.75 | 1.81 | 4991.98 | 2582.58 | 2409.40 | 1.07 | 38.98 |
| Incidence | Djibouti | Both | 14.10 | 10.00 | 4.10 | 2.44 | 49.49 | 37.87 | 11.62 | 3.26 | 251.05 |
| Incidence | Dominica | Both | 8.46 | 6.06 | 2.40 | 2.52 | 12.13 | 8.29 | 3.84 | 2.16 | 43.45 |
| Incidence | Dominican Republic | Both | 356.51 | 230.18 | 126.34 | 1.82 | 1122.66 | 706.98 | 415.68 | 1.70 | 214.90 |
| Incidence | Ecuador | Both | 408.51 | 275.65 | 132.86 | 2.07 | 1198.28 | 676.67 | 521.61 | 1.30 | 193.33 |
| Incidence | Egypt | Both | 1691.54 | 1240.34 | 451.20 | 2.75 | 4319.96 | 3155.56 | 1164.40 | 2.71 | 155.39 |
| Incidence | El Salvador | Both | 195.96 | 122.95 | 73.01 | 1.68 | 494.46 | 287.10 | 207.36 | 1.38 | 152.32 |
| Incidence | Equatorial Guinea | Both | 22.72 | 18.04 | 4.68 | 3.85 | 45.98 | 29.51 | 16.48 | 1.79 | 102.39 |
| Incidence | Eritrea | Both | 96.08 | 73.60 | 22.48 | 3.27 | 187.99 | 128.94 | 59.05 | 2.18 | 95.66 |
| Incidence | Estonia | Both | 766.38 | 637.26 | 129.12 | 4.94 | 714.99 | 478.01 | 236.98 | 2.02 | -6.71 |
| Incidence | Ethiopia | Both | 2288.77 | 1958.24 | 330.52 | 5.92 | 2893.79 | 2188.93 | 704.86 | 3.11 | 26.43 |
| Incidence | Fiji | Both | 28.04 | 18.39 | 9.65 | 1.91 | 53.56 | 35.39 | 18.18 | 1.95 | 91.00 |
| Incidence | Finland | Both | 2356.80 | 1817.09 | 539.72 | 3.37 | 3106.59 | 2023.33 | 1083.26 | 1.87 | 31.81 |
| Incidence | France | Both | 24105.94 | 20783.29 | 3322.65 | 6.26 | 40694.54 | 28928.83 | 11765.70 | 2.46 | 68.82 |
| Incidence | Gabon | Both | 80.43 | 62.22 | 18.21 | 3.42 | 134.47 | 102.56 | 31.91 | 3.21 | 67.20 |
| Incidence | Gambia | Both | 26.42 | 20.50 | 5.92 | 3.46 | 66.26 | 47.90 | 18.35 | 2.61 | 150.77 |
| Incidence | Georgia | Both | 1304.04 | 1073.25 | 230.79 | 4.65 | 1467.72 | 1251.20 | 216.52 | 5.78 | 12.55 |
| Incidence | Germany | Both | 44699.14 | 35916.40 | 8782.74 | 4.09 | 65919.46 | 42659.02 | 23260.44 | 1.83 | 47.47 |
| Incidence | Ghana | Both | 380.70 | 272.59 | 108.11 | 2.52 | 996.80 | 707.96 | 288.84 | 2.45 | 161.84 |
| Incidence | Greece | Both | 5251.33 | 4474.70 | 776.63 | 5.76 | 7873.82 | 6138.10 | 1735.72 | 3.54 | 49.94 |
| Incidence | Greenland | Both | 35.26 | 21.50 | 13.75 | 1.56 | 47.79 | 30.60 | 17.19 | 1.78 | 35.55 |
| Incidence | Grenada | Both | 8.23 | 5.73 | 2.50 | 2.30 | 17.94 | 11.24 | 6.71 | 1.68 | 118.00 |
| Incidence | Grenadines | Both | 6.21 | 4.01 | 2.20 | 1.82 | 12.77 | 8.58 | 4.19 | 2.04 | 105.62 |
| Incidence | Guam | Both | 26.52 | 20.39 | 6.13 | 3.33 | 63.74 | 42.32 | 21.42 | 1.98 | 140.36 |
| Incidence | Guatemala | Both | 271.06 | 159.81 | 111.25 | 1.44 | 660.61 | 426.94 | 233.67 | 1.83 | 143.71 |
| Incidence | Guinea | Both | 283.92 | 214.21 | 69.71 | 3.07 | 501.47 | 398.00 | 103.47 | 3.85 | 76.62 |
| Incidence | Guinea-Bissau | Both | 51.37 | 41.56 | 9.81 | 4.24 | 67.66 | 50.08 | 17.58 | 2.85 | 31.72 |
| Incidence | Guyana | Both | 24.95 | 17.33 | 7.62 | 2.27 | 43.89 | 26.85 | 17.05 | 1.58 | 75.93 |
| Incidence | Haiti | Both | 518.40 | 399.40 | 118.99 | 3.36 | 792.09 | 564.50 | 227.59 | 2.48 | 52.80 |
| Incidence | Honduras | Both | 156.11 | 107.46 | 48.65 | 2.21 | 454.40 | 286.35 | 168.06 | 1.70 | 191.07 |
| Incidence | Hungary | Both | 6826.01 | 5396.61 | 1429.40 | 3.78 | 8335.95 | 5308.70 | 3027.25 | 1.75 | 22.12 |
| Incidence | Iceland | Both | 96.53 | 54.42 | 42.11 | 1.29 | 177.71 | 96.52 | 81.19 | 1.19 | 84.10 |
| Incidence | India | Both | 33199.32 | 26601.94 | 6597.38 | 4.03 | 81179.74 | 57725.02 | 23454.72 | 2.46 | 144.52 |
| Incidence | Indonesia | Both | 15817.21 | 11518.29 | 4298.93 | 2.68 | 36174.47 | 27282.41 | 8892.06 | 3.07 | 128.70 |
| Incidence | Iran | Both | 2557.05 | 1944.07 | 612.98 | 3.17 | 7223.74 | 4999.80 | 2223.94 | 2.25 | 182.50 |
| Incidence | Iraq | Both | 1292.01 | 1031.92 | 260.09 | 3.97 | 2050.52 | 1452.83 | 597.69 | 2.43 | 58.71 |
| Incidence | Ireland | Both | 1520.61 | 1033.15 | 487.46 | 2.12 | 2319.89 | 1273.86 | 1046.03 | 1.22 | 52.56 |
| Incidence | Israel | Both | 975.65 | 670.39 | 305.26 | 2.20 | 2148.29 | 1413.04 | 735.25 | 1.92 | 120.19 |
| Incidence | Italy | Both | 31908.69 | 26615.56 | 5293.13 | 5.03 | 40976.60 | 28651.34 | 12325.26 | 2.32 | 28.42 |
| Incidence | Ivory Coast | Both | 284.50 | 222.03 | 62.47 | 3.55 | 599.61 | 456.47 | 143.14 | 3.19 | 110.76 |
| Incidence | Jamaica | Both | 251.70 | 195.69 | 56.02 | 3.49 | 550.02 | 442.70 | 107.32 | 4.13 | 118.52 |
| Incidence | Japan | Both | 47597.75 | 34433.04 | 13164.71 | 2.62 | 107264.93 | 75262.21 | 32002.72 | 2.35 | 125.36 |
| Incidence | Jordan | Both | 181.49 | 152.28 | 29.21 | 5.21 | 674.22 | 527.34 | 146.88 | 3.59 | 271.49 |
| Incidence | Kazakhstan | Both | 4994.72 | 4126.24 | 868.48 | 4.75 | 3218.88 | 2592.89 | 625.98 | 4.14 | -35.55 |
| Incidence | Kenya | Both | 494.12 | 364.54 | 129.58 | 2.81 | 1149.11 | 863.85 | 285.26 | 3.03 | 132.56 |
| Incidence | Kiribati | Both | 5.80 | 3.73 | 2.07 | 1.80 | 10.40 | 6.80 | 3.60 | 1.89 | 79.21 |
| Incidence | Kuwait | Both | 64.57 | 50.29 | 14.27 | 3.52 | 158.29 | 124.64 | 33.65 | 3.70 | 145.16 |
| Incidence | Kyrgyzstan | Both | 728.39 | 595.28 | 133.10 | 4.47 | 484.47 | 366.86 | 117.61 | 3.12 | -33.49 |
| Incidence | Laos | Both | 546.44 | 413.65 | 132.79 | 3.12 | 847.37 | 603.59 | 243.78 | 2.48 | 55.07 |
| Incidence | Latvia | Both | 1242.94 | 1038.98 | 203.95 | 5.09 | 1159.71 | 893.80 | 265.91 | 3.36 | -6.70 |
| Incidence | Lebanon | Both | 588.17 | 458.96 | 129.21 | 3.55 | 1418.72 | 822.17 | 596.55 | 1.38 | 141.21 |
| Incidence | Lesotho | Both | 128.28 | 103.71 | 24.56 | 4.22 | 191.27 | 148.50 | 42.77 | 3.47 | 49.11 |
| Incidence | Liberia | Both | 95.23 | 74.53 | 20.70 | 3.60 | 128.25 | 94.59 | 33.66 | 2.81 | 34.67 |
| Incidence | Libya | Both | 403.83 | 366.96 | 36.87 | 9.95 | 924.91 | 811.78 | 113.13 | 7.18 | 129.03 |
| Incidence | Lithuania | Both | 1520.83 | 1304.52 | 216.31 | 6.03 | 1657.71 | 1316.67 | 341.03 | 3.86 | 9.00 |
| Incidence | Luxembourg | Both | 211.29 | 175.21 | 36.08 | 4.86 | 342.53 | 222.90 | 119.63 | 1.86 | 62.11 |
| Incidence | Macedonia | Both | 462.04 | 393.00 | 69.04 | 5.69 | 1004.82 | 836.68 | 168.14 | 4.98 | 117.48 |
| Incidence | Madagascar | Both | 399.12 | 295.93 | 103.19 | 2.87 | 655.97 | 479.49 | 176.48 | 2.72 | 64.36 |
| Incidence | Malawi | Both | 231.80 | 164.31 | 67.49 | 2.43 | 379.38 | 269.70 | 109.68 | 2.46 | 63.67 |
| Incidence | Malaysia | Both | 1613.10 | 1218.14 | 394.96 | 3.08 | 4515.13 | 3261.15 | 1253.97 | 2.60 | 179.90 |
| Incidence | Maldives | Both | 11.48 | 10.15 | 1.32 | 7.69 | 18.56 | 15.11 | 3.44 | 4.39 | 61.73 |
| Incidence | Mali | Both | 277.58 | 202.87 | 74.71 | 2.72 | 516.20 | 371.97 | 144.23 | 2.58 | 85.97 |
| Incidence | Malta | Both | 107.94 | 94.11 | 13.83 | 6.80 | 237.35 | 194.89 | 42.46 | 4.59 | 119.89 |
| Incidence | Marshall Islands | Both | 4.17 | 3.50 | 0.66 | 5.28 | 8.33 | 6.74 | 1.59 | 4.24 | 99.96 |
| Incidence | Mauritania | Both | 102.48 | 72.21 | 30.28 | 2.38 | 173.64 | 119.22 | 54.42 | 2.19 | 69.43 |
| Incidence | Mauritius | Both | 94.55 | 72.49 | 22.07 | 3.28 | 181.97 | 131.42 | 50.55 | 2.60 | 92.46 |
| Incidence | Mexico | Both | 5398.76 | 3749.42 | 1649.34 | 2.27 | 9046.24 | 6035.38 | 3010.86 | 2.00 | 67.56 |
| Incidence | Micronesia | Both | 11.67 | 8.37 | 3.30 | 2.53 | 15.63 | 11.11 | 4.52 | 2.46 | 33.87 |
| Incidence | Moldova | Both | 1220.79 | 1003.53 | 217.25 | 4.62 | 1086.20 | 877.15 | 209.06 | 4.20 | -11.02 |
| Incidence | Mongolia | Both | 372.12 | 278.84 | 93.29 | 2.99 | 492.32 | 383.54 | 108.78 | 3.53 | 32.30 |
| Incidence | Montenegro | Both | 267.33 | 207.77 | 59.55 | 3.49 | 435.98 | 324.26 | 111.72 | 2.90 | 63.09 |
| Incidence | Morocco | Both | 1916.22 | 1693.40 | 222.83 | 7.60 | 4345.47 | 3813.93 | 531.54 | 7.18 | 126.77 |
| Incidence | Mozambique | Both | 493.78 | 393.04 | 100.73 | 3.90 | 965.08 | 781.04 | 184.04 | 4.24 | 95.45 |
| Incidence | Myanmar | Both | 5441.20 | 3789.60 | 1651.60 | 2.29 | 8044.38 | 5212.68 | 2831.70 | 1.84 | 47.84 |
| Incidence | Namibia | Both | 61.97 | 44.17 | 17.79 | 2.48 | 94.58 | 64.92 | 29.67 | 2.19 | 52.64 |
| Incidence | Nepal | Both | 1061.88 | 705.22 | 356.66 | 1.98 | 2214.73 | 1314.63 | 900.10 | 1.46 | 108.57 |
| Incidence | Netherlands | Both | 9335.30 | 7887.47 | 1447.83 | 5.45 | 14667.16 | 8484.55 | 6182.62 | 1.37 | 57.12 |
| Incidence | New Zealand | Both | 1407.86 | 950.30 | 457.56 | 2.08 | 2359.19 | 1264.24 | 1094.95 | 1.15 | 67.57 |
| Incidence | Nicaragua | Both | 103.62 | 69.41 | 34.21 | 2.03 | 260.08 | 163.01 | 97.07 | 1.68 | 151.00 |
| Incidence | Niger | Both | 225.44 | 169.94 | 55.50 | 3.06 | 532.65 | 384.47 | 148.18 | 2.59 | 136.27 |
| Incidence | Nigeria | Both | 3475.12 | 2708.65 | 766.47 | 3.53 | 5768.37 | 3978.04 | 1790.33 | 2.22 | 65.99 |
| Incidence | North Korea | Both | 3902.70 | 2642.36 | 1260.34 | 2.10 | 7293.66 | 5282.62 | 2011.04 | 2.63 | 86.89 |
| Incidence | Northern Mariana Islands | Both | 6.13 | 4.95 | 1.18 | 4.20 | 14.58 | 11.15 | 3.43 | 3.25 | 138.00 |
| Incidence | Norway | Both | 1844.74 | 1304.24 | 540.49 | 2.41 | 2827.93 | 1589.46 | 1238.47 | 1.28 | 53.30 |
| Incidence | Oman | Both | 52.58 | 43.03 | 9.55 | 4.50 | 118.79 | 90.22 | 28.57 | 3.16 | 125.91 |
| Incidence | Pakistan | Both | 6895.36 | 5625.46 | 1269.90 | 4.43 | 14975.41 | 11772.64 | 3202.77 | 3.68 | 117.18 |
| Incidence | Palestine | Both | 147.63 | 115.37 | 32.26 | 3.58 | 345.51 | 252.98 | 92.53 | 2.73 | 134.04 |
| Incidence | Panama | Both | 190.16 | 146.08 | 44.08 | 3.31 | 369.19 | 246.27 | 122.92 | 2.00 | 94.15 |
| Incidence | Papua New Guinea | Both | 476.10 | 370.24 | 105.86 | 3.50 | 1157.03 | 894.83 | 262.20 | 3.41 | 143.02 |
| Incidence | Paraguay | Both | 232.89 | 160.74 | 72.15 | 2.23 | 726.64 | 541.03 | 185.62 | 2.91 | 212.01 |
| Incidence | Peru | Both | 1473.66 | 931.98 | 541.68 | 1.72 | 2722.70 | 1510.30 | 1212.40 | 1.25 | 84.76 |
| Incidence | Philippines | Both | 5060.99 | 3766.85 | 1294.14 | 2.91 | 12551.82 | 8825.35 | 3726.47 | 2.37 | 148.01 |
| Incidence | Poland | Both | 18383.07 | 15201.33 | 3181.74 | 4.78 | 25692.34 | 17964.61 | 7727.73 | 2.32 | 39.76 |
| Incidence | Portugal | Both | 2541.14 | 2052.34 | 488.80 | 4.20 | 4201.52 | 3127.19 | 1074.33 | 2.91 | 65.34 |
| Incidence | Puerto Rico | Both | 538.60 | 372.99 | 165.60 | 2.25 | 767.69 | 482.71 | 284.98 | 1.69 | 42.54 |
| Incidence | Qatar | Both | 19.06 | 16.45 | 2.61 | 6.30 | 99.08 | 84.25 | 14.83 | 5.68 | 419.91 |
| Incidence | Republic of Congo | Both | 162.53 | 120.37 | 42.16 | 2.85 | 285.01 | 193.82 | 91.19 | 2.13 | 75.35 |
| Incidence | Romania | Both | 6967.17 | 5832.84 | 1134.33 | 5.14 | 10350.07 | 8097.75 | 2252.32 | 3.60 | 48.55 |
| Incidence | Russia | Both | 63832.81 | 53570.20 | 10262.61 | 5.22 | 61772.08 | 49625.95 | 12146.14 | 4.09 | -3.23 |
| Incidence | Rwanda | Both | 264.41 | 203.48 | 60.94 | 3.34 | 358.04 | 246.60 | 111.44 | 2.21 | 35.41 |
| Incidence | Saint Lucia | Both | 10.56 | 7.20 | 3.35 | 2.15 | 21.82 | 14.05 | 7.78 | 1.81 | 106.70 |
| Incidence | Saint Vincent | Both | 6.21 | 4.01 | 2.20 | 1.82 | 12.77 | 8.58 | 4.19 | 2.04 | 105.62 |
| Incidence | Samoa | Both | 6.45 | 4.79 | 1.66 | 2.89 | 9.72 | 6.83 | 2.90 | 2.36 | 50.73 |
| Incidence | Sao Tome and Principe | Both | 7.79 | 5.95 | 1.84 | 3.24 | 14.24 | 10.80 | 3.44 | 3.14 | 82.71 |
| Incidence | Saudi Arabia | Both | 458.19 | 397.46 | 60.73 | 6.54 | 1298.29 | 1052.43 | 245.86 | 4.28 | 183.35 |
| Incidence | Senegal | Both | 365.78 | 292.15 | 73.63 | 3.97 | 737.47 | 563.28 | 174.19 | 3.23 | 101.62 |
| Incidence | Serbia | Both | 4868.89 | 3972.51 | 896.39 | 4.43 | 6707.13 | 4668.21 | 2038.92 | 2.29 | 37.75 |
| Incidence | Seychelles | Both | 8.59 | 6.83 | 1.76 | 3.88 | 15.46 | 11.82 | 3.65 | 3.24 | 79.97 |
| Incidence | Sierra Leone | Both | 193.66 | 149.38 | 44.28 | 3.37 | 307.79 | 229.06 | 78.73 | 2.91 | 58.93 |
| Incidence | Singapore | Both | 801.96 | 578.31 | 223.65 | 2.59 | 1708.86 | 1160.32 | 548.54 | 2.12 | 113.09 |
| Incidence | Slovakia | Both | 2698.00 | 2359.13 | 338.87 | 6.96 | 3230.34 | 2423.35 | 806.99 | 3.00 | 19.73 |
| Incidence | Slovenia | Both | 875.61 | 725.63 | 149.98 | 4.84 | 1321.07 | 942.07 | 379.00 | 2.49 | 50.87 |
| Incidence | Solomon Islands | Both | 31.94 | 25.70 | 6.24 | 4.12 | 62.27 | 46.86 | 15.41 | 3.04 | 94.97 |
| Incidence | Somalia | Both | 200.73 | 150.33 | 50.40 | 2.98 | 450.59 | 338.28 | 112.31 | 3.01 | 124.47 |
| Incidence | South Africa | Both | 3821.75 | 2671.28 | 1150.48 | 2.32 | 6946.76 | 4787.50 | 2159.26 | 2.22 | 81.77 |
| Incidence | South Korea | Both | 6374.44 | 4774.88 | 1599.56 | 2.99 | 26099.15 | 18618.38 | 7480.77 | 2.49 | 309.43 |
| Incidence | South Sudan | Both | 228.73 | 179.89 | 48.84 | 3.68 | 329.68 | 257.86 | 71.82 | 3.59 | 44.13 |
| Incidence | Spain | Both | 17289.26 | 15623.52 | 1665.74 | 9.38 | 28931.89 | 22886.87 | 6045.02 | 3.79 | 67.34 |
| Incidence | Sri Lanka | Both | 788.42 | 596.46 | 191.96 | 3.11 | 1692.53 | 1225.81 | 466.72 | 2.63 | 114.67 |
| Incidence | Sudan | Both | 776.36 | 605.13 | 171.23 | 3.53 | 1358.64 | 1027.11 | 331.53 | 3.10 | 75.00 |
| Incidence | Suriname | Both | 30.79 | 21.14 | 9.65 | 2.19 | 79.65 | 51.42 | 28.23 | 1.82 | 158.72 |
| Incidence | Swaziland | Both | 49.83 | 38.74 | 11.09 | 3.49 | 85.46 | 65.66 | 19.81 | 3.32 | 71.51 |
| Incidence | Sweden | Both | 2989.59 | 1963.79 | 1025.80 | 1.91 | 4240.68 | 2053.32 | 2187.36 | 0.94 | 41.85 |
| Incidence | Switzerland | Both | 3131.07 | 2521.97 | 609.10 | 4.14 | 4045.47 | 2440.31 | 1605.17 | 1.52 | 29.20 |
| Incidence | Syria | Both | 500.94 | 385.23 | 115.71 | 3.33 | 1194.89 | 923.71 | 271.18 | 3.41 | 138.53 |
| Incidence | Taiwan (Province of China) | Both | 3768.69 | 2683.03 | 1085.66 | 2.47 | 11625.70 | 7244.67 | 4381.03 | 1.65 | 208.48 |
| Incidence | Tajikistan | Both | 375.19 | 269.83 | 105.36 | 2.56 | 393.62 | 263.28 | 130.34 | 2.02 | 4.91 |
| Incidence | Tanzania | Both | 749.09 | 555.56 | 193.53 | 2.87 | 1533.47 | 1100.20 | 433.27 | 2.54 | 104.71 |
| Incidence | Thailand | Both | 10796.05 | 6657.23 | 4138.83 | 1.61 | 18531.20 | 12498.64 | 6032.56 | 2.07 | 71.65 |
| Incidence | Timor-Leste | Both | 44.64 | 31.77 | 12.87 | 2.47 | 139.22 | 102.77 | 36.45 | 2.82 | 211.90 |
| Incidence | Tobago | Both | 93.31 | 70.96 | 22.35 | 3.18 | 173.67 | 128.08 | 45.59 | 2.81 | 86.13 |
| Incidence | Togo | Both | 107.14 | 77.76 | 29.38 | 2.65 | 281.35 | 202.64 | 78.71 | 2.57 | 162.61 |
| Incidence | Tonga | Both | 14.95 | 11.63 | 3.31 | 3.51 | 19.90 | 15.38 | 4.52 | 3.40 | 33.15 |
| Incidence | Trinidad | Both | 93.31 | 70.96 | 22.35 | 3.18 | 173.67 | 128.08 | 45.59 | 2.81 | 86.13 |
| Incidence | Tunisia | Both | 889.85 | 821.65 | 68.20 | 12.05 | 2100.36 | 1876.85 | 223.51 | 8.40 | 136.03 |
| Incidence | Turkey | Both | 14762.93 | 12895.07 | 1867.86 | 6.90 | 27069.03 | 23330.22 | 3738.81 | 6.24 | 83.36 |
| Incidence | Turkmenistan | Both | 285.71 | 225.71 | 60.00 | 3.76 | 387.36 | 292.30 | 95.05 | 3.08 | 35.58 |
| Incidence | Uganda | Both | 338.04 | 219.09 | 118.94 | 1.84 | 780.32 | 523.40 | 256.92 | 2.04 | 130.84 |
| Incidence | UK | Both | 43399.85 | 28957.12 | 14442.73 | 2.00 | 50849.88 | 27228.17 | 23621.71 | 1.15 | 17.17 |
| Incidence | Ukraine | Both | 26644.71 | 21900.56 | 4744.16 | 4.62 | 20415.75 | 16790.65 | 3625.11 | 4.63 | -23.38 |
| Incidence | United Arab Emirates | Both | 52.37 | 43.23 | 9.14 | 4.73 | 462.72 | 395.76 | 66.96 | 5.91 | 783.54 |
| Incidence | Uruguay | Both | 1420.40 | 1236.05 | 184.35 | 6.70 | 1490.21 | 1094.93 | 395.28 | 2.77 | 4.91 |
| Incidence | USA | Both | 174213.44 | 109656.55 | 64556.89 | 1.70 | 247661.38 | 134781.71 | 112879.67 | 1.19 | 42.16 |
| Incidence | Uzbekistan | Both | 1727.13 | 1289.68 | 437.45 | 2.95 | 2258.55 | 1681.87 | 576.68 | 2.92 | 30.77 |
| Incidence | Vanuatu | Both | 16.21 | 12.53 | 3.67 | 3.41 | 38.71 | 29.27 | 9.43 | 3.10 | 138.83 |
| Incidence | Venezuela | Both | 1619.24 | 1046.18 | 573.07 | 1.83 | 4583.62 | 2840.24 | 1743.37 | 1.63 | 183.07 |
| Incidence | Vietnam | Both | 13889.98 | 9787.98 | 4102.00 | 2.39 | 34439.57 | 24157.63 | 10281.95 | 2.35 | 147.95 |
| Incidence | Virgin Islands | Both | 14.95 | 10.50 | 4.46 | 2.36 | 39.54 | 27.14 | 12.40 | 2.19 | 164.39 |
| Incidence | Yemen | Both | 525.29 | 405.86 | 119.44 | 3.40 | 1241.75 | 951.06 | 290.70 | 3.27 | 136.39 |
| Incidence | Zambia | Both | 247.99 | 193.96 | 54.03 | 3.59 | 454.84 | 339.87 | 114.97 | 2.96 | 83.41 |
| Incidence | Zimbabwe | Both | 493.44 | 386.83 | 106.61 | 3.63 | 701.58 | 475.19 | 226.39 | 2.10 | 42.18 |
