# Supplementary material for: Epidemiological trends of tracheal, bronchus, and lung cancer at the global, regional, and national levels: a population-based study
Source: J Hematol Oncol. 2020 Jul 20;13:98. doi: 10.1186/s13045-020-00915-0 (PMC7370495; doi:10.1186/s13045-020-00915-0)
Supplement: Supplementary file 6 — Additional file 6: Table S3. The ASRs and variations of TBL cancer from 1990 to 2017 among countries. [file 13045_2020_915_MOESM6_ESM.docx]

**Table S3. The ASRs and variations of TBL cancer from 1990 to 2017 among countries.**

| **Measure** | **location** | **1990.00** | | | | **2017** | | | | **1990-2017** | | |
| --- | --- | --- | --- | --- | --- | --- | --- | --- | --- | --- | --- | --- |
|  |  | **ASR (95%CI)** | **Female (No.)** | **Male (No.)** | **Male/Female ratio** | **ASR (95%CI)** | **Female (No.)** | **Male (No.)** | **Male/Female ratio** | **EAPC** | **lower** | **upper** |
| DALYs | Afghanistan | 313.42(538.57-159.68) | 141.53 | 450.64 | 3.18 | 286.73(431.91-220.49) | 144.73 | 440.62 | 3.04 | -0.20 | -0.28 | -0.12 |
| DALYs | Albania | 599.28(630.55-565.05) | 181.10 | 1034.05 | 5.71 | 540.56(668.38-439.87) | 185.27 | 919.61 | 4.96 | -0.36 | -0.55 | -0.18 |
| DALYs | Algeria | 235.61(267.03-207.08) | 76.65 | 401.25 | 5.23 | 193.19(215.79-170.80) | 76.98 | 305.56 | 3.97 | -0.56 | -0.70 | -0.43 |
| DALYs | American Samoa | 595.55(657.82-537.65) | 261.04 | 919.86 | 3.52 | 547.13(622.65-477.81) | 310.63 | 798.70 | 2.57 | -0.19 | -0.29 | -0.09 |
| DALYs | Andorra | 716.27(847.31-605.72) | 324.79 | 1083.26 | 3.34 | 537.68(639.77-450.36) | 347.89 | 724.01 | 2.08 | -1.08 | -1.13 | -1.02 |
| DALYs | Angola | 351.04(453.96-258.81) | 138.56 | 558.11 | 4.03 | 263.74(321.48-214.30) | 121.58 | 435.29 | 3.58 | -1.09 | -1.19 | -0.99 |
| DALYs | Antigua | 196.06(212.15-181.15) | 95.37 | 317.93 | 3.33 | 172.44(188.38-158.52) | 126.54 | 224.08 | 1.77 | -0.42 | -0.47 | -0.37 |
| DALYs | Barbuda | 196.06(212.15-181.15) | 95.37 | 317.93 | 3.33 | 172.44(188.38-158.52) | 126.54 | 224.08 | 1.77 | -1.19 | -1.27 | -1.10 |
| DALYs | Argentina | 645.87(666.61-625.45) | 214.97 | 1149.33 | 5.35 | 483.94(541.73-430.45) | 289.16 | 719.21 | 2.49 | -0.34 | -0.61 | -0.06 |
| DALYs | Armenia | 781.23(826.68-741.43) | 226.18 | 1468.34 | 6.49 | 679.56(715.86-642.23) | 201.73 | 1296.70 | 6.43 | -1.67 | -1.75 | -1.59 |
| DALYs | Australia | 671.71(686.99-656.26) | 337.03 | 1060.73 | 3.15 | 448.86(495.90-401.17) | 348.97 | 559.48 | 1.60 | -0.73 | -0.96 | -0.50 |
| DALYs | Austria | 649.68(668.79-631.38) | 259.35 | 1186.13 | 4.57 | 549.25(589.68-514.50) | 407.88 | 713.76 | 1.75 | -0.15 | -0.57 | 0.27 |
| DALYs | Azerbaijan | 520.36(553.95-488.06) | 161.78 | 977.54 | 6.04 | 507.52(589.42-413.25) | 185.06 | 903.87 | 4.88 | -0.47 | -0.59 | -0.35 |
| DALYs | Bahrain | 738.99(804.90-657.99) | 329.88 | 1080.90 | 3.28 | 241.02(270.77-213.37) | 137.54 | 321.84 | 2.34 | -4.91 | -5.31 | -4.51 |
| DALYs | Bangladesh | 554.47(674.86-468.88) | 246.34 | 806.16 | 3.27 | 472.36(545.67-409.45) | 248.11 | 673.18 | 2.71 | -0.47 | -0.71 | -0.23 |
| DALYs | Barbados | 203.93(217.48-191.42) | 103.46 | 336.56 | 3.25 | 179.16(197.79-161.98) | 119.51 | 250.38 | 2.10 | -0.41 | -0.51 | -0.31 |
| DALYs | Belarus | 771.00(796.93-745.89) | 147.41 | 1691.38 | 11.47 | 485.21(529.15-444.98) | 102.67 | 1040.95 | 10.14 | -0.42 | -0.47 | -0.37 |
| DALYs | Belgium | 1036.44(1065.04-1007.53) | 280.71 | 1980.67 | 7.06 | 711.02(759.29-661.26) | 432.81 | 1029.48 | 2.38 | -2.16 | -2.48 | -1.85 |
| DALYs | Belize | 225.57(248.21-205.29) | 117.69 | 331.01 | 2.81 | 286.57(311.02-264.88) | 165.62 | 402.55 | 2.43 | -1.23 | -1.35 | -1.10 |
| DALYs | Benin | 220.46(256.07-187.51) | 108.67 | 338.79 | 3.12 | 200.35(250.62-160.71) | 104.95 | 308.49 | 2.94 | 0.69 | 0.25 | 1.13 |
| DALYs | Bermuda | 694.36(741.57-650.93) | 342.60 | 1134.89 | 3.31 | 434.66(477.69-396.46) | 220.61 | 696.02 | 3.15 | -0.16 | -0.24 | -0.09 |
| DALYs | Bhutan | 197.06(243.57-162.28) | 114.68 | 276.13 | 2.41 | 165.01(211.81-127.44) | 104.06 | 223.75 | 2.15 | -1.56 | -1.68 | -1.45 |
| DALYs | Bolivia | 291.07(355.46-232.57) | 171.94 | 425.12 | 2.47 | 251.31(313.46-196.14) | 193.34 | 317.60 | 1.64 | -0.51 | -0.65 | -0.38 |
| DALYs | Bosnia and Herzegovina | 856.54(898.99-817.22) | 244.95 | 1625.06 | 6.63 | 863.29(946.35-786.20) | 340.36 | 1472.81 | 4.33 | -0.61 | -0.65 | -0.57 |
| DALYs | Botswana | 375.15(451.29-315.80) | 165.35 | 631.02 | 3.82 | 262.20(316.48-219.78) | 172.83 | 378.68 | 2.19 | -0.32 | -0.47 | -0.17 |
| DALYs | Brazil | 366.27(372.96-358.99) | 215.57 | 535.86 | 2.49 | 315.03(322.90-307.80) | 247.93 | 398.12 | 1.61 | -1.31 | -1.48 | -1.13 |
| DALYs | Brunei | 767.61(844.10-681.34) | 587.81 | 935.01 | 1.59 | 645.32(710.51-581.28) | 548.57 | 758.75 | 1.38 | -0.66 | -0.81 | -0.50 |
| DALYs | Bulgaria | 651.30(671.65-630.90) | 173.81 | 1172.09 | 6.74 | 718.03(778.65-656.37) | 274.60 | 1228.94 | 4.48 | -0.19 | -0.44 | 0.05 |
| DALYs | Burkina Faso | 174.74(206.82-131.81) | 102.31 | 253.07 | 2.47 | 166.85(196.38-138.02) | 94.07 | 253.63 | 2.70 | 0.41 | 0.31 | 0.52 |
| DALYs | Burundi | 223.02(270.45-167.25) | 95.87 | 386.53 | 4.03 | 160.28(205.42-125.69) | 70.49 | 248.49 | 3.52 | -0.21 | -0.32 | -0.10 |
| DALYs | Cambodia | 585.09(745.42-476.23) | 266.70 | 1009.26 | 3.78 | 465.13(552.82-389.26) | 221.21 | 815.11 | 3.68 | -1.44 | -1.60 | -1.29 |
| DALYs | Cameroon | 240.81(294.39-191.12) | 115.05 | 371.65 | 3.23 | 238.78(303.81-184.40) | 114.41 | 370.59 | 3.24 | -0.87 | -1.02 | -0.71 |
| DALYs | Canada | 1003.33(1024.45-981.84) | 638.25 | 1447.89 | 2.27 | 643.86(689.88-600.00) | 570.62 | 729.15 | 1.28 | 0.10 | 0.00 | 0.20 |
| DALYs | Cape Verde | 199.63(220.16-179.63) | 145.08 | 277.87 | 1.92 | 242.08(269.35-216.33) | 164.83 | 342.93 | 2.08 | -1.76 | -1.88 | -1.64 |
| DALYs | Central African Republic | 314.48(434.30-224.04) | 129.47 | 518.57 | 4.01 | 271.31(390.43-190.56) | 108.90 | 449.85 | 4.13 | 0.75 | 0.59 | 0.90 |
| DALYs | Chad | 191.58(250.85-147.97) | 107.84 | 280.16 | 2.60 | 250.71(324.17-194.01) | 127.61 | 351.65 | 2.76 | -0.56 | -0.67 | -0.45 |
| DALYs | Chile | 396.52(409.69-383.28) | 200.53 | 629.27 | 3.14 | 311.78(349.00-279.70) | 235.84 | 402.43 | 1.71 | 1.30 | 1.21 | 1.39 |
| DALYs | China | 670.03(716.67-639.38) | 417.40 | 930.93 | 2.23 | 757.61(789.62-721.96) | 451.83 | 1075.36 | 2.38 | -0.64 | -0.75 | -0.53 |
| DALYs | Colombia | 311.71(321.51-302.33) | 211.81 | 417.27 | 1.97 | 213.66(239.26-191.68) | 166.64 | 269.46 | 1.62 | 0.43 | 0.32 | 0.54 |
| DALYs | Comoros | 248.90(305.82-196.81) | 149.26 | 350.18 | 2.35 | 178.27(230.71-145.14) | 110.11 | 263.40 | 2.39 | -1.71 | -1.85 | -1.57 |
| DALYs | Republic of Congo | 366.88(440.69-263.93) | 172.05 | 611.92 | 3.56 | 287.34(366.54-221.12) | 176.96 | 407.49 | 2.30 | -1.32 | -1.48 | -1.16 |
| DALYs | Costa Rica | 242.59(252.93-232.81) | 130.67 | 361.00 | 2.76 | 192.96(208.98-176.75) | 108.59 | 290.26 | 2.67 | -1.18 | -1.39 | -0.96 |
| DALYs | Ivory Coast | 167.45(196.05-143.05) | 81.05 | 240.91 | 2.97 | 143.99(177.57-116.86) | 72.57 | 209.02 | 2.88 | -0.72 | -0.88 | -0.56 |
| DALYs | Croatia | 944.10(975.87-914.40) | 241.46 | 1851.91 | 7.67 | 736.21(791.05-689.26) | 334.32 | 1217.25 | 3.64 | 0.19 | 0.07 | 0.32 |
| DALYs | Cuba | 680.23(698.70-661.74) | 382.07 | 987.54 | 2.58 | 680.42(763.27-611.07) | 481.94 | 900.61 | 1.87 | 0.70 | 0.44 | 0.96 |
| DALYs | Cyprus | 448.81(503.76-382.05) | 159.61 | 778.00 | 4.87 | 467.74(522.27-417.87) | 179.00 | 776.21 | 4.34 | -2.41 | -2.48 | -2.34 |
| DALYs | Czech Republic | 1157.02(1186.29-1126.72) | 284.28 | 2257.11 | 7.94 | 597.70(640.64-558.14) | 344.68 | 899.39 | 2.61 | -0.78 | -1.00 | -0.56 |
| DALYs | Democratic Republic of the Congo | 234.32(299.59-186.31) | 115.02 | 368.90 | 3.21 | 197.97(275.18-152.49) | 99.44 | 319.65 | 3.21 | -1.28 | -1.47 | -1.10 |
| DALYs | Denmark | 988.09(1011.33-965.46) | 697.75 | 1351.77 | 1.94 | 718.76(770.37-667.09) | 665.63 | 783.05 | 1.18 | -0.50 | -0.63 | -0.37 |
| DALYs | Djibouti | 238.95(316.18-176.04) | 138.38 | 336.69 | 2.43 | 223.13(326.90-157.03) | 110.97 | 320.01 | 2.88 | 0.45 | 0.37 | 0.52 |
| DALYs | Dominica | 278.30(297.47-260.08) | 137.84 | 470.37 | 3.41 | 310.84(340.45-283.11) | 189.89 | 430.07 | 2.26 | 0.77 | 0.57 | 0.97 |
| DALYs | Dominican Republic | 221.94(242.51-202.92) | 155.46 | 289.76 | 1.86 | 287.61(336.27-240.10) | 209.86 | 371.19 | 1.77 | 0.14 | -0.08 | 0.35 |
| DALYs | Ecuador | 176.60(183.23-170.45) | 113.05 | 242.59 | 2.15 | 178.96(198.94-161.68) | 153.86 | 206.37 | 1.34 | 0.82 | 0.64 | 1.00 |
| DALYs | Egypt | 144.77(157.05-133.72) | 79.01 | 206.66 | 2.62 | 177.84(207.11-150.21) | 100.37 | 248.24 | 2.47 | 0.97 | 0.83 | 1.11 |
| DALYs | El Salvador | 154.25(164.92-144.82) | 107.93 | 207.17 | 1.92 | 197.63(232.67-166.98) | 145.97 | 266.48 | 1.83 | -0.35 | -0.61 | -0.09 |
| DALYs | Equatorial Guinea | 285.11(425.30-178.37) | 108.51 | 496.13 | 4.57 | 245.51(366.25-155.99) | 152.21 | 373.89 | 2.46 | -1.13 | -1.34 | -0.93 |
| DALYs | Eritrea | 262.71(332.77-203.78) | 106.64 | 484.43 | 4.54 | 214.07(264.89-174.16) | 111.68 | 380.01 | 3.40 | -2.11 | -2.39 | -1.83 |
| DALYs | Estonia | 931.47(962.32-900.73) | 218.32 | 2016.92 | 9.24 | 555.39(640.16-483.25) | 222.66 | 1040.02 | 4.67 | -1.95 | -2.14 | -1.76 |
| DALYs | Ethiopia | 283.22(352.85-207.76) | 82.14 | 481.42 | 5.86 | 175.72(198.62-154.95) | 87.03 | 258.51 | 2.97 | -0.19 | -0.27 | -0.11 |
| DALYs | Micronesia | 571.22(705.76-483.50) | 313.16 | 839.61 | 2.68 | 550.55(683.47-445.29) | 304.00 | 834.56 | 2.75 | -1.52 | -1.65 | -1.39 |
| DALYs | Fiji | 186.22(221.07-158.49) | 126.77 | 246.75 | 1.95 | 176.32(204.55-151.58) | 114.51 | 247.63 | 2.16 | -0.05 | -0.25 | 0.14 |
| DALYs | Finland | 615.34(632.25-599.57) | 213.34 | 1184.07 | 5.55 | 401.71(434.39-371.53) | 261.58 | 565.44 | 2.16 | -0.31 | -0.39 | -0.23 |
| DALYs | France | 749.64(773.07-729.80) | 170.86 | 1433.76 | 8.39 | 706.80(764.21-650.35) | 389.48 | 1065.78 | 2.74 | -0.05 | -0.12 | 0.01 |
| DALYs | Gabon | 343.76(437.94-213.80) | 142.36 | 590.37 | 4.15 | 318.66(394.45-246.37) | 142.67 | 505.59 | 3.54 | 1.55 | 0.77 | 2.34 |
| DALYs | Georgia | 538.93(570.91-506.32) | 161.03 | 1047.96 | 6.51 | 668.38(715.77-623.81) | 163.29 | 1316.46 | 8.06 | -0.74 | -0.89 | -0.60 |
| DALYs | Germany | 744.02(763.65-725.36) | 256.79 | 1401.36 | 5.46 | 637.82(716.22-568.95) | 433.91 | 868.67 | 2.00 | 0.47 | 0.41 | 0.53 |
| DALYs | Ghana | 141.75(168.96-119.24) | 78.64 | 208.59 | 2.65 | 154.41(178.38-128.99) | 80.94 | 246.90 | 3.05 | -0.23 | -0.30 | -0.16 |
| DALYs | Greece | 776.09(798.96-754.04) | 221.17 | 1408.10 | 6.37 | 738.24(794.45-684.94) | 318.71 | 1206.86 | 3.79 | -1.82 | -1.90 | -1.74 |
| DALYs | Greenland | 2468.23(2664.57-2269.64) | 1910.86 | 3138.13 | 1.64 | 1584.94(1709.38-1458.64) | 1244.05 | 1892.33 | 1.52 | 0.48 | 0.14 | 0.83 |
| DALYs | Grenada | 286.36(305.57-268.00) | 151.08 | 463.95 | 3.07 | 294.74(318.25-271.97) | 206.72 | 377.73 | 1.83 | 0.49 | 0.37 | 0.61 |
| DALYs | Guam | 802.02(878.71-727.20) | 377.63 | 1229.63 | 3.26 | 840.02(921.62-754.99) | 546.68 | 1138.13 | 2.08 | 0.12 | 0.02 | 0.22 |
| DALYs | Guatemala | 172.86(180.52-165.78) | 140.53 | 205.45 | 1.46 | 140.28(155.76-126.26) | 93.79 | 195.62 | 2.09 | -1.53 | -1.86 | -1.20 |
| DALYs | Guinea | 198.66(225.26-174.09) | 97.44 | 299.42 | 3.07 | 225.52(282.60-158.42) | 93.44 | 352.72 | 3.77 | 0.71 | 0.61 | 0.81 |
| DALYs | Guinea-Bissau | 301.41(369.84-228.93) | 114.82 | 490.22 | 4.27 | 237.57(299.92-179.10) | 113.17 | 383.00 | 3.38 | -0.71 | -0.85 | -0.56 |
| DALYs | Guyana | 156.84(166.86-147.50) | 92.44 | 225.68 | 2.44 | 173.99(197.72-154.33) | 130.10 | 223.77 | 1.72 | 0.76 | 0.48 | 1.03 |
| DALYs | Haiti | 385.72(521.87-303.94) | 177.14 | 597.60 | 3.37 | 286.56(409.96-217.14) | 155.28 | 435.58 | 2.81 | -1.00 | -1.12 | -0.88 |
| DALYs | Honduras | 177.99(200.99-155.44) | 109.82 | 248.36 | 2.26 | 176.27(215.86-142.45) | 126.15 | 230.59 | 1.83 | -0.06 | -0.11 | -0.01 |
| DALYs | Hungary | 1202.93(1240.26-1167.57) | 439.91 | 2172.96 | 4.94 | 1115.57(1187.23-1050.02) | 718.98 | 1631.33 | 2.27 | -0.19 | -0.60 | 0.22 |
| DALYs | Iceland | 729.11(764.05-696.47) | 629.87 | 845.49 | 1.34 | 563.55(597.80-532.16) | 501.42 | 630.58 | 1.26 | -1.11 | -1.22 | -1.00 |
| DALYs | India | 168.82(185.50-154.45) | 69.89 | 261.91 | 3.75 | 178.69(192.39-166.65) | 103.01 | 259.28 | 2.52 | 0.16 | -0.04 | 0.37 |
| DALYs | Indonesia | 385.17(449.94-334.25) | 203.61 | 580.89 | 2.85 | 408.83(477.19-349.05) | 192.66 | 649.22 | 3.37 | 0.15 | 0.08 | 0.21 |
| DALYs | Iran | 228.01(254.24-210.75) | 115.38 | 332.64 | 2.88 | 237.50(248.34-229.05) | 145.07 | 331.43 | 2.28 | 0.60 | 0.23 | 0.98 |
| DALYs | Iraq | 408.76(490.93-338.82) | 163.44 | 658.48 | 4.03 | 212.07(231.85-195.14) | 125.84 | 295.51 | 2.35 | -2.65 | -2.93 | -2.37 |
| DALYs | Ireland | 803.59(828.15-777.97) | 481.30 | 1178.03 | 2.45 | 541.97(586.43-500.12) | 465.94 | 626.05 | 1.34 | -1.45 | -1.59 | -1.30 |
| DALYs | Israel | 464.65(481.32-447.76) | 257.95 | 705.79 | 2.74 | 415.43(447.12-386.93) | 249.85 | 601.75 | 2.41 | -0.63 | -0.83 | -0.43 |
| DALYs | Italy | 823.10(842.56-803.41) | 237.58 | 1532.84 | 6.45 | 486.83(520.12-455.93) | 274.73 | 736.86 | 2.68 | -1.99 | -2.06 | -1.92 |
| DALYs | Jamaica | 344.71(368.51-320.73) | 141.08 | 568.91 | 4.03 | 465.28(544.20-394.27) | 171.50 | 772.22 | 4.50 | -0.71 | -0.85 | -0.57 |
| DALYs | Japan | 436.11(440.93-430.69) | 210.78 | 731.72 | 3.47 | 372.70(386.62-360.60) | 186.54 | 590.18 | 3.16 | 0.54 | 0.04 | 1.05 |
| DALYs | Jordan | 311.81(366.60-238.15) | 101.86 | 509.23 | 5.00 | 267.76(310.24-230.04) | 121.32 | 402.66 | 3.32 | -0.68 | -0.82 | -0.54 |
| DALYs | Kazakhstan | 973.53(1008.20-938.67) | 276.04 | 1968.27 | 7.13 | 457.72(493.28-425.34) | 152.43 | 881.87 | 5.79 | -0.13 | -0.44 | 0.18 |
| DALYs | Kenya | 140.92(197.61-115.85) | 73.78 | 209.16 | 2.84 | 134.10(182.50-118.74) | 61.96 | 217.61 | 3.51 | -3.20 | -3.42 | -2.98 |
| DALYs | Kiribati | 376.84(415.71-340.88) | 242.19 | 544.25 | 2.25 | 378.69(458.17-320.21) | 229.36 | 578.22 | 2.52 | -0.30 | -0.49 | -0.11 |
| DALYs | Kuwait | 236.91(252.70-222.13) | 130.52 | 301.22 | 2.31 | 147.17(162.27-133.31) | 68.26 | 197.77 | 2.90 | -0.02 | -0.07 | 0.03 |
| DALYs | Kyrgyzstan | 621.36(661.60-582.29) | 189.04 | 1202.59 | 6.36 | 272.93(290.61-254.85) | 117.15 | 472.97 | 4.04 | -1.54 | -1.81 | -1.26 |
| DALYs | Laos | 633.66(787.60-502.63) | 298.39 | 999.35 | 3.35 | 490.35(596.31-396.24) | 272.42 | 725.80 | 2.66 | -2.94 | -3.28 | -2.60 |
| DALYs | Latvia | 846.53(873.66-819.45) | 196.00 | 1837.00 | 9.37 | 577.00(645.43-509.41) | 186.68 | 1156.77 | 6.20 | -1.06 | -1.13 | -1.00 |
| DALYs | Lebanon | 636.66(743.62-528.63) | 263.42 | 1069.17 | 4.06 | 514.40(592.46-451.68) | 386.63 | 670.59 | 1.73 | -1.63 | -1.94 | -1.33 |
| DALYs | Lesotho | 324.60(456.24-262.00) | 109.24 | 584.77 | 5.35 | 407.57(517.90-321.10) | 145.25 | 780.84 | 5.38 | -0.61 | -0.75 | -0.47 |
| DALYs | Liberia | 189.73(251.00-156.73) | 93.49 | 268.06 | 2.87 | 162.43(220.94-130.06) | 85.81 | 236.84 | 2.76 | 1.27 | 0.91 | 1.64 |
| DALYs | Libya | 510.11(619.09-416.06) | 99.16 | 871.19 | 8.79 | 485.91(579.70-398.06) | 118.68 | 842.32 | 7.10 | -0.29 | -0.58 | 0.01 |
| DALYs | Lithuania | 811.20(836.03-786.72) | 172.90 | 1731.42 | 10.01 | 589.49(639.53-546.78) | 172.54 | 1193.38 | 6.92 | -0.04 | -0.15 | 0.07 |
| DALYs | Luxembourg | 890.78(930.31-850.64) | 278.03 | 1671.22 | 6.01 | 637.95(713.06-572.96) | 427.67 | 865.38 | 2.02 | -1.56 | -1.79 | -1.33 |
| DALYs | Macedonia | 610.56(647.85-572.33) | 181.47 | 1072.46 | 5.91 | 768.13(850.16-696.46) | 271.58 | 1292.05 | 4.76 | -1.43 | -1.52 | -1.33 |
| DALYs | Madagascar | 190.03(215.38-166.63) | 101.45 | 272.62 | 2.69 | 160.61(198.78-127.72) | 81.69 | 246.70 | 3.02 | 0.84 | 0.65 | 1.02 |
| DALYs | Malawi | 132.55(162.80-83.56) | 75.22 | 195.55 | 2.60 | 121.91(143.54-103.31) | 62.32 | 200.68 | 3.22 | -0.59 | -0.68 | -0.49 |
| DALYs | Malaysia | 418.63(460.11-383.18) | 198.12 | 655.82 | 3.31 | 407.80(467.33-353.07) | 225.50 | 592.30 | 2.63 | -0.62 | -0.93 | -0.31 |
| DALYs | Maldives | 305.13(379.23-233.86) | 83.88 | 458.84 | 5.47 | 150.18(167.01-133.74) | 59.29 | 230.08 | 3.88 | -0.14 | -0.29 | 0.00 |
| DALYs | Mali | 163.75(187.32-143.98) | 90.40 | 235.00 | 2.60 | 149.18(186.31-120.92) | 87.11 | 206.14 | 2.37 | -3.07 | -3.23 | -2.90 |
| DALYs | Malta | 548.38(575.78-520.78) | 136.89 | 1050.89 | 7.68 | 465.45(497.26-430.95) | 190.34 | 769.73 | 4.04 | -0.35 | -0.47 | -0.23 |
| DALYs | Marshall Islands | 597.02(827.74-359.52) | 189.40 | 1010.58 | 5.34 | 635.71(901.53-415.91) | 246.29 | 1023.83 | 4.16 | -0.83 | -0.93 | -0.73 |
| DALYs | Mauritania | 231.07(279.23-190.51) | 131.81 | 338.97 | 2.57 | 199.49(253.33-157.72) | 127.15 | 270.12 | 2.12 | 0.19 | 0.07 | 0.32 |
| DALYs | Mauritius | 300.57(316.55-284.25) | 131.05 | 508.04 | 3.88 | 252.29(272.02-232.18) | 134.41 | 395.77 | 2.94 | -0.41 | -0.66 | -0.17 |
| DALYs | Mexico | 284.99(289.04-280.94) | 166.17 | 414.64 | 2.50 | 175.53(179.94-171.38) | 112.29 | 248.26 | 2.21 | -0.63 | -0.74 | -0.53 |
| DALYs | Moldova | 703.82(731.42-680.96) | 208.82 | 1357.68 | 6.50 | 484.28(515.23-454.70) | 152.37 | 921.38 | 6.05 | -2.15 | -2.28 | -2.02 |
| DALYs | Mongolia | 831.74(914.65-762.89) | 373.50 | 1380.15 | 3.70 | 558.40(629.11-487.73) | 217.97 | 1011.40 | 4.64 | -0.16 | -0.20 | -0.13 |
| DALYs | Montenegro | 1027.53(1113.45-945.66) | 422.98 | 1759.13 | 4.16 | 1048.64(1192.94-924.48) | 517.50 | 1658.49 | 3.20 | -1.11 | -1.56 | -0.66 |
| DALYs | Morocco | 327.05(384.42-278.49) | 74.32 | 584.28 | 7.86 | 332.80(418.84-263.65) | 79.57 | 584.31 | 7.34 | -2.15 | -2.44 | -1.85 |
| DALYs | Mozambique | 196.04(230.22-169.20) | 75.95 | 326.66 | 4.30 | 221.62(263.85-184.83) | 75.10 | 406.16 | 5.41 | 0.02 | -0.14 | 0.17 |
| DALYs | Myanmar | 572.49(730.19-446.46) | 332.76 | 838.20 | 2.52 | 440.00(498.87-384.16) | 277.67 | 647.16 | 2.33 | 0.00 | -0.11 | 0.10 |
| DALYs | Namibia | 206.19(232.93-182.82) | 112.10 | 317.48 | 2.83 | 160.67(186.66-138.16) | 86.72 | 263.82 | 3.04 | 0.80 | 0.64 | 0.96 |
| DALYs | Nepal | 271.97(415.80-165.75) | 187.68 | 350.93 | 1.87 | 242.32(338.39-158.18) | 191.24 | 297.26 | 1.55 | -0.82 | -0.93 | -0.72 |
| DALYs | Netherlands | 1016.29(1039.69-992.31) | 338.84 | 1880.45 | 5.55 | 757.26(807.46-711.26) | 654.97 | 884.33 | 1.35 | -1.23 | -1.59 | -0.86 |
| DALYs | New Zealand | 736.23(760.67-711.71) | 484.04 | 1043.14 | 2.16 | 497.74(529.09-469.53) | 459.50 | 541.95 | 1.18 | -0.33 | -0.57 | -0.10 |
| DALYs | Nicaragua | 153.31(168.83-139.56) | 95.97 | 215.48 | 2.25 | 132.06(151.13-116.37) | 92.06 | 178.72 | 1.94 | -1.13 | -1.23 | -1.03 |
| DALYs | Niger | 189.65(251.46-138.04) | 99.29 | 269.13 | 2.71 | 174.59(229.47-134.69) | 95.44 | 256.87 | 2.69 | -1.56 | -1.63 | -1.49 |
| DALYs | Nigeria | 177.37(236.68-128.92) | 81.98 | 266.08 | 3.25 | 165.45(228.99-121.72) | 97.68 | 236.88 | 2.43 | -0.40 | -0.56 | -0.24 |
| DALYs | North Korea | 580.40(701.09-471.01) | 318.02 | 999.04 | 3.14 | 575.73(720.45-455.88) | 267.83 | 991.68 | 3.70 | -0.32 | -0.52 | -0.12 |
| DALYs | Northern Mariana Islands | 834.01(1040.86-686.35) | 378.29 | 1176.13 | 3.11 | 647.72(734.86-568.14) | 312.41 | 979.74 | 3.14 | -0.15 | -0.32 | 0.03 |
| DALYs | Norway | 549.75(558.98-541.37) | 303.99 | 841.24 | 2.77 | 466.75(487.72-448.41) | 399.77 | 541.84 | 1.36 | 0.02 | -0.02 | 0.06 |
| DALYs | Oman | 184.19(232.23-146.07) | 73.63 | 279.24 | 3.79 | 141.50(171.67-115.69) | 81.71 | 193.15 | 2.36 | -0.90 | -1.03 | -0.76 |
| DALYs | Pakistan | 301.45(362.77-265.23) | 119.36 | 453.58 | 3.80 | 329.81(405.18-265.64) | 143.01 | 500.82 | 3.50 | -0.53 | -0.81 | -0.26 |
| DALYs | Palestine | 403.13(490.53-323.13) | 163.28 | 685.75 | 4.20 | 340.02(373.08-311.50) | 173.84 | 512.96 | 2.95 | -0.77 | -0.94 | -0.60 |
| DALYs | Panama | 298.63(313.27-285.46) | 140.58 | 451.78 | 3.21 | 200.98(217.93-185.49) | 138.44 | 266.30 | 1.92 | 0.26 | 0.03 | 0.50 |
| DALYs | Papua New Guinea | 640.06(870.40-513.04) | 300.46 | 951.86 | 3.17 | 662.34(935.63-523.36) | 321.33 | 963.64 | 3.00 | -0.67 | -0.76 | -0.58 |
| DALYs | Paraguay | 247.49(273.04-221.85) | 146.29 | 357.76 | 2.45 | 320.92(385.84-266.88) | 158.18 | 495.56 | 3.13 | -1.69 | -1.83 | -1.54 |
| DALYs | Peru | 288.12(318.14-259.40) | 206.32 | 373.90 | 1.81 | 203.06(233.64-174.13) | 179.25 | 228.04 | 1.27 | 0.21 | 0.18 | 0.24 |
| DALYs | Philippines | 408.03(434.47-380.69) | 199.85 | 629.41 | 3.15 | 420.86(482.45-365.29) | 234.36 | 640.64 | 2.73 | 1.02 | 0.66 | 1.39 |
| DALYs | Poland | 1050.66(1076.27-1022.13) | 309.70 | 1990.28 | 6.43 | 889.62(950.39-827.96) | 484.51 | 1403.00 | 2.90 | -1.47 | -1.79 | -1.16 |
| DALYs | Portugal | 445.87(460.12-430.77) | 148.84 | 808.35 | 5.43 | 464.80(506.46-425.92) | 199.33 | 776.25 | 3.89 | -0.27 | -0.55 | 0.02 |
| DALYs | Puerto Rico | 329.53(342.56-316.44) | 185.86 | 495.48 | 2.67 | 224.91(242.31-208.83) | 153.55 | 311.79 | 2.03 | -0.72 | -0.84 | -0.61 |
| DALYs | Qatar | 424.43(505.88-355.07) | 163.33 | 587.44 | 3.60 | 256.92(311.39-208.18) | 131.33 | 312.22 | 2.38 | 0.16 | -0.05 | 0.37 |
| DALYs | Romania | 666.42(687.38-644.14) | 194.70 | 1193.82 | 6.13 | 760.47(813.38-709.50) | 294.53 | 1301.60 | 4.42 | -1.70 | -1.83 | -1.57 |
| DALYs | Russia | 833.30(847.48-814.28) | 192.99 | 1850.85 | 9.59 | 526.21(536.93-515.84) | 152.25 | 1078.57 | 7.08 | -1.99 | -2.35 | -1.63 |
| DALYs | Rwanda | 220.01(275.66-158.12) | 94.21 | 369.89 | 3.93 | 154.47(187.20-125.71) | 82.13 | 262.05 | 3.19 | -1.14 | -1.35 | -0.94 |
| DALYs | Saint Lucia | 282.07(301.71-263.42) | 166.03 | 425.08 | 2.56 | 248.45(270.96-227.69) | 171.53 | 329.97 | 1.92 | 0.28 | 0.12 | 0.45 |
| DALYs | Saint Vincent | 201.50(216.30-186.78) | 130.31 | 288.43 | 2.21 | 229.28(249.78-211.30) | 156.58 | 297.32 | 1.90 | -2.17 | -2.50 | -1.84 |
| DALYs | Grenadines | 201.50(216.30-186.78) | 130.31 | 288.43 | 2.21 | 229.28(249.78-211.30) | 156.58 | 297.32 | 1.90 | -1.82 | -2.06 | -1.59 |
| DALYs | Samoa | 187.21(217.06-157.42) | 97.28 | 276.87 | 2.85 | 171.81(203.54-143.80) | 99.74 | 250.87 | 2.52 | -0.49 | -0.69 | -0.29 |
| DALYs | Sao Tome and Principe | 269.12(311.39-236.89) | 123.94 | 435.29 | 3.51 | 332.57(419.03-245.93) | 150.36 | 540.47 | 3.59 | 0.49 | 0.37 | 0.61 |
| DALYs | Saudi Arabia | 175.87(220.56-138.52) | 54.34 | 261.98 | 4.82 | 190.06(223.31-162.04) | 75.70 | 268.84 | 3.55 | -0.33 | -0.40 | -0.27 |
| DALYs | Senegal | 260.97(330.93-214.89) | 108.17 | 407.89 | 3.77 | 240.25(303.47-194.27) | 110.80 | 379.59 | 3.43 | 0.89 | 0.76 | 1.02 |
| DALYs | Serbia | 1033.27(1131.92-958.43) | 373.35 | 1768.54 | 4.74 | 1023.54(1106.36-947.65) | 573.46 | 1521.73 | 2.65 | 0.73 | 0.43 | 1.02 |
| DALYs | Seychelles | 371.29(408.15-335.14) | 134.28 | 656.42 | 4.89 | 347.10(380.63-315.84) | 149.72 | 559.65 | 3.74 | -0.11 | -0.36 | 0.14 |
| DALYs | Sierra Leone | 229.58(315.83-167.77) | 110.56 | 340.44 | 3.08 | 214.37(279.87-170.39) | 109.73 | 318.52 | 2.90 | 0.46 | 0.26 | 0.65 |
| DALYs | Singapore | 766.91(791.13-744.46) | 398.50 | 1181.57 | 2.97 | 360.99(388.26-334.93) | 210.50 | 538.37 | 2.56 | -0.86 | -1.12 | -0.61 |
| DALYs | Slovakia | 953.88(996.87-912.91) | 190.68 | 1899.38 | 9.96 | 584.60(640.61-534.29) | 265.05 | 992.23 | 3.74 | -0.03 | -0.18 | 0.12 |
| DALYs | Slovenia | 856.55(882.19-830.72) | 251.39 | 1677.93 | 6.67 | 626.63(676.32-578.82) | 346.66 | 944.32 | 2.72 | -3.01 | -3.17 | -2.84 |
| DALYs | Solomon Islands | 558.57(762.77-439.04) | 250.90 | 802.56 | 3.20 | 483.16(668.27-384.45) | 242.68 | 715.08 | 2.95 | -1.72 | -1.81 | -1.62 |
| DALYs | Somalia | 199.81(274.98-128.69) | 95.59 | 304.31 | 3.18 | 178.95(248.15-129.50) | 84.91 | 283.49 | 3.34 | -1.37 | -1.54 | -1.21 |
| DALYs | South Africa | 455.22(559.28-414.61) | 234.71 | 731.28 | 3.12 | 386.57(405.25-367.52) | 201.41 | 631.77 | 3.14 | -0.57 | -0.60 | -0.54 |
| DALYs | South Korea | 495.54(510.26-480.71) | 218.07 | 868.67 | 3.98 | 421.67(457.49-389.34) | 210.62 | 688.46 | 3.27 | -0.69 | -0.83 | -0.56 |
| DALYs | South Sudan | 232.17(326.89-153.13) | 113.80 | 322.54 | 2.83 | 224.10(304.34-164.42) | 101.75 | 326.80 | 3.21 | -0.90 | -1.46 | -0.35 |
| DALYs | Spain | 660.01(678.84-641.97) | 119.93 | 1299.96 | 10.84 | 568.04(609.46-527.15) | 248.46 | 931.49 | 3.75 | -1.10 | -1.61 | -0.57 |
| DALYs | Sri Lanka | 174.54(189.02-161.07) | 85.96 | 261.01 | 3.04 | 159.72(193.89-131.47) | 81.90 | 254.48 | 3.11 | -0.33 | -0.46 | -0.20 |
| DALYs | Sudan | 194.94(294.54-143.54) | 90.81 | 288.22 | 3.17 | 179.42(242.14-137.84) | 92.46 | 251.15 | 2.72 | -0.53 | -0.68 | -0.39 |
| DALYs | Suriname | 286.04(310.98-263.64) | 176.28 | 403.05 | 2.29 | 328.48(367.04-289.94) | 219.78 | 453.20 | 2.06 | 0.06 | -0.13 | 0.25 |
| DALYs | Swaziland | 417.12(522.35-330.22) | 165.38 | 722.30 | 4.37 | 384.72(496.28-240.01) | 147.23 | 707.35 | 4.80 | -0.21 | -0.26 | -0.16 |
| DALYs | Sweden | 444.50(456.17-433.92) | 298.90 | 618.13 | 2.07 | 372.50(394.11-351.39) | 375.66 | 372.69 | 0.99 | 0.66 | 0.45 | 0.86 |
| DALYs | Switzerland | 679.96(696.94-662.25) | 254.27 | 1199.65 | 4.72 | 435.30(468.36-404.14) | 336.11 | 548.16 | 1.63 | 0.14 | -0.31 | 0.60 |
| DALYs | Syria | 227.07(260.75-198.15) | 109.59 | 333.93 | 3.05 | 222.06(267.01-184.81) | 106.52 | 328.38 | 3.08 | -0.53 | -0.71 | -0.36 |
| DALYs | Taiwan (Province of China) | 509.80(522.63-498.45) | 325.67 | 668.54 | 2.05 | 549.63(583.86-516.08) | 384.50 | 737.90 | 1.92 | -1.53 | -1.71 | -1.36 |
| DALYs | Tajikistan | 335.66(354.95-316.82) | 169.64 | 528.08 | 3.11 | 185.56(210.33-159.85) | 117.13 | 259.81 | 2.22 | -0.07 | -0.29 | 0.15 |
| DALYs | Tanzania | 159.92(192.14-125.93) | 82.65 | 238.16 | 2.88 | 154.46(182.84-127.94) | 82.78 | 232.63 | 2.81 | 0.24 | 0.02 | 0.46 |
| DALYs | Thailand | 719.66(764.78-672.95) | 517.98 | 945.02 | 1.82 | 435.33(491.66-387.86) | 261.07 | 640.01 | 2.45 | -1.76 | -2.49 | -1.01 |
| DALYs | Bahamas | 377.69(406.12-349.40) | 161.25 | 642.49 | 3.98 | 311.72(345.70-279.22) | 181.48 | 464.68 | 2.56 | -0.36 | -0.51 | -0.21 |
| DALYs | Gambia | 178.60(214.66-144.69) | 86.17 | 257.37 | 2.99 | 171.68(204.33-139.39) | 92.62 | 254.00 | 2.74 | -2.32 | -2.50 | -2.14 |
| DALYs | Timor-Leste | 379.90(506.70-299.45) | 222.64 | 529.77 | 2.38 | 404.73(554.62-308.83) | 216.75 | 596.80 | 2.75 | 0.36 | 0.16 | 0.55 |
| DALYs | Togo | 206.10(237.97-176.40) | 107.22 | 311.71 | 2.91 | 198.74(244.82-159.39) | 96.99 | 337.63 | 3.48 | -0.51 | -0.62 | -0.40 |
| DALYs | Tonga | 617.90(683.84-559.19) | 272.49 | 975.86 | 3.58 | 580.70(672.95-501.45) | 237.88 | 970.43 | 4.08 | -0.06 | -0.13 | 0.01 |
| DALYs | Trinidad | 264.75(279.87-249.91) | 120.22 | 420.10 | 3.49 | 234.38(284.12-189.92) | 118.29 | 356.40 | 3.01 | -0.17 | -0.20 | -0.13 |
| DALYs | Tobago | 264.75(279.87-249.91) | 120.22 | 420.10 | 3.49 | 234.38(284.12-189.92) | 118.29 | 356.40 | 3.01 | -0.51 | -0.62 | -0.40 |
| DALYs | Tunisia | 378.17(426.73-332.02) | 61.39 | 670.89 | 10.93 | 378.76(483.04-292.01) | 78.03 | 690.27 | 8.85 | -0.34 | -0.55 | -0.13 |
| DALYs | Turkey | 1008.80(1128.77-872.98) | 253.26 | 1811.69 | 7.15 | 740.09(821.25-656.89) | 196.75 | 1346.88 | 6.85 | -1.03 | -1.21 | -0.85 |
| DALYs | Turkmenistan | 371.39(388.53-355.23) | 135.83 | 662.73 | 4.88 | 248.79(273.39-225.39) | 111.81 | 413.34 | 3.70 | -1.89 | -2.38 | -1.40 |
| DALYs | Uganda | 124.08(149.93-102.37) | 89.53 | 156.68 | 1.75 | 142.67(164.83-121.11) | 83.50 | 219.95 | 2.63 | 0.27 | 0.03 | 0.52 |
| DALYs | Ukraine | 872.38(903.54-840.84) | 225.56 | 1828.25 | 8.11 | 545.29(584.52-509.30) | 148.36 | 1116.09 | 7.52 | -1.73 | -1.82 | -1.64 |
| DALYs | United Arab Emirates | 303.99(379.01-242.82) | 148.34 | 415.51 | 2.80 | 344.48(433.59-270.35) | 165.92 | 439.99 | 2.65 | -2.35 | -2.70 | -2.01 |
| DALYs | UK | 940.24(949.10-931.04) | 563.42 | 1424.38 | 2.53 | 599.35(610.82-588.81) | 515.68 | 694.98 | 1.35 | 0.51 | 0.34 | 0.69 |
| DALYs | USA | 1092.42(1103.15-1081.45) | 725.64 | 1553.17 | 2.14 | 709.08(729.63-689.07) | 583.02 | 854.61 | 1.47 | -1.03 | -1.15 | -0.91 |
| DALYs | Uruguay | 927.84(961.07-893.70) | 211.73 | 1771.30 | 8.37 | 708.50(797.35-627.40) | 340.88 | 1162.52 | 3.41 | -1.94 | -2.09 | -1.79 |
| DALYs | Uzbekistan | 384.17(397.73-371.62) | 168.53 | 653.83 | 3.88 | 246.55(278.53-217.91) | 116.28 | 413.94 | 3.56 | -1.61 | -1.91 | -1.31 |
| DALYs | Vanuatu | 596.63(826.28-457.84) | 297.00 | 841.06 | 2.83 | 568.70(795.93-430.70) | 289.86 | 823.67 | 2.84 | -0.10 | -0.13 | -0.06 |
| DALYs | Venezuela | 399.44(413.78-385.08) | 267.96 | 544.08 | 2.03 | 382.79(444.13-332.46) | 274.85 | 505.02 | 1.84 | -0.04 | -0.16 | 0.07 |
| DALYs | Vietnam | 816.26(936.40-708.67) | 417.14 | 1347.32 | 3.23 | 914.17(1065.95-781.45) | 461.24 | 1499.02 | 3.25 | 0.57 | 0.46 | 0.67 |
| DALYs | Virgin Islands | 408.75(449.09-373.47) | 224.59 | 621.58 | 2.77 | 481.60(548.39-399.02) | 276.82 | 724.12 | 2.62 | 0.74 | 0.66 | 0.83 |
| DALYs | Yemen | 249.90(382.28-153.13) | 112.30 | 388.83 | 3.46 | 232.19(322.83-174.52) | 106.47 | 360.73 | 3.39 | -0.16 | -0.21 | -0.12 |
| DALYs | Zambia | 209.03(246.22-173.79) | 94.97 | 306.48 | 3.23 | 176.64(203.34-149.70) | 83.65 | 281.43 | 3.36 | -1.11 | -1.32 | -0.90 |
| DALYs | Zimbabwe | 282.67(324.78-242.98) | 120.70 | 449.64 | 3.73 | 254.08(297.21-211.98) | 143.97 | 401.21 | 2.79 | -0.26 | -0.84 | 0.34 |
| Deaths | Afghanistan | 13.58(23.01-7.61) | 5.86 | 19.52 | 3.33 | 144.73(180.66-112.84) | 6.00 | 18.84 | 3.14 | 0.32 | 0.20 | 0.43 |
| Deaths | Albania | 24.78(26.09-23.33) | 7.15 | 45.23 | 6.33 | 185.27(243.25-136.55) | 7.82 | 42.69 | 5.46 | -0.23 | -0.63 | 0.18 |
| Deaths | Algeria | 10.39(11.66-9.21) | 3.31 | 17.71 | 5.35 | 76.98(88.00-66.91) | 3.39 | 13.75 | 4.06 | 0.23 | 0.11 | 0.35 |
| Deaths | American Samoa | 27.47(30.25-24.81) | 12.35 | 42.99 | 3.48 | 310.63(352.64-268.38) | 14.38 | 37.54 | 2.61 | 0.90 | 0.81 | 0.99 |
| Deaths | Andorra | 33.51(39.41-28.37) | 14.49 | 52.31 | 3.61 | 347.89(474.46-250.87) | 16.28 | 36.50 | 2.24 | 0.20 | 0.06 | 0.33 |
| Deaths | Angola | 14.74(18.94-11.25) | 5.81 | 23.56 | 4.05 | 121.58(162.40-90.92) | 5.24 | 19.26 | 3.68 | -0.40 | -0.54 | -0.25 |
| Deaths | Antigua | 8.56(9.24-7.94) | 4.03 | 14.46 | 3.59 | 126.54(140.92-113.21) | 5.79 | 10.51 | 1.81 | 1.20 | 1.15 | 1.25 |
| Deaths | Barbuda | 8.56(9.24-7.94) | 4.03 | 14.46 | 3.59 | 126.54(140.92-113.21) | 5.79 | 10.51 | 1.81 | 1.34 | 1.24 | 1.44 |
| Deaths | Argentina | 25.91(26.71-25.13) | 9.00 | 46.94 | 5.21 | 289.16(337.99-246.61) | 12.30 | 32.66 | 2.65 | 0.33 | -0.06 | 0.71 |
| Deaths | Armenia | 28.68(30.31-27.24) | 8.49 | 55.29 | 6.51 | 201.73(219.28-183.95) | 8.89 | 56.50 | 6.36 | 0.06 | -0.03 | 0.16 |
| Deaths | Australia | 30.16(30.84-29.48) | 14.44 | 50.41 | 3.49 | 348.97(404.64-299.74) | 16.44 | 28.68 | 1.74 | 1.73 | 1.57 | 1.90 |
| Deaths | Austria | 27.55(28.31-26.82) | 11.09 | 53.30 | 4.80 | 407.88(460.10-364.20) | 17.35 | 33.38 | 1.92 | 0.72 | 0.42 | 1.01 |
| Deaths | Azerbaijan | 18.75(19.87-17.68) | 6.21 | 36.15 | 5.82 | 185.06(215.79-158.19) | 7.14 | 37.09 | 5.19 | 0.87 | 0.71 | 1.03 |
| Deaths | Bahrain | 39.57(43.35-35.24) | 17.29 | 58.52 | 3.38 | 137.54(160.80-116.88) | 7.47 | 18.46 | 2.47 | -3.74 | -4.17 | -3.31 |
| Deaths | Bangladesh | 22.36(26.76-19.00) | 8.83 | 33.41 | 3.78 | 248.11(301.03-200.15) | 8.91 | 32.07 | 3.60 | -0.40 | -1.02 | 0.22 |
| Deaths | Barbados | 9.16(9.80-8.62) | 4.54 | 15.76 | 3.47 | 119.51(139.81-102.41) | 5.38 | 11.75 | 2.18 | 0.84 | 0.70 | 0.98 |
| Deaths | Belarus | 28.68(29.61-27.81) | 6.23 | 65.37 | 10.49 | 102.67(121.00-81.65) | 4.40 | 44.31 | 10.06 | 1.20 | 1.15 | 1.25 |
| Deaths | Belgium | 45.68(46.96-44.44) | 11.59 | 93.87 | 8.10 | 432.81(487.68-385.09) | 18.07 | 50.51 | 2.79 | -1.63 | -1.79 | -1.47 |
| Deaths | Belize | 10.22(11.25-9.31) | 5.14 | 15.44 | 3.00 | 165.62(189.94-143.69) | 6.90 | 17.34 | 2.51 | 2.20 | 1.95 | 2.45 |
| Deaths | Benin | 10.17(11.78-8.67) | 4.88 | 15.68 | 3.22 | 104.95(135.66-78.46) | 4.82 | 14.92 | 3.09 | 1.51 | 1.19 | 1.84 |
| Deaths | Bermuda | 31.26(33.20-29.36) | 15.53 | 53.20 | 3.43 | 220.61(264.93-182.00) | 11.11 | 34.67 | 3.12 | -0.01 | -0.06 | 0.04 |
| Deaths | Bhutan | 8.38(10.26-6.86) | 4.86 | 11.89 | 2.45 | 104.06(131.98-79.89) | 4.50 | 10.83 | 2.41 | -1.77 | -1.92 | -1.62 |
| Deaths | Bolivia | 12.90(15.79-10.49) | 7.47 | 19.17 | 2.57 | 193.34(259.21-138.68) | 8.79 | 15.99 | 1.82 | -0.35 | -0.45 | -0.25 |
| Deaths | Bosnia and Herzegovina | 33.95(35.68-32.41) | 10.02 | 65.96 | 6.58 | 340.36(386.99-299.77) | 14.37 | 64.24 | 4.47 | 0.28 | 0.12 | 0.45 |
| Deaths | Botswana | 15.65(18.34-13.37) | 7.11 | 26.69 | 3.75 | 172.83(221.48-138.14) | 7.85 | 18.29 | 2.33 | 1.25 | 1.15 | 1.35 |
| Deaths | Brazil | 15.84(16.11-15.51) | 9.28 | 23.52 | 2.53 | 247.93(258.33-238.74) | 10.86 | 19.06 | 1.75 | 0.75 | 0.30 | 1.20 |
| Deaths | Brunei | 38.09(41.76-33.66) | 27.83 | 48.15 | 1.73 | 548.57(639.91-474.21) | 25.60 | 39.10 | 1.53 | 0.61 | 0.48 | 0.73 |
| Deaths | Bulgaria | 23.63(24.36-22.94) | 6.72 | 42.76 | 6.37 | 274.60(318.67-230.71) | 10.25 | 47.62 | 4.64 | 0.30 | 0.05 | 0.55 |
| Deaths | Burkina Faso | 8.39(9.92-6.52) | 4.61 | 12.42 | 2.69 | 94.07(119.59-73.50) | 4.33 | 12.26 | 2.84 | 1.98 | 1.81 | 2.16 |
| Deaths | Burundi | 9.89(11.91-7.68) | 4.08 | 17.76 | 4.36 | 70.49(92.65-52.26) | 3.17 | 12.16 | 3.84 | -0.51 | -0.68 | -0.34 |
| Deaths | Cambodia | 24.24(31.44-19.66) | 10.93 | 42.42 | 3.88 | 221.21(276.77-180.63) | 9.65 | 37.35 | 3.87 | -1.44 | -1.61 | -1.28 |
| Deaths | Cameroon | 11.01(13.38-8.73) | 5.11 | 17.19 | 3.36 | 114.41(175.31-72.15) | 5.27 | 17.88 | 3.39 | -0.69 | -0.79 | -0.59 |
| Deaths | Canada | 44.09(44.98-43.20) | 26.25 | 67.90 | 2.59 | 570.62(636.88-508.88) | 26.66 | 37.32 | 1.40 | 0.07 | 0.03 | 0.12 |
| Deaths | Cape Verde | 8.60(9.46-7.74) | 5.95 | 12.56 | 2.11 | 164.83(187.90-141.06) | 7.67 | 17.18 | 2.24 | -0.58 | -0.79 | -0.36 |
| Deaths | Central African Republic | 12.90(17.52-9.66) | 5.40 | 21.26 | 3.94 | 108.90(151.99-75.07) | 4.61 | 18.64 | 4.04 | 0.77 | 0.50 | 1.03 |
| Deaths | Chad | 8.87(11.53-6.87) | 4.82 | 13.14 | 2.72 | 127.61(176.02-86.53) | 5.92 | 16.89 | 2.85 | -0.59 | -0.71 | -0.46 |
| Deaths | Chile | 17.49(18.05-16.92) | 9.21 | 27.75 | 3.01 | 235.84(275.30-199.89) | 11.71 | 20.18 | 1.72 | 0.87 | 0.74 | 0.99 |
| Deaths | China | 28.30(30.19-27.06) | 17.55 | 40.47 | 2.31 | 451.83(478.87-426.09) | 21.98 | 51.92 | 2.36 | 0.82 | 0.74 | 0.90 |
| Deaths | Colombia | 13.97(14.38-13.55) | 9.32 | 18.96 | 2.03 | 166.64(195.45-141.21) | 8.00 | 13.20 | 1.65 | 0.09 | -0.06 | 0.24 |
| Deaths | Comoros | 11.23(13.77-8.92) | 6.40 | 16.19 | 2.53 | 110.11(151.20-75.27) | 4.92 | 13.05 | 2.65 | -1.12 | -1.20 | -1.03 |
| Deaths | Republic of Congo | 15.18(17.94-11.78) | 7.24 | 25.46 | 3.52 | 176.96(299.67-107.68) | 7.60 | 18.27 | 2.40 | -1.16 | -1.21 | -1.12 |
| Deaths | Costa Rica | 11.37(11.81-10.96) | 5.98 | 17.19 | 2.87 | 108.59(123.06-96.33) | 5.28 | 14.57 | 2.76 | -0.94 | -1.11 | -0.76 |
| Deaths | Ivory Coast | 7.74(8.94-6.67) | 3.73 | 11.23 | 3.01 | 72.57(98.50-53.23) | 3.37 | 9.91 | 2.94 | 1.87 | 1.65 | 2.09 |
| Deaths | Croatia | 36.68(37.83-35.58) | 9.93 | 74.78 | 7.53 | 334.32(371.42-299.86) | 13.94 | 54.26 | 3.89 | 1.25 | 1.05 | 1.45 |
| Deaths | Cuba | 31.69(32.58-30.89) | 17.21 | 46.82 | 2.72 | 481.94(560.77-410.38) | 21.63 | 43.10 | 1.99 | 0.90 | 0.54 | 1.26 |
| Deaths | Cyprus | 20.21(22.64-17.56) | 6.83 | 36.16 | 5.29 | 179.00(205.86-154.03) | 7.70 | 37.86 | 4.92 | 0.70 | 0.50 | 0.89 |
| Deaths | Czech Republic | 45.42(46.52-44.32) | 11.64 | 92.49 | 7.95 | 344.68(380.36-310.36) | 15.27 | 42.44 | 2.78 | -0.83 | -1.12 | -0.53 |
| Deaths | Democratic Republic of the Congo | 10.37(13.11-8.37) | 4.96 | 16.23 | 3.27 | 99.44(128.07-75.37) | 4.37 | 14.23 | 3.25 | -0.30 | -0.58 | -0.03 |
| Deaths | Denmark | 42.32(43.30-41.38) | 27.61 | 62.34 | 2.26 | 665.63(742.25-595.10) | 31.54 | 40.40 | 1.28 | -1.04 | -1.30 | -0.77 |
| Deaths | Djibouti | 10.84(14.17-8.11) | 5.92 | 15.92 | 2.69 | 110.97(158.61-75.99) | 4.91 | 15.82 | 3.22 | 1.39 | 1.28 | 1.50 |
| Deaths | Dominica | 12.33(13.17-11.56) | 6.07 | 21.32 | 3.51 | 189.89(214.43-167.04) | 8.56 | 19.72 | 2.30 | 1.00 | 0.80 | 1.21 |
| Deaths | Dominican Republic | 9.74(10.58-8.89) | 6.71 | 12.85 | 1.92 | 209.86(275.19-161.39) | 9.12 | 17.53 | 1.92 | 1.13 | 0.93 | 1.34 |
| Deaths | Ecuador | 7.99(8.29-7.71) | 4.91 | 11.27 | 2.29 | 153.86(179.98-131.65) | 7.34 | 10.81 | 1.47 | 0.82 | 0.73 | 0.90 |
| Deaths | Egypt | 5.92(6.39-5.52) | 2.94 | 8.71 | 2.96 | 100.37(119.99-82.84) | 3.87 | 10.70 | 2.77 | 1.26 | 1.09 | 1.42 |
| Deaths | El Salvador | 6.80(7.25-6.40) | 4.69 | 9.26 | 1.97 | 145.97(184.30-113.55) | 6.72 | 12.62 | 1.88 | 2.32 | 1.97 | 2.67 |
| Deaths | Equatorial Guinea | 11.75(17.15-7.87) | 4.58 | 20.59 | 4.49 | 152.21(267.67-82.11) | 6.68 | 17.32 | 2.59 | -0.21 | -0.41 | -0.02 |
| Deaths | Eritrea | 10.83(13.44-8.66) | 4.51 | 20.56 | 4.56 | 111.68(141.98-84.05) | 4.96 | 16.93 | 3.41 | 0.07 | -0.12 | 0.25 |
| Deaths | Estonia | 36.12(37.20-35.03) | 9.23 | 81.78 | 8.86 | 222.66(275.47-173.42) | 9.92 | 49.21 | 4.96 | 0.32 | 0.06 | 0.59 |
| Deaths | Ethiopia | 13.19(16.31-10.13) | 3.65 | 23.29 | 6.38 | 87.03(113.74-58.44) | 3.80 | 13.12 | 3.45 | -0.27 | -0.46 | -0.09 |
| Deaths | Micronesia | 25.02(30.25-21.45) | 14.06 | 37.35 | 2.66 | 304.00(385.18-242.80) | 14.04 | 38.86 | 2.77 | 1.15 | 1.01 | 1.29 |
| Deaths | Fiji | 8.45(10.04-7.15) | 5.66 | 11.38 | 2.01 | 114.51(145.03-90.28) | 5.17 | 12.02 | 2.32 | 3.56 | 3.23 | 3.89 |
| Deaths | Finland | 27.69(28.38-26.99) | 9.50 | 56.89 | 5.99 | 261.58(302.82-225.46) | 12.33 | 29.10 | 2.36 | -0.11 | -0.33 | 0.12 |
| Deaths | France | 30.35(31.32-29.57) | 7.28 | 60.65 | 8.34 | 389.48(456.97-325.44) | 15.37 | 46.43 | 3.02 | 0.44 | 0.36 | 0.51 |
| Deaths | Gabon | 14.47(18.27-9.48) | 6.08 | 25.51 | 4.20 | 142.67(229.53-91.33) | 6.30 | 22.55 | 3.58 | 0.63 | 0.18 | 1.08 |
| Deaths | Georgia | 19.63(20.77-18.51) | 6.18 | 39.44 | 6.38 | 163.29(181.57-145.78) | 6.57 | 51.43 | 7.82 | 1.95 | 1.88 | 2.01 |
| Deaths | Germany | 30.84(31.59-30.09) | 10.66 | 62.13 | 5.83 | 433.91(517.15-360.93) | 18.28 | 40.24 | 2.20 | -0.02 | -0.09 | 0.05 |
| Deaths | Ghana | 6.57(7.82-5.60) | 3.50 | 9.86 | 2.81 | 80.94(102.92-62.86) | 3.66 | 12.00 | 3.27 | 1.60 | 1.35 | 1.84 |
| Deaths | Greece | 34.32(35.42-33.35) | 9.63 | 63.87 | 6.64 | 318.71(363.04-279.77) | 13.50 | 55.82 | 4.13 | -1.74 | -1.92 | -1.56 |
| Deaths | Greenland | 112.91(122.06-103.73) | 84.59 | 153.97 | 1.82 | 1244.05(1421.53-1080.08) | 58.23 | 94.30 | 1.62 | 1.59 | 1.13 | 2.06 |
| Deaths | Grenada | 12.37(13.20-11.61) | 6.45 | 20.85 | 3.23 | 206.72(232.59-183.23) | 9.24 | 15.81 | 1.71 | 0.74 | 0.65 | 0.84 |
| Deaths | Guam | 36.49(39.91-33.22) | 17.26 | 59.05 | 3.42 | 546.68(629.06-470.87) | 24.64 | 51.61 | 2.09 | 1.74 | 1.59 | 1.88 |
| Deaths | Guatemala | 7.70(8.07-7.37) | 6.08 | 9.36 | 1.54 | 93.79(109.62-80.06) | 4.12 | 9.49 | 2.30 | -2.18 | -2.50 | -1.85 |
| Deaths | Guinea | 8.81(9.91-7.76) | 4.40 | 13.14 | 2.98 | 93.44(137.40-66.39) | 4.28 | 15.28 | 3.57 | -0.17 | -0.29 | -0.04 |
| Deaths | Guinea-Bissau | 13.43(16.36-10.54) | 5.05 | 21.75 | 4.31 | 113.17(153.49-82.51) | 5.11 | 17.66 | 3.45 | 0.04 | -0.05 | 0.13 |
| Deaths | Guyana | 6.66(7.06-6.27) | 3.85 | 9.77 | 2.53 | 130.10(154.15-108.22) | 5.34 | 10.00 | 1.87 | 1.41 | 1.11 | 1.72 |
| Deaths | Haiti | 16.49(22.06-13.24) | 7.46 | 25.83 | 3.46 | 155.28(209.83-107.03) | 6.82 | 19.87 | 2.91 | -0.45 | -0.49 | -0.41 |
| Deaths | Honduras | 7.62(8.60-6.70) | 4.56 | 10.79 | 2.37 | 126.15(165.85-91.39) | 5.50 | 10.90 | 1.98 | 0.75 | 0.64 | 0.85 |
| Deaths | Hungary | 45.90(47.16-44.69) | 17.07 | 85.67 | 5.02 | 718.98(787.18-655.06) | 28.53 | 68.99 | 2.42 | 2.32 | 1.69 | 2.96 |
| Deaths | Iceland | 32.40(33.89-30.93) | 26.82 | 39.22 | 1.46 | 501.42(549.16-457.23) | 23.03 | 31.17 | 1.35 | -0.87 | -1.09 | -0.65 |
| Deaths | India | 7.29(8.01-6.60) | 2.98 | 11.49 | 3.86 | 103.01(118.38-93.41) | 4.44 | 11.94 | 2.69 | 1.14 | 0.87 | 1.41 |
| Deaths | Indonesia | 16.07(18.63-13.95) | 8.20 | 24.76 | 3.02 | 192.66(277.32-144.04) | 8.65 | 30.50 | 3.53 | -0.40 | -0.51 | -0.30 |
| Deaths | Iran | 10.26(11.38-9.54) | 5.11 | 15.33 | 3.00 | 145.07(150.90-139.22) | 6.88 | 15.72 | 2.28 | 1.31 | 0.92 | 1.69 |
| Deaths | Iraq | 17.51(20.88-14.59) | 6.76 | 28.73 | 4.25 | 125.84(142.55-109.62) | 5.69 | 13.73 | 2.41 | -0.94 | -1.11 | -0.78 |
| Deaths | Ireland | 37.30(38.38-36.27) | 22.02 | 56.74 | 2.58 | 465.94(531.82-403.67) | 23.01 | 32.80 | 1.43 | -0.09 | -0.25 | 0.08 |
| Deaths | Israel | 20.95(21.69-20.23) | 12.17 | 31.35 | 2.58 | 249.85(280.75-223.92) | 12.12 | 28.14 | 2.32 | -0.08 | -0.22 | 0.06 |
| Deaths | Italy | 35.00(35.89-34.19) | 10.40 | 67.17 | 6.46 | 274.73(305.77-242.29) | 12.25 | 37.90 | 3.09 | 0.80 | 0.69 | 0.90 |
| Deaths | Jamaica | 14.46(15.41-13.55) | 6.04 | 24.11 | 3.99 | 171.50(215.36-135.88) | 7.39 | 32.46 | 4.39 | -0.46 | -0.56 | -0.37 |
| Deaths | Japan | 22.11(22.32-21.87) | 10.54 | 38.78 | 3.68 | 186.54(199.72-171.81) | 10.06 | 33.58 | 3.34 | 0.71 | 0.53 | 0.89 |
| Deaths | Jordan | 12.59(14.68-9.75) | 4.14 | 20.86 | 5.04 | 121.32(153.17-92.99) | 5.68 | 19.14 | 3.37 | -0.64 | -0.75 | -0.53 |
| Deaths | Kazakhstan | 35.78(36.94-34.63) | 10.92 | 75.11 | 6.88 | 152.43(173.67-129.28) | 6.44 | 37.18 | 5.78 | 0.78 | 0.54 | 1.03 |
| Deaths | Kenya | 6.81(9.44-5.63) | 3.38 | 10.23 | 3.02 | 61.96(77.42-42.09) | 2.89 | 10.88 | 3.76 | -2.50 | -2.62 | -2.38 |
| Deaths | Kiribati | 16.28(17.91-14.78) | 10.46 | 24.17 | 2.31 | 229.36(298.88-167.67) | 10.31 | 25.24 | 2.45 | -0.87 | -0.97 | -0.77 |
| Deaths | Kuwait | 10.64(11.35-9.95) | 5.63 | 14.04 | 2.49 | 68.26(83.12-53.43) | 3.15 | 9.72 | 3.09 | 0.10 | -0.18 | 0.39 |
| Deaths | Kyrgyzstan | 22.87(24.29-21.53) | 7.39 | 45.68 | 6.18 | 117.15(133.73-100.87) | 4.85 | 19.84 | 4.09 | -2.43 | -2.80 | -2.06 |
| Deaths | Laos | 25.98(32.13-20.57) | 12.26 | 41.32 | 3.37 | 272.42(352.30-209.89) | 11.65 | 32.98 | 2.83 | -1.89 | -2.17 | -1.60 |
| Deaths | Latvia | 32.61(33.57-31.66) | 8.43 | 73.35 | 8.70 | 186.68(232.51-145.42) | 8.09 | 51.61 | 6.38 | -0.44 | -0.51 | -0.38 |
| Deaths | Lebanon | 26.15(30.36-21.96) | 10.48 | 45.19 | 4.31 | 386.63(460.75-312.12) | 16.86 | 30.15 | 1.79 | 0.01 | -0.21 | 0.24 |
| Deaths | Lesotho | 12.94(18.09-10.50) | 4.65 | 24.09 | 5.18 | 145.25(225.05-93.80) | 6.47 | 31.29 | 4.84 | 1.67 | 1.58 | 1.76 |
| Deaths | Liberia | 8.82(11.79-7.30) | 4.15 | 12.61 | 3.04 | 85.81(109.92-66.39) | 3.96 | 11.58 | 2.92 | 1.55 | 0.99 | 2.12 |
| Deaths | Libya | 21.94(26.57-18.06) | 4.28 | 38.12 | 8.90 | 118.68(145.88-92.94) | 5.23 | 37.09 | 7.09 | 0.16 | -0.24 | 0.56 |
| Deaths | Lithuania | 31.74(32.62-30.89) | 7.46 | 69.43 | 9.31 | 172.54(198.31-146.31) | 7.42 | 52.43 | 7.06 | 0.92 | 0.61 | 1.22 |
| Deaths | Luxembourg | 37.91(39.55-36.24) | 11.54 | 75.63 | 6.55 | 427.67(522.56-345.02) | 18.94 | 42.15 | 2.22 | 0.03 | -0.18 | 0.24 |
| Deaths | Macedonia | 23.00(24.40-21.66) | 6.65 | 40.90 | 6.15 | 271.58(315.31-230.78) | 9.65 | 52.07 | 5.39 | 1.62 | 1.50 | 1.74 |
| Deaths | Madagascar | 8.53(9.67-7.41) | 4.23 | 12.27 | 2.90 | 81.69(108.71-57.47) | 3.51 | 11.52 | 3.29 | 1.69 | 1.52 | 1.86 |
| Deaths | Malawi | 6.34(7.61-4.35) | 3.49 | 9.46 | 2.71 | 62.32(80.98-48.31) | 2.86 | 9.72 | 3.40 | -0.95 | -1.07 | -0.83 |
| Deaths | Malaysia | 19.84(21.85-18.11) | 9.24 | 31.64 | 3.42 | 225.50(282.50-185.71) | 11.10 | 28.73 | 2.59 | -1.16 | -1.39 | -0.92 |
| Deaths | Maldives | 13.83(17.26-11.20) | 3.57 | 20.27 | 5.67 | 59.29(69.05-50.55) | 2.72 | 11.33 | 4.16 | 0.70 | 0.43 | 0.98 |
| Deaths | Mali | 7.43(8.51-6.54) | 4.02 | 10.75 | 2.67 | 87.11(110.23-67.89) | 4.02 | 9.89 | 2.46 | -1.60 | -1.77 | -1.44 |
| Deaths | Malta | 24.62(25.81-23.46) | 5.93 | 48.66 | 8.21 | 190.34(212.63-169.70) | 8.41 | 38.22 | 4.55 | -0.18 | -0.24 | -0.11 |
| Deaths | Marshall Islands | 25.44(34.92-15.47) | 8.17 | 44.27 | 5.42 | 246.29(438.44-140.27) | 10.75 | 45.90 | 4.27 | 1.46 | 1.37 | 1.55 |
| Deaths | Mauritania | 10.37(12.39-8.58) | 5.86 | 15.51 | 2.65 | 127.15(173.32-95.42) | 5.89 | 13.64 | 2.32 | 0.96 | 0.81 | 1.11 |
| Deaths | Mauritius | 13.26(13.94-12.53) | 5.58 | 23.67 | 4.24 | 134.41(153.83-114.84) | 5.83 | 19.34 | 3.32 | -0.07 | -0.25 | 0.11 |
| Deaths | Mexico | 13.43(13.62-13.24) | 7.75 | 19.77 | 2.55 | 112.29(116.91-108.26) | 5.34 | 12.55 | 2.35 | 0.16 | -0.05 | 0.38 |
| Deaths | Moldova | 25.19(26.14-24.42) | 8.03 | 49.10 | 6.12 | 152.37(171.06-132.61) | 6.02 | 35.30 | 5.86 | -1.73 | -1.85 | -1.62 |
| Deaths | Mongolia | 38.22(41.83-34.98) | 17.68 | 64.95 | 3.67 | 217.97(259.38-184.19) | 11.32 | 49.64 | 4.38 | -0.22 | -0.27 | -0.17 |
| Deaths | Montenegro | 41.14(44.43-38.11) | 16.82 | 72.58 | 4.32 | 517.50(618.99-416.44) | 20.82 | 71.60 | 3.44 | -0.78 | -1.08 | -0.48 |
| Deaths | Morocco | 13.38(15.69-11.49) | 3.27 | 23.51 | 7.19 | 79.57(98.21-62.00) | 3.57 | 24.33 | 6.81 | -2.94 | -3.37 | -2.51 |
| Deaths | Mozambique | 9.35(10.79-8.13) | 3.43 | 16.05 | 4.68 | 75.10(97.38-55.38) | 3.54 | 19.30 | 5.45 | 0.94 | 0.83 | 1.05 |
| Deaths | Myanmar | 22.87(29.27-17.85) | 13.54 | 33.52 | 2.48 | 277.67(347.82-218.01) | 11.69 | 27.72 | 2.37 | 0.31 | 0.21 | 0.40 |
| Deaths | Namibia | 8.91(9.93-7.96) | 4.70 | 14.15 | 3.01 | 86.72(117.38-63.18) | 3.94 | 12.73 | 3.23 | 0.02 | -0.31 | 0.34 |
| Deaths | Nepal | 11.59(17.58-6.90) | 8.33 | 14.67 | 1.76 | 191.24(311.39-75.00) | 8.55 | 14.12 | 1.65 | -0.48 | -0.66 | -0.31 |
| Deaths | Netherlands | 45.55(46.63-44.48) | 13.06 | 91.82 | 7.03 | 654.97(717.74-591.55) | 28.27 | 46.60 | 1.65 | -1.38 | -1.89 | -0.88 |
| Deaths | New Zealand | 32.83(33.93-31.80) | 19.92 | 50.23 | 2.52 | 459.50(500.80-417.25) | 20.87 | 26.90 | 1.29 | 0.09 | 0.03 | 0.15 |
| Deaths | Nicaragua | 6.82(7.49-6.21) | 4.17 | 9.77 | 2.34 | 92.06(109.84-75.59) | 4.25 | 8.62 | 2.03 | 2.47 | 2.05 | 2.89 |
| Deaths | Niger | 8.60(11.39-6.41) | 4.36 | 12.50 | 2.87 | 95.44(131.83-59.65) | 4.37 | 12.37 | 2.83 | -0.15 | -0.26 | -0.03 |
| Deaths | Nigeria | 8.63(11.17-6.48) | 3.95 | 13.45 | 3.40 | 97.68(142.52-63.16) | 4.72 | 12.21 | 2.59 | 0.09 | -0.07 | 0.25 |
| Deaths | North Korea | 23.60(27.78-19.65) | 12.82 | 43.29 | 3.38 | 267.83(366.61-195.07) | 11.35 | 43.41 | 3.82 | -0.24 | -0.34 | -0.14 |
| Deaths | Northern Mariana Islands | 39.58(49.00-32.77) | 18.34 | 57.59 | 3.14 | 312.41(365.65-268.57) | 14.94 | 48.97 | 3.28 | 1.04 | 0.80 | 1.28 |
| Deaths | Norway | 23.88(24.29-23.55) | 12.60 | 38.46 | 3.05 | 399.77(420.10-380.78) | 18.45 | 27.26 | 1.48 | -0.74 | -0.86 | -0.62 |
| Deaths | Oman | 8.20(10.15-6.58) | 3.17 | 13.01 | 4.10 | 81.71(99.96-64.19) | 3.68 | 9.66 | 2.62 | -0.76 | -0.83 | -0.69 |
| Deaths | Pakistan | 12.41(15.00-10.95) | 4.97 | 18.49 | 3.72 | 143.01(183.56-106.13) | 6.14 | 21.10 | 3.43 | 1.13 | 0.74 | 1.52 |
| Deaths | Palestine | 17.41(21.29-14.02) | 6.76 | 29.57 | 4.37 | 173.84(196.94-149.91) | 7.69 | 22.66 | 2.95 | 0.45 | 0.34 | 0.55 |
| Deaths | Panama | 13.25(13.91-12.64) | 6.12 | 20.32 | 3.32 | 138.44(156.41-120.61) | 6.39 | 13.07 | 2.05 | 0.74 | 0.58 | 0.89 |
| Deaths | Papua New Guinea | 25.30(34.29-20.65) | 12.33 | 37.26 | 3.02 | 321.33(419.90-239.61) | 13.85 | 39.69 | 2.87 | 0.33 | 0.19 | 0.47 |
| Deaths | Paraguay | 11.21(12.21-10.12) | 6.48 | 16.63 | 2.57 | 158.18(203.91-122.75) | 7.16 | 23.66 | 3.31 | -0.20 | -0.33 | -0.08 |
| Deaths | Peru | 12.86(14.17-11.56) | 8.97 | 17.07 | 1.90 | 179.25(218.84-142.40) | 8.15 | 11.03 | 1.35 | 0.22 | 0.20 | 0.24 |
| Deaths | Philippines | 16.11(17.10-15.09) | 8.21 | 24.69 | 3.01 | 234.36(285.84-189.23) | 10.38 | 28.60 | 2.76 | 0.33 | 0.23 | 0.43 |
| Deaths | Poland | 40.65(41.61-39.68) | 12.47 | 79.38 | 6.36 | 484.51(547.43-419.87) | 20.30 | 64.19 | 3.16 | -0.61 | -0.85 | -0.36 |
| Deaths | Portugal | 18.76(19.30-18.17) | 6.59 | 34.84 | 5.29 | 199.33(234.53-163.04) | 8.34 | 33.50 | 4.01 | 0.47 | 0.35 | 0.59 |
| Deaths | Puerto Rico | 15.25(15.79-14.68) | 8.74 | 22.90 | 2.62 | 153.55(172.44-137.37) | 7.33 | 15.18 | 2.07 | 1.80 | 1.64 | 1.96 |
| Deaths | Qatar | 20.72(24.62-17.29) | 7.44 | 30.96 | 4.16 | 131.33(161.60-103.75) | 6.12 | 16.59 | 2.71 | 1.20 | 1.07 | 1.32 |
| Deaths | Romania | 23.02(23.71-22.32) | 7.22 | 41.41 | 5.74 | 294.53(325.62-261.94) | 11.56 | 51.69 | 4.47 | -0.93 | -1.04 | -0.82 |
| Deaths | Russia | 31.33(31.81-30.74) | 8.08 | 73.55 | 9.10 | 152.25(157.42-145.44) | 6.38 | 45.86 | 7.19 | -0.79 | -0.96 | -0.62 |
| Deaths | Rwanda | 10.15(12.70-7.40) | 4.02 | 17.41 | 4.33 | 82.13(111.32-55.92) | 3.69 | 13.32 | 3.61 | -0.04 | -0.21 | 0.13 |
| Deaths | Saint Lucia | 12.32(13.12-11.56) | 7.03 | 19.34 | 2.75 | 171.53(194.68-151.76) | 7.49 | 14.73 | 1.97 | 1.63 | 1.54 | 1.71 |
| Deaths | Saint Vincent | 8.82(9.46-8.16) | 5.63 | 12.96 | 2.30 | 156.58(177.49-137.72) | 6.51 | 12.89 | 1.98 | -0.95 | -1.15 | -0.75 |
| Deaths | Grenadines | 8.82(9.46-8.16) | 5.63 | 12.96 | 2.30 | 156.58(177.49-137.72) | 6.51 | 12.89 | 1.98 | -0.68 | -0.87 | -0.50 |
| Deaths | Samoa | 8.04(9.25-6.85) | 4.16 | 12.05 | 2.90 | 99.74(127.08-76.60) | 4.41 | 11.86 | 2.69 | 0.23 | -0.06 | 0.52 |
| Deaths | Sao Tome and Principe | 12.23(14.03-10.92) | 5.10 | 21.21 | 4.16 | 150.36(260.67-88.82) | 6.42 | 26.55 | 4.14 | 0.74 | 0.65 | 0.84 |
| Deaths | Saudi Arabia | 8.08(10.04-6.46) | 2.38 | 12.46 | 5.24 | 75.70(91.68-64.26) | 3.26 | 14.47 | 4.43 | 0.15 | 0.07 | 0.23 |
| Deaths | Senegal | 12.12(15.46-9.99) | 4.92 | 19.11 | 3.88 | 110.80(140.36-86.99) | 5.09 | 18.66 | 3.67 | 0.80 | 0.70 | 0.90 |
| Deaths | Serbia | 39.11(42.89-36.36) | 13.90 | 68.68 | 4.94 | 573.46(640.22-504.12) | 22.45 | 62.30 | 2.77 | 0.95 | 0.83 | 1.07 |
| Deaths | Seychelles | 15.56(16.96-14.15) | 5.72 | 28.68 | 5.01 | 149.72(170.98-131.33) | 6.89 | 25.56 | 3.71 | 0.16 | 0.00 | 0.33 |
| Deaths | Sierra Leone | 10.65(14.63-7.86) | 4.98 | 15.91 | 3.19 | 109.73(140.01-84.90) | 5.07 | 15.60 | 3.08 | 2.51 | 2.15 | 2.87 |
| Deaths | Singapore | 36.49(37.58-35.46) | 19.11 | 57.90 | 3.03 | 210.50(241.06-184.70) | 10.39 | 28.88 | 2.78 | 0.24 | 0.16 | 0.32 |
| Deaths | Slovakia | 36.83(38.28-35.28) | 7.98 | 74.75 | 9.36 | 265.05(297.45-233.82) | 11.12 | 45.23 | 4.07 | 0.21 | 0.00 | 0.43 |
| Deaths | Slovenia | 33.86(34.75-32.92) | 10.29 | 69.25 | 6.73 | 346.66(396.60-301.81) | 14.57 | 43.64 | 2.99 | -2.58 | -2.72 | -2.44 |
| Deaths | Solomon Islands | 23.58(32.17-18.58) | 10.86 | 33.17 | 3.05 | 242.68(293.71-192.73) | 11.00 | 31.35 | 2.85 | 1.68 | 1.48 | 1.88 |
| Deaths | Somalia | 9.18(12.25-6.28) | 4.14 | 14.25 | 3.44 | 84.91(118.46-54.74) | 3.72 | 13.55 | 3.64 | 1.40 | 1.15 | 1.65 |
| Deaths | South Africa | 17.98(22.51-16.25) | 9.84 | 28.83 | 2.93 | 201.41(213.08-190.50) | 9.02 | 27.08 | 3.00 | -0.23 | -0.31 | -0.14 |
| Deaths | South Korea | 20.60(21.18-20.03) | 9.30 | 37.64 | 4.05 | 210.62(238.04-184.22) | 11.28 | 39.84 | 3.53 | -0.63 | -0.79 | -0.47 |
| Deaths | South Sudan | 10.70(14.69-7.47) | 5.01 | 15.35 | 3.06 | 101.75(152.41-61.86) | 4.48 | 15.54 | 3.47 | -0.71 | -1.10 | -0.32 |
| Deaths | Spain | 27.48(28.22-26.78) | 5.28 | 56.13 | 10.62 | 248.46(289.46-208.95) | 9.71 | 43.05 | 4.43 | -0.77 | -1.25 | -0.30 |
| Deaths | Sri Lanka | 7.55(8.19-6.96) | 3.71 | 11.32 | 3.05 | 81.90(107.75-61.06) | 3.60 | 11.54 | 3.20 | -0.59 | -0.75 | -0.44 |
| Deaths | Sudan | 8.78(13.33-6.53) | 4.02 | 13.07 | 3.25 | 92.46(121.82-66.53) | 4.07 | 11.86 | 2.92 | 3.41 | 3.17 | 3.64 |
| Deaths | Suriname | 12.55(13.55-11.64) | 7.47 | 18.35 | 2.45 | 219.78(261.76-183.08) | 9.49 | 20.50 | 2.16 | -0.13 | -0.46 | 0.21 |
| Deaths | Swaziland | 17.06(21.12-13.71) | 7.07 | 30.28 | 4.28 | 147.23(244.48-88.45) | 6.57 | 28.81 | 4.39 | 0.18 | 0.05 | 0.31 |
| Deaths | Sweden | 19.41(19.92-18.95) | 12.17 | 28.82 | 2.37 | 375.66(408.62-344.34) | 18.03 | 19.56 | 1.09 | 1.09 | 0.94 | 1.23 |
| Deaths | Switzerland | 28.99(29.70-28.30) | 10.27 | 54.09 | 5.27 | 336.11(374.52-299.19) | 14.85 | 27.15 | 1.83 | -0.25 | -0.68 | 0.17 |
| Deaths | Syria | 9.48(10.85-8.32) | 4.38 | 14.10 | 3.22 | 106.52(130.92-83.10) | 4.28 | 14.67 | 3.43 | 0.93 | 0.67 | 1.20 |
| Deaths | Taiwan (Province of China) | 22.69(23.25-22.14) | 14.06 | 30.52 | 2.17 | 384.50(422.41-350.97) | 18.51 | 38.13 | 2.06 | 0.97 | 0.58 | 1.36 |
| Deaths | Tajikistan | 12.69(13.38-12.03) | 6.54 | 20.38 | 3.12 | 117.13(136.64-99.03) | 4.45 | 11.70 | 2.63 | -0.37 | -0.66 | -0.07 |
| Deaths | Tanzania | 7.71(9.12-6.30) | 3.72 | 11.80 | 3.18 | 82.78(104.74-65.08) | 3.76 | 11.53 | 3.07 | 0.60 | 0.41 | 0.80 |
| Deaths | Thailand | 29.34(31.23-27.44) | 21.50 | 38.58 | 1.79 | 261.07(304.37-222.52) | 11.99 | 30.36 | 2.53 | -0.78 | -1.47 | -0.08 |
| Deaths | Bahamas | 15.58(16.73-14.49) | 6.70 | 27.27 | 4.07 | 181.48(209.80-153.65) | 7.76 | 20.22 | 2.61 | -0.30 | -0.47 | -0.14 |
| Deaths | Gambia | 7.97(9.51-6.56) | 3.89 | 11.72 | 3.01 | 92.62(126.96-67.60) | 4.23 | 12.06 | 2.85 | -3.09 | -3.29 | -2.89 |
| Deaths | Timor-Leste | 16.22(21.58-12.85) | 9.35 | 22.85 | 2.44 | 216.75(274.86-164.86) | 9.52 | 28.28 | 2.97 | 0.08 | -0.16 | 0.32 |
| Deaths | Togo | 9.43(10.79-8.11) | 4.76 | 14.45 | 3.04 | 96.99(126.22-73.34) | 4.44 | 16.06 | 3.62 | -0.24 | -0.49 | 0.01 |
| Deaths | Tonga | 30.01(33.14-27.22) | 13.28 | 47.63 | 3.59 | 237.88(306.42-188.73) | 11.64 | 45.82 | 3.94 | -0.48 | -0.53 | -0.43 |
| Deaths | Trinidad | 11.25(11.84-10.66) | 5.16 | 18.16 | 3.52 | 118.29(154.84-86.76) | 5.10 | 15.28 | 3.00 | -0.53 | -0.62 | -0.45 |
| Deaths | Tobago | 11.25(11.84-10.66) | 5.16 | 18.16 | 3.52 | 118.29(154.84-86.76) | 5.10 | 15.28 | 3.00 | -0.24 | -0.49 | 0.01 |
| Deaths | Tunisia | 17.92(20.20-15.66) | 2.83 | 31.63 | 11.16 | 78.03(103.36-57.36) | 3.63 | 32.17 | 8.86 | 0.74 | 0.60 | 0.88 |
| Deaths | Turkey | 38.38(42.65-33.41) | 9.46 | 70.58 | 7.46 | 196.75(225.73-171.01) | 7.90 | 59.37 | 7.51 | -1.14 | -1.34 | -0.94 |
| Deaths | Turkmenistan | 13.30(13.89-12.74) | 5.17 | 24.19 | 4.68 | 111.81(129.27-95.01) | 4.61 | 16.74 | 3.63 | -0.94 | -1.37 | -0.51 |
| Deaths | Uganda | 5.75(6.89-4.80) | 3.80 | 7.53 | 1.98 | 83.50(105.21-66.05) | 3.70 | 10.83 | 2.93 | -0.59 | -0.77 | -0.42 |
| Deaths | Ukraine | 31.91(32.97-30.90) | 8.97 | 69.66 | 7.76 | 148.36(169.14-127.31) | 5.65 | 43.66 | 7.72 | -0.31 | -0.37 | -0.25 |
| Deaths | United Arab Emirates | 14.85(18.26-11.96) | 6.72 | 21.00 | 3.13 | 165.92(209.09-125.72) | 7.76 | 22.38 | 2.88 | -2.02 | -2.32 | -1.73 |
| Deaths | UK | 43.64(44.03-43.20) | 25.20 | 70.71 | 2.81 | 515.68(528.77-503.22) | 25.54 | 36.52 | 1.43 | 0.43 | 0.09 | 0.77 |
| Deaths | USA | 46.95(47.37-46.52) | 30.30 | 69.94 | 2.31 | 583.02(611.53-557.31) | 27.99 | 42.32 | 1.51 | 2.05 | 1.88 | 2.22 |
| Deaths | Uruguay | 37.10(38.36-35.84) | 8.77 | 72.72 | 8.29 | 340.88(402.25-282.87) | 14.02 | 51.42 | 3.67 | -1.11 | -1.32 | -0.89 |
| Deaths | Uzbekistan | 14.04(14.54-13.60) | 6.44 | 24.37 | 3.79 | 116.28(137.60-96.15) | 4.55 | 19.05 | 4.19 | -1.51 | -1.77 | -1.24 |
| Deaths | Vanuatu | 25.72(35.10-20.12) | 12.60 | 36.15 | 2.87 | 289.86(376.32-216.97) | 12.70 | 37.09 | 2.92 | 0.02 | -0.09 | 0.12 |
| Deaths | Venezuela | 16.95(17.52-16.35) | 11.26 | 23.48 | 2.09 | 274.85(333.99-218.75) | 12.24 | 23.03 | 1.88 | 0.33 | 0.24 | 0.42 |
| Deaths | Vietnam | 35.11(40.26-30.61) | 18.33 | 59.09 | 3.22 | 461.24(602.71-349.45) | 21.26 | 67.72 | 3.19 | 0.52 | 0.40 | 0.65 |
| Deaths | Virgin Islands | 18.32(20.08-16.76) | 10.20 | 28.34 | 2.78 | 276.82(323.88-235.44) | 12.66 | 32.83 | 2.59 | 0.92 | 0.77 | 1.07 |
| Deaths | Yemen | 10.74(16.09-6.82) | 4.98 | 17.01 | 3.42 | 106.47(143.44-78.49) | 4.63 | 16.68 | 3.60 | 0.05 | -0.09 | 0.19 |
| Deaths | Zambia | 10.28(11.97-8.75) | 4.25 | 15.26 | 3.59 | 83.65(106.03-66.11) | 3.87 | 14.20 | 3.67 | -0.88 | -1.33 | -0.42 |
| Deaths | Zimbabwe | 12.19(13.93-10.55) | 5.23 | 19.87 | 3.80 | 143.97(190.89-105.13) | 6.14 | 16.90 | 2.75 | 1.04 | 0.30 | 1.80 |
| Incidence | Afghanistan | 13.06(22.30-7.10) | 5.66 | 18.80 | 3.32 | 440.62(747.39-311.58) | 5.76 | 18.23 | 3.17 | 0.04 | -0.08 | 0.16 |
| Incidence | Albania | 24.00(25.27-22.67) | 6.99 | 43.11 | 6.17 | 919.61(1169.22-706.12) | 7.63 | 40.32 | 5.29 | -0.37 | -0.58 | -0.16 |
| Incidence | Algeria | 9.85(11.03-8.75) | 3.14 | 16.80 | 5.35 | 305.56(348.97-262.44) | 3.22 | 13.02 | 4.04 | -0.86 | -1.00 | -0.72 |
| Incidence | American Samoa | 25.63(28.21-23.11) | 11.45 | 39.96 | 3.49 | 798.70(945.99-659.00) | 13.47 | 34.95 | 2.59 | -0.46 | -0.56 | -0.37 |
| Incidence | Andorra | 33.45(39.37-28.38) | 14.53 | 51.90 | 3.57 | 724.01(861.15-593.92) | 19.90 | 41.57 | 2.09 | -1.45 | -1.52 | -1.38 |
| Incidence | Angola | 14.07(18.11-10.63) | 5.55 | 22.44 | 4.04 | 435.29(567.40-334.09) | 4.97 | 18.10 | 3.64 | -0.98 | -1.08 | -0.88 |
| Incidence | Antigua | 8.13(8.77-7.55) | 3.86 | 13.59 | 3.52 | 224.08(253.20-200.43) | 5.49 | 9.95 | 1.81 | -1.27 | -1.38 | -1.16 |
| Incidence | Barbuda | 8.13(8.77-7.55) | 3.86 | 13.59 | 3.52 | 224.08(253.20-200.43) | 5.49 | 9.95 | 1.81 | -1.97 | -2.11 | -1.83 |
| Incidence | Argentina | 25.18(25.94-24.43) | 8.63 | 45.37 | 5.26 | 719.21(832.14-618.34) | 11.88 | 30.92 | 2.60 | -0.35 | -0.61 | -0.09 |
| Incidence | Armenia | 28.72(30.40-27.27) | 8.45 | 54.97 | 6.51 | 1296.70(1378.63-1215.01) | 8.54 | 54.28 | 6.36 | -2.63 | -2.76 | -2.51 |
| Incidence | Australia | 35.00(35.95-34.14) | 17.57 | 57.03 | 3.25 | 559.48(643.95-482.71) | 24.29 | 40.51 | 1.67 | -2.03 | -2.27 | -1.79 |
| Incidence | Austria | 28.26(29.33-27.34) | 11.46 | 53.59 | 4.68 | 713.76(783.29-656.07) | 24.47 | 42.55 | 1.74 | -0.41 | -0.89 | 0.07 |
| Incidence | Azerbaijan | 18.88(20.03-17.76) | 6.13 | 36.11 | 5.89 | 903.87(1077.85-700.52) | 7.09 | 35.99 | 5.08 | -1.07 | -1.20 | -0.93 |
| Incidence | Bahrain | 35.55(38.82-31.68) | 15.60 | 52.46 | 3.36 | 321.84(370.76-277.40) | 6.79 | 16.54 | 2.44 | -5.29 | -5.72 | -4.86 |
| Incidence | Bangladesh | 21.60(26.07-18.31) | 8.84 | 32.01 | 3.62 | 673.18(797.88-565.57) | 8.93 | 29.53 | 3.31 | -0.39 | -0.62 | -0.16 |
| Incidence | Barbados | 8.64(9.23-8.13) | 4.31 | 14.70 | 3.41 | 250.38(282.76-215.71) | 5.13 | 11.13 | 2.17 | -1.13 | -1.28 | -0.99 |
| Incidence | Belarus | 29.00(29.97-28.11) | 6.18 | 65.18 | 10.54 | 1040.95(1144.48-943.14) | 5.71 | 48.89 | 8.57 | -1.27 | -1.38 | -1.16 |
| Incidence | Belgium | 45.12(46.73-43.62) | 11.56 | 90.93 | 7.87 | 1029.48(1117.99-940.87) | 22.03 | 55.99 | 2.54 | -2.21 | -2.54 | -1.87 |
| Incidence | Belize | 9.59(10.56-8.75) | 4.87 | 14.38 | 2.95 | 402.55(443.90-366.57) | 6.65 | 16.54 | 2.49 | -2.37 | -2.47 | -2.28 |
| Incidence | Benin | 9.59(11.11-8.17) | 4.60 | 14.81 | 3.22 | 308.49(403.18-233.75) | 4.51 | 13.98 | 3.10 | 0.36 | -0.13 | 0.85 |
| Incidence | Bermuda | 29.52(31.38-27.74) | 14.63 | 49.71 | 3.40 | 696.02(768.15-630.51) | 10.93 | 35.29 | 3.23 | -0.12 | -0.21 | -0.03 |
| Incidence | Bhutan | 7.99(9.82-6.55) | 4.62 | 11.32 | 2.45 | 223.75(314.31-159.68) | 4.26 | 9.97 | 2.34 | -1.54 | -1.73 | -1.35 |
| Incidence | Bolivia | 12.10(14.73-9.79) | 7.08 | 17.87 | 2.53 | 317.60(431.37-217.59) | 8.24 | 14.46 | 1.76 | -0.55 | -0.73 | -0.37 |
| Incidence | Bosnia and Herzegovina | 33.21(34.90-31.69) | 9.72 | 64.10 | 6.60 | 1472.81(1639.04-1321.77) | 13.92 | 61.44 | 4.41 | -1.10 | -1.18 | -1.02 |
| Incidence | Botswana | 14.84(17.46-12.68) | 6.74 | 25.16 | 3.73 | 378.68(476.50-301.14) | 7.34 | 16.82 | 2.29 | -0.75 | -0.93 | -0.58 |
| Incidence | Brazil | 15.06(15.33-14.73) | 8.83 | 22.28 | 2.52 | 398.12(407.74-388.34) | 10.37 | 17.82 | 1.72 | -2.08 | -2.30 | -1.86 |
| Incidence | Brunei | 35.58(38.99-31.63) | 26.30 | 44.55 | 1.69 | 758.75(873.91-659.32) | 26.33 | 38.64 | 1.47 | -1.31 | -1.51 | -1.10 |
| Incidence | Bulgaria | 24.03(24.77-23.29) | 6.63 | 43.50 | 6.56 | 1228.94(1345.90-1112.79) | 10.43 | 49.75 | 4.77 | -0.38 | -0.61 | -0.14 |
| Incidence | Burkina Faso | 7.89(9.39-6.05) | 4.38 | 11.65 | 2.66 | 253.63(312.20-200.99) | 4.08 | 11.60 | 2.84 | 0.21 | 0.06 | 0.36 |
| Incidence | Burundi | 9.21(11.11-7.08) | 3.85 | 16.37 | 4.26 | 248.49(330.31-188.81) | 2.94 | 11.04 | 3.76 | 0.04 | -0.07 | 0.15 |
| Incidence | Cambodia | 23.36(30.08-18.82) | 10.54 | 40.74 | 3.86 | 815.11(1025.74-646.85) | 9.14 | 35.00 | 3.83 | -1.83 | -1.99 | -1.68 |
| Incidence | Cameroon | 10.38(12.64-8.24) | 4.83 | 16.18 | 3.35 | 370.59(483.48-277.66) | 4.92 | 16.61 | 3.38 | -0.81 | -0.98 | -0.64 |
| Incidence | Canada | 45.43(47.21-44.10) | 27.60 | 68.71 | 2.49 | 729.15(798.29-666.87) | 33.57 | 43.55 | 1.30 | 0.12 | 0.00 | 0.24 |
| Incidence | Cape Verde | 8.16(8.98-7.33) | 5.73 | 11.76 | 2.05 | 342.93(396.79-294.23) | 7.13 | 15.67 | 2.20 | -2.68 | -2.80 | -2.56 |
| Incidence | Central African Republic | 12.34(16.85-9.01) | 5.16 | 20.31 | 3.94 | 449.85(700.04-286.43) | 4.35 | 17.73 | 4.07 | 0.65 | 0.25 | 1.05 |
| Incidence | Chad | 8.32(10.82-6.41) | 4.54 | 12.30 | 2.71 | 351.65(476.81-259.06) | 5.52 | 15.78 | 2.86 | -0.57 | -0.71 | -0.43 |
| Incidence | Chile | 16.58(17.12-16.05) | 8.62 | 26.32 | 3.05 | 402.43(470.92-346.19) | 11.15 | 19.06 | 1.71 | 1.13 | 1.01 | 1.24 |
| Incidence | China | 27.41(29.23-26.18) | 16.97 | 38.93 | 2.29 | 1075.36(1137.68-1010.44) | 25.18 | 60.36 | 2.40 | -1.41 | -1.54 | -1.28 |
| Incidence | Colombia | 13.13(13.55-12.71) | 8.85 | 17.72 | 2.00 | 269.46(312.34-231.37) | 7.48 | 12.35 | 1.65 | 0.59 | 0.49 | 0.69 |
| Incidence | Comoros | 10.44(12.85-8.31) | 6.04 | 14.95 | 2.48 | 263.40(365.92-203.38) | 4.60 | 11.86 | 2.58 | -1.96 | -2.16 | -1.75 |
| Incidence | Republic of Congo | 14.52(17.24-11.06) | 6.89 | 24.32 | 3.53 | 407.49(516.96-309.39) | 7.20 | 17.08 | 2.37 | -1.17 | -1.39 | -0.95 |
| Incidence | Costa Rica | 10.66(11.08-10.27) | 5.61 | 16.08 | 2.86 | 290.26(320.56-261.50) | 4.97 | 14.03 | 2.83 | -1.18 | -1.45 | -0.90 |
| Incidence | Ivory Coast | 7.39(8.59-6.35) | 3.48 | 10.77 | 3.09 | 209.02(264.82-161.76) | 3.13 | 9.41 | 3.00 | -1.44 | -1.63 | -1.26 |
| Incidence | Croatia | 39.86(41.71-38.21) | 10.74 | 80.68 | 7.51 | 1217.25(1328.98-1117.68) | 18.65 | 64.07 | 3.44 | -0.26 | -0.37 | -0.15 |
| Incidence | Cuba | 29.65(30.46-28.91) | 16.22 | 43.64 | 2.69 | 900.61(1048.29-779.67) | 20.99 | 41.53 | 1.98 | 0.56 | 0.30 | 0.82 |
| Incidence | Cyprus | 19.29(21.71-16.77) | 6.56 | 34.25 | 5.22 | 776.21(881.39-679.08) | 8.72 | 45.09 | 5.17 | -3.39 | -3.48 | -3.30 |
| Incidence | Czech Republic | 45.78(47.03-44.51) | 11.55 | 92.18 | 7.98 | 899.39(980.82-823.90) | 19.29 | 51.24 | 2.66 | -0.66 | -0.81 | -0.51 |
| Incidence | Democratic Republic of the Congo | 9.75(12.34-7.84) | 4.70 | 15.28 | 3.25 | 319.65(485.10-226.94) | 4.12 | 13.35 | 3.24 | -2.15 | -2.29 | -2.00 |
| Incidence | Denmark | 45.23(46.36-44.17) | 30.76 | 64.56 | 2.10 | 783.05(853.29-718.27) | 40.97 | 48.30 | 1.18 | -0.44 | -0.55 | -0.33 |
| Incidence | Djibouti | 10.05(13.21-7.50) | 5.58 | 14.60 | 2.62 | 320.01(494.29-223.54) | 4.60 | 14.37 | 3.13 | -0.40 | -0.52 | -0.28 |
| Incidence | Dominica | 11.65(12.45-10.91) | 5.74 | 20.03 | 3.49 | 430.07(483.34-383.48) | 8.07 | 18.53 | 2.30 | 0.65 | 0.38 | 0.92 |
| Incidence | Dominican Republic | 9.23(10.04-8.44) | 6.38 | 12.14 | 1.90 | 371.19(447.44-293.67) | 8.70 | 16.32 | 1.88 | -0.44 | -0.71 | -0.17 |
| Incidence | Ecuador | 7.50(7.77-7.24) | 4.67 | 10.49 | 2.25 | 206.37(236.88-179.46) | 6.83 | 9.82 | 1.44 | 0.77 | 0.53 | 1.01 |
| Incidence | Egypt | 5.74(6.19-5.33) | 2.93 | 8.38 | 2.86 | 248.24(299.31-197.05) | 3.82 | 10.24 | 2.68 | 0.93 | 0.76 | 1.10 |
| Incidence | El Salvador | 6.47(6.89-6.09) | 4.48 | 8.78 | 1.96 | 266.48(325.92-212.06) | 6.33 | 11.89 | 1.88 | -1.17 | -1.45 | -0.89 |
| Incidence | Equatorial Guinea | 11.26(16.57-7.39) | 4.36 | 19.68 | 4.52 | 373.89(559.05-219.94) | 6.30 | 16.08 | 2.55 | -1.20 | -1.45 | -0.95 |
| Incidence | Eritrea | 10.24(12.76-8.12) | 4.25 | 19.27 | 4.53 | 380.01(490.09-283.00) | 4.62 | 15.67 | 3.39 | -2.59 | -2.89 | -2.28 |
| Incidence | Estonia | 36.11(37.22-35.01) | 9.66 | 79.62 | 8.24 | 1040.02(1241.96-885.50) | 15.07 | 48.38 | 3.21 | -2.51 | -2.74 | -2.29 |
| Incidence | Ethiopia | 12.28(15.20-9.32) | 3.43 | 21.46 | 6.25 | 258.51(296.25-225.65) | 3.60 | 11.91 | 3.31 | -0.04 | -0.14 | 0.06 |
| Incidence | Micronesia | 23.67(28.72-20.21) | 13.20 | 35.18 | 2.66 | 834.56(1104.59-644.94) | 13.08 | 36.16 | 2.76 | -2.81 | -2.94 | -2.68 |
| Incidence | Fiji | 7.91(9.38-6.71) | 5.32 | 10.61 | 1.99 | 247.63(295.76-206.04) | 4.85 | 11.07 | 2.28 | -1.00 | -1.20 | -0.79 |
| Incidence | Finland | 32.29(33.18-31.41) | 12.38 | 63.12 | 5.10 | 565.44(619.21-513.89) | 16.46 | 36.78 | 2.23 | -0.63 | -0.69 | -0.57 |
| Incidence | France | 30.19(31.25-29.33) | 7.10 | 59.60 | 8.40 | 1065.78(1160.35-973.85) | 18.43 | 51.80 | 2.81 | 0.06 | -0.05 | 0.17 |
| Incidence | Gabon | 13.77(17.45-8.91) | 5.76 | 24.09 | 4.18 | 505.59(653.99-379.35) | 5.94 | 21.09 | 3.55 | 1.67 | 0.78 | 2.56 |
| Incidence | Georgia | 19.85(20.98-18.69) | 6.13 | 39.50 | 6.44 | 1316.46(1420.96-1221.55) | 6.44 | 50.86 | 7.89 | -2.03 | -2.22 | -1.83 |
| Incidence | Germany | 35.54(36.63-34.46) | 11.78 | 71.16 | 6.04 | 868.67(995.98-748.41) | 25.86 | 52.53 | 2.03 | 0.90 | 0.77 | 1.02 |
| Incidence | Ghana | 6.19(7.38-5.26) | 3.31 | 9.26 | 2.79 | 246.90(296.11-197.12) | 3.46 | 11.24 | 3.25 | -0.65 | -0.74 | -0.56 |
| Incidence | Greece | 33.59(34.78-32.54) | 9.40 | 62.16 | 6.62 | 1206.86(1313.36-1106.62) | 15.33 | 59.88 | 3.91 | -2.09 | -2.23 | -1.95 |
| Incidence | Greenland | 105.92(114.24-97.09) | 80.14 | 141.53 | 1.77 | 1892.33(2064.53-1703.87) | 54.80 | 86.90 | 1.59 | -0.45 | -0.73 | -0.17 |
| Incidence | Grenada | 11.76(12.54-11.03) | 6.16 | 19.60 | 3.18 | 377.73(421.47-340.65) | 8.71 | 15.25 | 1.75 | 0.07 | -0.08 | 0.22 |
| Incidence | Guam | 34.20(37.34-31.09) | 16.16 | 54.50 | 3.37 | 1138.13(1283.77-988.47) | 23.43 | 48.96 | 2.09 | -0.48 | -0.61 | -0.35 |
| Incidence | Guatemala | 7.24(7.58-6.94) | 5.78 | 8.73 | 1.51 | 195.62(225.54-169.31) | 3.90 | 8.78 | 2.25 | -0.98 | -1.32 | -0.63 |
| Incidence | Guinea | 8.36(9.42-7.34) | 4.15 | 12.51 | 3.02 | 352.72(469.58-230.23) | 4.00 | 14.64 | 3.66 | 0.85 | 0.77 | 0.94 |
| Incidence | Guinea-Bissau | 12.83(15.62-9.96) | 4.82 | 20.80 | 4.32 | 383.00(508.76-266.60) | 4.83 | 16.71 | 3.46 | -0.73 | -0.85 | -0.61 |
| Incidence | Guyana | 6.36(6.75-5.99) | 3.70 | 9.28 | 2.51 | 223.77(263.93-190.32) | 5.15 | 9.43 | 1.83 | 0.45 | 0.16 | 0.74 |
| Incidence | Haiti | 15.72(21.09-12.56) | 7.12 | 24.56 | 3.45 | 435.58(684.04-304.08) | 6.43 | 18.57 | 2.89 | -1.05 | -1.23 | -0.87 |
| Incidence | Honduras | 7.30(8.24-6.44) | 4.42 | 10.29 | 2.33 | 230.59(301.53-172.34) | 5.35 | 10.21 | 1.91 | -0.42 | -0.51 | -0.34 |
| Incidence | Hungary | 45.73(47.04-44.49) | 16.86 | 84.62 | 5.02 | 1631.33(1777.79-1500.53) | 29.14 | 67.88 | 2.33 | -1.15 | -1.54 | -0.77 |
| Incidence | Iceland | 33.55(35.43-31.76) | 27.86 | 40.45 | 1.45 | 630.58(679.77-584.52) | 30.38 | 38.24 | 1.26 | -1.37 | -1.57 | -1.17 |
| Incidence | India | 6.93(7.61-6.29) | 2.84 | 10.88 | 3.84 | 259.28(280.60-236.75) | 4.21 | 11.15 | 2.65 | 0.03 | -0.17 | 0.23 |
| Incidence | Indonesia | 15.46(17.98-13.41) | 7.95 | 23.68 | 2.98 | 649.22(745.08-559.25) | 8.13 | 28.35 | 3.49 | 0.39 | 0.32 | 0.46 |
| Incidence | Iran | 9.65(10.71-8.98) | 4.82 | 14.32 | 2.97 | 331.43(351.56-315.70) | 6.49 | 14.78 | 2.28 | 0.48 | 0.08 | 0.88 |
| Incidence | Iraq | 16.57(19.76-13.83) | 6.48 | 27.04 | 4.17 | 295.51(330.19-265.27) | 5.35 | 12.81 | 2.40 | -3.25 | -3.57 | -2.92 |
| Incidence | Ireland | 35.61(36.75-34.55) | 21.04 | 53.68 | 2.55 | 626.05(694.10-564.54) | 27.48 | 36.93 | 1.34 | -2.33 | -2.50 | -2.15 |
| Incidence | Israel | 19.89(20.60-19.20) | 11.40 | 29.93 | 2.63 | 601.75(659.95-547.71) | 12.05 | 27.94 | 2.32 | -0.91 | -1.17 | -0.66 |
| Incidence | Italy | 35.25(36.42-34.21) | 10.40 | 67.08 | 6.45 | 736.86(804.86-676.44) | 16.88 | 45.43 | 2.69 | -2.83 | -2.93 | -2.73 |
| Incidence | Jamaica | 14.16(15.10-13.22) | 5.82 | 23.58 | 4.05 | 772.22(936.49-631.87) | 7.12 | 32.02 | 4.49 | -0.69 | -0.90 | -0.49 |
| Incidence | Japan | 27.38(27.71-27.05) | 13.35 | 47.21 | 3.54 | 590.18(613.76-564.89) | 14.42 | 46.55 | 3.23 | 0.41 | -0.19 | 1.02 |
| Incidence | Jordan | 12.21(14.28-9.45) | 4.01 | 20.14 | 5.02 | 402.66(479.08-339.70) | 5.36 | 17.93 | 3.35 | -0.87 | -1.03 | -0.71 |
| Incidence | Kazakhstan | 35.83(37.02-34.62) | 10.70 | 74.38 | 6.95 | 881.87(964.30-809.88) | 6.24 | 36.02 | 5.77 | -0.37 | -0.78 | 0.04 |
| Incidence | Kenya | 6.28(8.62-5.18) | 3.16 | 9.40 | 2.98 | 217.61(319.78-183.23) | 2.69 | 9.89 | 3.68 | -3.32 | -3.56 | -3.09 |
| Incidence | Kiribati | 15.48(17.02-14.04) | 9.94 | 22.80 | 2.29 | 578.22(724.02-468.48) | 9.69 | 23.88 | 2.46 | 0.05 | -0.23 | 0.34 |
| Incidence | Kuwait | 10.09(10.79-9.44) | 5.39 | 13.18 | 2.44 | 197.77(220.53-175.14) | 3.12 | 9.38 | 3.01 | 0.05 | -0.14 | 0.24 |
| Incidence | Kyrgyzstan | 22.88(24.33-21.51) | 7.26 | 45.28 | 6.24 | 472.97(509.97-435.95) | 4.69 | 19.14 | 4.08 | -1.24 | -1.52 | -0.95 |
| Incidence | Laos | 25.03(31.03-19.91) | 11.80 | 39.72 | 3.37 | 725.80(910.82-565.01) | 11.10 | 30.88 | 2.78 | -3.23 | -3.63 | -2.83 |
| Incidence | Latvia | 33.16(34.25-32.15) | 8.56 | 73.58 | 8.60 | 1156.77(1316.57-1002.17) | 11.08 | 61.09 | 5.52 | -1.31 | -1.37 | -1.25 |
| Incidence | Lebanon | 25.44(29.52-21.31) | 10.26 | 43.62 | 4.25 | 670.59(819.82-579.69) | 18.07 | 31.26 | 1.73 | -1.93 | -2.26 | -1.61 |
| Incidence | Lesotho | 12.44(17.40-10.12) | 4.40 | 22.93 | 5.21 | 780.84(1021.53-595.68) | 6.06 | 30.11 | 4.97 | -1.57 | -1.72 | -1.42 |
| Incidence | Liberia | 8.29(11.04-6.87) | 3.98 | 11.79 | 2.96 | 236.84(348.10-172.62) | 3.68 | 10.70 | 2.91 | 1.51 | 1.21 | 1.82 |
| Incidence | Libya | 20.93(25.29-17.26) | 4.07 | 36.21 | 8.89 | 842.32(1020.81-669.86) | 5.02 | 35.49 | 7.07 | -0.20 | -0.47 | 0.06 |
| Incidence | Lithuania | 32.26(33.27-31.26) | 7.56 | 70.00 | 9.26 | 1193.38(1307.83-1093.94) | 9.77 | 61.55 | 6.30 | 0.00 | -0.10 | 0.09 |
| Incidence | Luxembourg | 37.94(39.81-36.11) | 11.54 | 74.51 | 6.46 | 865.38(989.83-753.23) | 24.59 | 50.45 | 2.05 | -1.79 | -2.02 | -1.55 |
| Incidence | Macedonia | 22.89(24.25-21.56) | 6.67 | 40.56 | 6.09 | 1292.05(1457.84-1147.01) | 9.92 | 51.06 | 5.15 | -2.71 | -2.84 | -2.57 |
| Incidence | Madagascar | 7.93(8.99-6.90) | 4.02 | 11.40 | 2.84 | 246.70(320.43-181.76) | 3.30 | 10.58 | 3.20 | 0.63 | 0.39 | 0.87 |
| Incidence | Malawi | 5.84(7.04-3.92) | 3.23 | 8.71 | 2.70 | 200.68(243.13-162.34) | 2.65 | 8.93 | 3.37 | -0.25 | -0.38 | -0.11 |
| Incidence | Malaysia | 18.29(20.10-16.77) | 8.55 | 29.04 | 3.40 | 592.30(693.50-494.57) | 10.27 | 26.68 | 2.60 | -0.12 | -0.44 | 0.20 |
| Incidence | Maldives | 12.96(16.19-10.27) | 3.39 | 19.14 | 5.64 | 230.08(260.44-201.82) | 2.59 | 10.56 | 4.07 | -0.57 | -0.71 | -0.44 |
| Incidence | Mali | 7.00(8.00-6.16) | 3.79 | 10.13 | 2.67 | 206.14(278.96-156.68) | 3.75 | 9.21 | 2.45 | -2.97 | -3.15 | -2.80 |
| Incidence | Malta | 24.43(25.85-23.14) | 5.73 | 48.09 | 8.40 | 769.73(833.20-701.94) | 9.33 | 46.49 | 4.98 | -0.48 | -0.63 | -0.33 |
| Incidence | Marshall Islands | 24.31(33.46-14.77) | 7.77 | 41.97 | 5.40 | 1023.83(1552.37-619.39) | 10.17 | 43.13 | 4.24 | -1.49 | -1.62 | -1.37 |
| Incidence | Mauritania | 9.95(11.89-8.17) | 5.61 | 14.82 | 2.64 | 270.12(376.34-198.67) | 5.56 | 12.66 | 2.27 | -0.01 | -0.10 | 0.09 |
| Incidence | Mauritius | 12.55(13.20-11.87) | 5.33 | 22.06 | 4.14 | 395.77(435.97-357.94) | 5.61 | 17.97 | 3.20 | -0.68 | -0.97 | -0.39 |
| Incidence | Mexico | 12.50(12.70-12.32) | 7.22 | 18.36 | 2.54 | 248.26(256.87-239.28) | 4.99 | 11.74 | 2.35 | -0.92 | -1.03 | -0.81 |
| Incidence | Moldova | 25.51(26.50-24.70) | 8.01 | 49.50 | 6.18 | 921.38(991.11-853.53) | 6.35 | 35.97 | 5.66 | -2.29 | -2.43 | -2.15 |
| Incidence | Mongolia | 35.80(39.32-32.79) | 16.41 | 60.45 | 3.68 | 1011.40(1166.16-858.64) | 10.25 | 45.57 | 4.44 | 0.01 | -0.02 | 0.04 |
| Incidence | Montenegro | 40.70(44.08-37.73) | 16.61 | 71.31 | 4.29 | 1658.49(1930.12-1418.64) | 21.02 | 69.81 | 3.32 | -1.17 | -1.66 | -0.68 |
| Incidence | Morocco | 12.95(15.22-11.15) | 3.08 | 22.89 | 7.42 | 584.31(744.66-448.30) | 3.36 | 23.48 | 6.99 | -1.73 | -2.00 | -1.46 |
| Incidence | Mozambique | 8.56(9.91-7.42) | 3.19 | 14.58 | 4.58 | 406.16(497.90-326.63) | 3.24 | 17.72 | 5.46 | -0.35 | -0.52 | -0.17 |
| Incidence | Myanmar | 22.23(28.44-17.38) | 13.05 | 32.62 | 2.50 | 647.16(748.13-548.07) | 11.17 | 26.43 | 2.37 | -0.07 | -0.24 | 0.09 |
| Incidence | Namibia | 8.37(9.34-7.47) | 4.47 | 13.17 | 2.94 | 263.82(306.65-227.18) | 3.67 | 11.63 | 3.17 | 1.23 | 1.06 | 1.39 |
| Incidence | Nepal | 11.01(16.63-6.57) | 7.82 | 14.02 | 1.79 | 297.26(479.68-215.83) | 8.00 | 13.00 | 1.63 | -0.82 | -0.90 | -0.75 |
| Incidence | Netherlands | 45.95(47.91-44.32) | 13.64 | 90.56 | 6.64 | 884.33(964.01-812.23) | 37.08 | 54.24 | 1.46 | -0.94 | -1.23 | -0.64 |
| Incidence | New Zealand | 34.76(36.68-33.10) | 21.21 | 52.64 | 2.48 | 541.95(589.53-497.56) | 27.52 | 35.43 | 1.29 | -0.51 | -0.93 | -0.09 |
| Incidence | Nicaragua | 6.43(7.06-5.87) | 3.96 | 9.16 | 2.31 | 178.72(210.84-151.59) | 3.99 | 8.05 | 2.02 | -2.93 | -3.02 | -2.84 |
| Incidence | Niger | 8.11(10.74-6.01) | 4.13 | 11.73 | 2.84 | 256.87(368.25-185.22) | 4.09 | 11.54 | 2.82 | -2.68 | -2.79 | -2.57 |
| Incidence | Nigeria | 7.97(10.42-5.93) | 3.64 | 12.31 | 3.39 | 236.88(328.26-175.98) | 4.35 | 11.12 | 2.56 | -0.55 | -0.73 | -0.38 |
| Incidence | North Korea | 22.81(26.99-18.92) | 12.42 | 41.08 | 3.31 | 991.68(1305.22-754.61) | 10.85 | 41.19 | 3.80 | -0.15 | -0.39 | 0.09 |
| Incidence | Northern Mariana Islands | 36.76(45.27-30.47) | 16.92 | 53.02 | 3.13 | 979.74(1128.76-835.62) | 14.18 | 45.66 | 3.22 | -0.40 | -0.67 | -0.14 |
| Incidence | Norway | 27.85(28.43-27.31) | 15.26 | 43.82 | 2.87 | 541.84(571.57-516.11) | 25.73 | 36.20 | 1.41 | 0.07 | 0.00 | 0.15 |
| Incidence | Oman | 7.74(9.65-6.18) | 3.01 | 12.13 | 4.02 | 193.15(243.19-149.49) | 3.57 | 9.08 | 2.55 | -0.64 | -0.79 | -0.48 |
| Incidence | Pakistan | 11.96(14.35-10.51) | 4.80 | 17.84 | 3.72 | 500.82(642.39-382.18) | 5.88 | 20.15 | 3.43 | -1.62 | -1.87 | -1.37 |
| Incidence | Palestine | 16.48(20.14-13.27) | 6.50 | 27.98 | 4.30 | 512.96(577.19-458.46) | 7.29 | 21.44 | 2.94 | -1.06 | -1.26 | -0.87 |
| Incidence | Panama | 12.58(13.19-12.01) | 5.82 | 19.23 | 3.30 | 266.30(294.65-238.29) | 6.04 | 12.96 | 2.15 | 0.26 | -0.01 | 0.52 |
| Incidence | Papua New Guinea | 24.70(33.53-20.10) | 11.96 | 36.44 | 3.05 | 963.64(1485.16-707.57) | 13.20 | 38.23 | 2.90 | -1.18 | -1.37 | -1.00 |
| Incidence | Paraguay | 10.47(11.43-9.48) | 6.12 | 15.40 | 2.52 | 495.56(624.07-390.70) | 6.73 | 21.92 | 3.26 | -2.19 | -2.36 | -2.03 |
| Incidence | Peru | 12.09(13.33-10.89) | 8.52 | 15.92 | 1.87 | 228.04(278.61-181.31) | 7.69 | 10.26 | 1.33 | 0.14 | 0.10 | 0.18 |
| Incidence | Philippines | 15.73(16.70-14.73) | 7.92 | 24.15 | 3.05 | 640.64(758.71-538.44) | 9.81 | 27.04 | 2.76 | 1.28 | 0.83 | 1.74 |
| Incidence | Poland | 40.02(40.95-39.06) | 12.13 | 77.44 | 6.38 | 1403.00(1512.80-1288.55) | 20.03 | 60.31 | 3.01 | -2.08 | -2.46 | -1.69 |
| Incidence | Portugal | 18.01(18.55-17.44) | 6.24 | 33.20 | 5.32 | 776.25(852.31-705.48) | 8.86 | 32.32 | 3.65 | -0.37 | -0.70 | -0.04 |
| Incidence | Puerto Rico | 14.38(14.92-13.84) | 8.19 | 21.62 | 2.64 | 311.79(343.59-281.29) | 7.31 | 15.49 | 2.12 | -1.47 | -1.63 | -1.32 |
| Incidence | Qatar | 19.09(22.70-15.95) | 6.96 | 27.95 | 4.02 | 312.22(385.19-245.31) | 6.09 | 15.86 | 2.60 | -0.16 | -0.40 | 0.08 |
| Incidence | Romania | 23.53(24.28-22.79) | 7.22 | 42.28 | 5.86 | 1301.60(1405.09-1203.52) | 11.63 | 51.57 | 4.44 | -2.03 | -2.18 | -1.87 |
| Incidence | Russia | 33.08(34.20-32.10) | 8.55 | 75.85 | 8.87 | 1078.57(1102.49-1055.54) | 8.59 | 54.52 | 6.35 | -2.45 | -2.88 | -2.01 |
| Incidence | Rwanda | 9.38(11.71-6.79) | 3.80 | 16.00 | 4.21 | 262.05(331.82-196.38) | 3.44 | 11.95 | 3.48 | -1.81 | -2.03 | -1.59 |
| Incidence | Saint Lucia | 11.66(12.41-10.95) | 6.71 | 18.09 | 2.69 | 329.97(367.88-292.99) | 7.14 | 13.98 | 1.96 | 0.08 | -0.11 | 0.28 |
| Incidence | Saint Vincent | 8.36(8.97-7.75) | 5.35 | 12.20 | 2.28 | 297.32(331.44-266.90) | 6.27 | 12.29 | 1.96 | -2.42 | -2.75 | -2.09 |
| Incidence | Grenadines | 8.36(8.97-7.75) | 5.35 | 12.20 | 2.28 | 297.32(331.44-266.90) | 6.27 | 12.29 | 1.96 | -1.88 | -2.14 | -1.61 |
| Incidence | Samoa | 7.65(8.81-6.50) | 3.96 | 11.43 | 2.89 | 250.87(310.84-202.19) | 4.17 | 11.01 | 2.64 | -1.03 | -1.25 | -0.82 |
| Incidence | Sao Tome and Principe | 11.53(13.27-10.24) | 4.93 | 19.61 | 3.97 | 540.47(691.72-398.36) | 6.14 | 24.48 | 3.99 | 0.07 | -0.08 | 0.22 |
| Incidence | Saudi Arabia | 7.57(9.39-6.05) | 2.25 | 11.57 | 5.15 | 268.84(321.44-224.77) | 3.23 | 13.36 | 4.14 | -0.42 | -0.47 | -0.36 |
| Incidence | Senegal | 11.41(14.57-9.42) | 4.61 | 17.99 | 3.90 | 379.59(507.13-286.78) | 4.75 | 17.34 | 3.65 | 0.94 | 0.80 | 1.09 |
| Incidence | Serbia | 39.25(42.86-36.38) | 14.13 | 68.29 | 4.83 | 1521.73(1696.94-1376.31) | 25.52 | 63.30 | 2.48 | 0.68 | 0.35 | 1.00 |
| Incidence | Seychelles | 14.92(16.27-13.56) | 5.46 | 27.16 | 4.98 | 559.65(621.89-498.78) | 6.49 | 24.09 | 3.71 | -0.05 | -0.32 | 0.23 |
| Incidence | Sierra Leone | 9.96(13.68-7.37) | 4.68 | 14.86 | 3.18 | 318.52(446.27-234.92) | 4.73 | 14.42 | 3.05 | -0.14 | -0.34 | 0.06 |
| Incidence | Singapore | 35.46(36.79-34.24) | 18.61 | 55.72 | 2.99 | 538.37(592.02-490.76) | 14.85 | 38.55 | 2.60 | -1.32 | -1.65 | -1.00 |
| Incidence | Slovakia | 44.21(46.23-42.41) | 9.69 | 89.21 | 9.21 | 992.23(1112.66-884.02) | 15.75 | 61.88 | 3.93 | -0.01 | -0.13 | 0.12 |
| Incidence | Slovenia | 34.35(35.48-33.29) | 10.14 | 69.49 | 6.85 | 944.32(1033.93-854.67) | 17.35 | 50.68 | 2.92 | -3.13 | -3.32 | -2.93 |
| Incidence | Solomon Islands | 22.54(30.77-17.61) | 10.30 | 31.90 | 3.10 | 715.08(1084.33-539.37) | 10.30 | 29.63 | 2.88 | -2.38 | -2.46 | -2.30 |
| Incidence | Somalia | 8.49(11.40-5.81) | 3.92 | 13.08 | 3.34 | 283.49(416.55-202.49) | 3.49 | 12.35 | 3.54 | -2.49 | -2.68 | -2.29 |
| Incidence | South Africa | 17.51(21.89-15.88) | 9.43 | 28.07 | 2.98 | 631.77(674.07-593.36) | 8.50 | 25.75 | 3.03 | -0.44 | -0.46 | -0.42 |
| Incidence | South Korea | 20.08(20.65-19.51) | 9.00 | 36.25 | 4.03 | 688.46(764.20-618.49) | 15.66 | 51.86 | 3.31 | -0.57 | -0.73 | -0.41 |
| Incidence | South Sudan | 9.90(13.58-6.80) | 4.71 | 14.05 | 2.98 | 326.80(442.95-238.59) | 4.22 | 14.25 | 3.38 | -0.90 | -1.55 | -0.25 |
| Incidence | Spain | 30.97(31.87-30.09) | 5.33 | 63.37 | 11.90 | 931.49(1009.29-855.66) | 13.34 | 56.36 | 4.23 | -1.32 | -1.90 | -0.74 |
| Incidence | Sri Lanka | 7.15(7.75-6.60) | 3.51 | 10.73 | 3.05 | 254.48(321.92-199.78) | 3.47 | 11.00 | 3.17 | -0.13 | -0.26 | 0.00 |
| Incidence | Sudan | 8.25(12.47-6.16) | 3.78 | 12.27 | 3.25 | 251.15(367.12-178.07) | 3.84 | 11.01 | 2.86 | -1.29 | -1.48 | -1.11 |
| Incidence | Suriname | 11.88(12.80-11.02) | 7.15 | 17.18 | 2.40 | 453.20(524.54-384.73) | 9.03 | 19.25 | 2.13 | 0.44 | 0.13 | 0.77 |
| Incidence | Swaziland | 16.33(20.29-13.04) | 6.70 | 28.77 | 4.29 | 707.35(962.61-385.72) | 6.14 | 27.37 | 4.46 | -0.43 | -0.48 | -0.37 |
| Incidence | Sweden | 20.50(21.12-19.99) | 13.52 | 29.36 | 2.17 | 372.69(401.57-344.29) | 20.86 | 20.77 | 1.00 | 0.53 | 0.27 | 0.79 |
| Incidence | Switzerland | 30.54(32.25-29.26) | 10.84 | 56.26 | 5.19 | 548.16(605.03-492.62) | 19.13 | 31.90 | 1.67 | 0.44 | -0.03 | 0.92 |
| Incidence | Syria | 9.09(10.38-7.95) | 4.24 | 13.49 | 3.18 | 328.38(414.06-253.53) | 4.19 | 13.92 | 3.32 | -1.73 | -1.89 | -1.57 |
| Incidence | Taiwan (Province of China) | 22.64(23.19-22.08) | 14.03 | 30.27 | 2.16 | 737.90(800.19-677.64) | 21.72 | 40.70 | 1.87 | -2.73 | -2.88 | -2.59 |
| Incidence | Tajikistan | 12.57(13.25-11.90) | 6.45 | 20.07 | 3.11 | 259.81(308.11-211.21) | 4.40 | 10.97 | 2.49 | 0.05 | -0.20 | 0.30 |
| Incidence | Tanzania | 7.08(8.40-5.74) | 3.48 | 10.76 | 3.10 | 232.63(285.38-184.89) | 3.51 | 10.51 | 2.99 | 0.34 | 0.10 | 0.58 |
| Incidence | Thailand | 28.39(30.15-26.55) | 20.70 | 37.31 | 1.80 | 640.01(748.50-553.79) | 11.37 | 28.45 | 2.50 | -2.24 | -3.01 | -1.46 |
| Incidence | Bahamas | 15.04(16.17-13.99) | 6.46 | 26.10 | 4.04 | 464.68(531.68-404.12) | 7.46 | 19.32 | 2.59 | -0.29 | -0.42 | -0.15 |
| Incidence | Gambia | 7.57(9.06-6.21) | 3.65 | 11.09 | 3.04 | 254.00(310.03-198.20) | 3.93 | 11.28 | 2.87 | -1.84 | -2.03 | -1.64 |
| Incidence | Timor-Leste | 15.44(20.44-12.20) | 8.93 | 21.71 | 2.43 | 596.80(889.62-423.78) | 9.01 | 26.14 | 2.90 | 0.58 | 0.37 | 0.79 |
| Incidence | Togo | 8.86(10.17-7.61) | 4.50 | 13.54 | 3.01 | 337.63(435.93-258.29) | 4.15 | 14.94 | 3.60 | -0.62 | -0.78 | -0.47 |
| Incidence | Tonga | 27.81(30.69-25.21) | 12.25 | 44.11 | 3.60 | 970.43(1165.03-817.31) | 10.71 | 42.53 | 3.97 | 0.49 | 0.37 | 0.61 |
| Incidence | Trinidad | 10.74(11.30-10.18) | 4.91 | 17.26 | 3.52 | 356.40(452.33-273.08) | 4.88 | 14.65 | 3.01 | 0.08 | 0.04 | 0.11 |
| Incidence | Tobago | 10.74(11.30-10.18) | 4.91 | 17.26 | 3.52 | 356.40(452.33-273.08) | 4.88 | 14.65 | 3.01 | -0.62 | -0.78 | -0.47 |
| Incidence | Tunisia | 17.35(19.58-15.28) | 2.78 | 30.69 | 11.04 | 690.27(893.20-518.07) | 3.60 | 31.53 | 8.76 | -0.27 | -0.50 | -0.03 |
| Incidence | Turkey | 38.48(42.81-33.39) | 9.44 | 70.29 | 7.45 | 1346.88(1518.30-1175.60) | 7.93 | 57.61 | 7.27 | -0.94 | -1.13 | -0.75 |
| Incidence | Turkmenistan | 13.39(13.98-12.81) | 5.10 | 24.22 | 4.75 | 413.34(462.92-366.92) | 4.45 | 16.27 | 3.65 | -2.17 | -2.67 | -1.67 |
| Incidence | Uganda | 5.31(6.35-4.44) | 3.60 | 6.89 | 1.91 | 219.95(263.65-176.78) | 3.46 | 9.80 | 2.83 | 1.08 | 0.79 | 1.36 |
| Incidence | Ukraine | 35.74(37.63-34.12) | 10.21 | 76.32 | 7.47 | 1116.09(1211.48-1031.43) | 8.03 | 56.22 | 7.00 | -2.78 | -2.91 | -2.65 |
| Incidence | United Arab Emirates | 13.66(16.84-10.99) | 6.30 | 19.16 | 3.04 | 439.99(570.51-331.81) | 7.30 | 20.65 | 2.83 | -2.40 | -2.74 | -2.06 |
| Incidence | UK | 46.02(46.72-45.34) | 27.19 | 73.13 | 2.69 | 694.98(711.13-677.86) | 34.47 | 46.35 | 1.34 | 0.31 | 0.14 | 0.48 |
| Incidence | USA | 54.05(54.74-53.35) | 35.81 | 78.92 | 2.20 | 854.61(885.60-823.43) | 37.80 | 53.86 | 1.42 | -1.66 | -1.75 | -1.56 |
| Incidence | Uruguay | 36.16(37.42-34.89) | 8.45 | 70.31 | 8.32 | 1162.52(1331.39-999.66) | 13.74 | 49.29 | 3.59 | -2.60 | -2.73 | -2.46 |
| Incidence | Uzbekistan | 14.07(14.57-13.62) | 6.36 | 24.28 | 3.82 | 413.94(483.18-356.63) | 4.47 | 17.84 | 3.99 | -1.55 | -1.92 | -1.18 |
| Incidence | Vanuatu | 24.25(32.81-18.77) | 12.02 | 34.03 | 2.83 | 823.67(1239.31-593.73) | 11.97 | 34.40 | 2.87 | 0.02 | -0.03 | 0.07 |
| Incidence | Venezuela | 16.23(16.78-15.67) | 10.81 | 22.38 | 2.07 | 505.02(612.87-416.64) | 11.67 | 22.01 | 1.89 | -0.23 | -0.38 | -0.07 |
| Incidence | Vietnam | 33.41(38.17-29.14) | 17.30 | 55.96 | 3.24 | 1499.02(1791.74-1258.73) | 19.93 | 63.87 | 3.20 | 0.54 | 0.43 | 0.65 |
| Incidence | Virgin Islands | 17.26(18.94-15.76) | 9.58 | 26.57 | 2.78 | 724.12(850.40-556.44) | 12.12 | 31.71 | 2.62 | 0.67 | 0.58 | 0.77 |
| Incidence | Yemen | 10.25(15.49-6.54) | 4.70 | 16.15 | 3.44 | 360.73(521.61-260.80) | 4.40 | 15.59 | 3.55 | -0.19 | -0.24 | -0.14 |
| Incidence | Zambia | 9.36(10.93-7.94) | 3.96 | 13.85 | 3.49 | 281.43(329.88-230.06) | 3.58 | 12.87 | 3.60 | -0.75 | -0.95 | -0.55 |
| Incidence | Zimbabwe | 11.57(13.27-9.99) | 4.93 | 18.75 | 3.80 | 401.21(490.50-318.99) | 5.79 | 16.13 | 2.78 | -0.33 | -0.90 | 0.25 |

ASDR, age standardized death rate; ASIR, age standardized incidence rate; DALY, disability adjusted life-year; EAPC: estimated annual percentage change; CI: confidence interval; UI: uncertainty interval.
